# POU6F1 cooperates with RORA to suppress the proliferation of lung adenocarcinoma by downregulating HIF1A signaling pathway

**DOI:** 10.1038/s41419-022-04857-y

**Published:** 2022-05-03

**Authors:** Wenjing Xiao, Wei Geng, Mei Zhou, Juanjuan Xu, Sufei Wang, Qi Huang, Yice Sun, Yumei Li, Guanghai Yang, Yang Jin

**Affiliations:** 1 Department of Respiratory and Critical Care Medicine, NHC Key Laboratory of Pulmonary Diseases, Union Hospital, Tongji Medical College, Huazhong University of Science and Technology, 1277 Jiefang Avenue, Wuhan, 430022 Hubei China; 2 Department of Thoracic Surgery, Union Hospital, Tongji Medical College, Huazhong University of Science and Technology, Wuhan, 430022 China

**Keywords:** Non-small-cell lung cancer, Translational research

## Abstract

Lung adenocarcinoma (LUAD) represents the most frequently diagnosed histological subtype of non-small cell lung cancer with the highest mortality worldwide. Transcriptional dysregulation is a hallmark of nearly all kinds of cancers. In the study, we identified that the POU domain, class 6, transcription factor 1 (POU6F1), a member of the POU family of transcription factors, was closely associated with tumor stage and death in LUAD. We revealed that POU6F1 was downregulated in LUAD tissues and downregulated POU6F1 was predictive of an unfavorable prognosis in LUAD patients. In vitro assays, including CCK8, soft agar, transwell, clone formation, wound-healing assay, and nude mouse xenograft model all revealed that POU6F1 inhibited the growth and invasion of LUAD cells. Mechanistically, POU6F1 bound and stabilized retinoid-related orphan receptor alpha (RORA) to exert the transcriptional inhibition of hypoxia-inducible factor 1-alpha (HIF1A) and alter the expression of HIF1A signaling pathway-associated genes, including ENO1, PDK1, and PRKCB, thereby leading to the suppression of LUAD cells. Collectively, these results demonstrated the suppressive role of POU6F1/RORA in the progression of LUAD and may potentially be used as a target for the treatment of LUAD.

## Introduction

Lung cancer is believed to be the leading cause of malignancies-related deaths globally, with 85% being non-small cell lung cancer (NSCLC) [1, 2]. Lung adenocarcinoma (LUAD), the most predominant histological subtype of NSCLC, is usually diagnosed at an advanced stage with a survival rate being as low as 15% and a high postoperative recurrence rate, despite great advances in molecular diagnostics, targeted therapies, and immunotherapies [3, 4]. Hence, unveiling critical regulatory molecules is of paramount importance for understanding the molecular mechanisms of LUAD carcinogenesis and developing more efficacious therapeutic strategies.

The POU family of transcription factors play vitally important roles in determining cell-fate determination and timing of cellular events [5]. Among them, the POU domain, class 6, transcription factor 1 (POU6F1), known as BRN5, MPOU, and TCFB1 displayed tissue specificity [6]. It is expressed exclusively in the developing brain in the embryo. However, it is restricted to the brain, heart, and skeletal muscle of adults, intimately involved in the neurons and heart development [6–8]. Moreover, it has been implicated in carcinogenesis. For instance, POU6F1 siRNA dose-dependently suppressed tumor proliferation in clear cell adenocarcinoma cell lines [9, 10]. Nonetheless, so far, the role of POU6F1 has never been studied in LUAD.

Retinoid-related orphan receptor alpha (RORA), a member of orphan nuclear receptors (ONRs), has been proved to be associated with multiple biological processes and diseases, such as lipid metabolism [11], osteoarthritis [12], circadian rhythm [13], and cancers [14, 15]. Reduced RORA expression was associated with tumor progression and unfavorable prognosis in cancers, such as prostate cancer [16, 17], colon [14], and breast cancer [15]. For instance, RORA suppressed cell proliferation by down-regulating the Wnt/β-catenin pathway in gastric cancer and colon cancer [14, 18]. RORA has been found to affect transcriptional activity of HIF1A and participate in the HIF1A signaling pathway [19, 20].

In the present study, we identified that POU6F1 was decreased in LUAD tissues and POU6F1 downregulation was indicative of poor prognosis of LUAD patients. We revealed that POU6F1 inhibited the growth and invasion of LUAD and found that POU6F1 was involved in the HIF1A signaling pathway. Therefore, we explored whether POU6F1 coordinated with RORA to participate in the HIF1A signaling pathway.

## Materials and methods

### Cell culture

Human A549, H1975, SPC, PC-9, NCI-H1299, human bronchial epithelial cells (HBE), and embryonic kidney HEK293T were obtained from Shanghai Institutes for Biological Science, China, authenticated by short tandem repeat profiling and used within 6 months after resuscitation of frozen aliquots. All lung adenocarcinoma (LUAD) cell lines and HBE were cultured in RPMI 1640 medium and HEK293T was cultured in DMEM medium, supplemented with 10% fetal bovine serum and 1% penicillin–streptomycin (Beyotime, Shanghai, China, C0222) at a 37 °C humidified incubator containing 5% CO_2_.

### Data mining of public dataset

A comprehensive analysis in a public LUAD dataset (TCGA-LUAD) of R2: Genomics Analysis and Visualization Platform (amc.nl) (https://hgserver2.amc.nl/cgi-bin/r2/main.cgi) containing 515 cases was performed to identify potential transcription factors (TFs) that were differentially expressed with the death, clinical progression, and metastasis, respectively. A detailed list of the screened TFs was displayed in Supplementary Tables 1–3.

### Gene expression analysis

The transcript information (fragments per kilobase million, FPKM) of 594 LUAD patients, involving 535 LUAD tissues and 59 normal lung tissues, was downloaded from The Cancer Genome Atlas (TCGA). POU6F1 transcription levels in pan-cancer were evaluated from Oncomine (https://www.oncomine.org/) [21] and TIMER: Tumor IMmune Estimation Resource (https://cistrome.shinyapps.io/timer/) [22].

### Human tissue samples

Paired LUAD tissue samples and adjacent normal tissue samples were collected from patients who had never received preoperative chemotherapy or other treatments and undergone surgical resection in Wuhan Union Hospital of Tongji Medical College, Wuhan, China. The Institutional Review Board of Tongji Medical College approved all human specimen studies ([2010]IEC(S202)) and written informed consent was obtained from patients or legal guardians of patients. All tissues were validated by pathological diagnosis and frozen in liquid nitrogen for future use.

### Western blotting

All cellular and tissue proteins were extracted with 1 × cell lysis buffer (Thermo Fisher Scientific, Inc., Waltham, MA, USA) and a protease inhibitor cocktail (Beyotime, Shanghai, China). The protein concentration of the lysate was measured using a BCA protein assay kit (Beyotime, Shanghai, China). Then, the lysate was diluted in 5× SDS-PAGE loading buffer and then boiled for 10 min at 95 °C. Cell lysate containing 30 μg protein was loaded into appropriate gels for separation based on the molecular weight of protein and then transferred to PVDF membranes (Millipore, Billerica, MA, USA). Thereafter, membranes were blocked with 5% skimmed milk powder and incubated with the primary antibodies specific for POU6F1 (abclonal, A7299), RORA (Proteintech, 10616-1-AP; Santa Cruz Biotechnology, sc-518081), HIF1A (Abcam, ab51608), actin (Abclonal, AC026), ENO1 (Proteintech, 11204-1-AP), ENO2 (Proteintech, 66150-1-Ig), PDK1 (Proteintech, 10026-1-AP), PRKCB (Proteintech, 12919-1-AP), HA (Abcam, ab9110), Histone-H3 (Proteintech, 17168-1-AP), Flag antibody (Abcam, ab205606), and GAPDH (Abcam, ab8245) at 4 °C overnight. After being washed with TBST three times for 15 min, membranes were further incubated with the secondary antibody for 1 h at room temperature. After being washed with TBST three times for 15 min, proteins were visualized using an ECL chemiluminescence staining assay kit and revealed on a ChemiDoc Touch Imaging System (Bio-Rad).

### RNA extraction and real‑time quantitative PCR

Whole cellular and tissue RNAs were extracted using TRIzol reagent (Invitrogen, Carlsbad, CA, USA) according to the manufacturer’s instructions. The obtained RNA was diluted in diethylpyrocarbonate and the concentration of RNA was measured with the NanoDrop 2000 (NanoDrop Technologies, Wilmington, USA). For real-time PCR (RT-PCR), 1 µg of RNA was reverse transcribed into cDNA using HisScript Reverse Transcriptase (Vazyme Biotech Co., Ltd), and quantification of mRNA was performed using SYBR Green PCR Master Mix (Vazyme Biotech Co., Ltd). The corresponding primer sequences were listed in Supplementary Table 4. The relative expression of genes was normalized to β-actin and determined by the 2^−△△Ct^ method.

### Ectopic expression and knockdown of genes

Human POU6F1 cDNA (1836 bp) and RORA cDNA (1671 bp) were obtained by PCR using the vector LV201-POU6F1 and LV205-RORA (Guangzhou FulenGen Co., Ltd.) and then inserted into CV186 lentivirus vector containing sequence which could express both red fluorescent protein and anti-puromycin protein (Genechem Co., Ltd., Shanghai, China). The POU6F1 PCR fragments and pCMV-HA were double-digested with EcoR I and Xho I and ligated to construct pCMV-HA-POU6F1. Meanwhile, the pCMV-3Tag-1-RORA vector was constructed with BamH I and Hind III. In addition, independent single guide RNAs (sgRNAs) targeting the downstream region of the POU6F1 transcription start site and oligonucleotides specific of shRNAs for RORA were inserted into dCas9-BFP-KRAB (Addgene) and GV298 vector (Shanghai GeneChem Co., Ltd), respectively. The primer sequences were listed in Supplementary Table 5. Stable cell lines were constructed with puromycin for 3–4 weeks.

### RNA sequencing (RNA‑seq)

Total RNA was extracted from A549 cells stably transfected with POU6F1 overexpression plasmid or control plasmid with TRIzol reagent (Invitrogen, Carlsbad, CA, USA) and RNA‑seq was performed at Beijing Genomics institution (BGI-Shenzhen, China).

### Rescue of gene expression

To rescue gene expression resulting from POU6F1 knockdown, the overexpression plasmid of RORA was transfected into cancer cells with lipofectamine 3000 (Invitrogen). Briefly, we constructed four cell lines, including the cell line with CRISPRI-POU6F1 control vector and RORA-overexpression control vector, the cell line with CRISPRI-POU6F1 and RORA-overexpression control vector, the cell line with CRISPRI-POU6F1 control vector and RORA-overexpression vector, and the cell line with CRISPRI-POU6F1 and RORA-overexpression vector.

### Cell proliferation assay

CCK8 assay was used to detect cell proliferation viability according to the instructions (Beyotime, Shanghai, China), each experiment being independently performed at least three times. In brief, 2 × 10^3^ A549 and NCI-H1299 cells/well were plated in 96-well plates (eight wells per group) and then transfected with overexpression of POU6F1 or control plasmids. At 0 and 24 h, 90 μl of fresh serum-free medium and 10 μl of CCK8 reagent were mixed and added to each well. Then, absorbance was measured at a wavelength of 450 nm under a microplate reader 1 h later (Bio-Rad, Hercules, CA, USA).

### Colony formation assay

A549 and NCI-H1299 cells transfected with POU6F1 overexpression plasmid or control plasmid at a density of 500 per well were seeded into 12-well plates. The medium was changed every 3 days and incubated for about 3 weeks to allow for colony formation. Then, the cells were fixed with 4% paraformaldehyde for 20 min and stained with crystal violet for 30 min.

### Wound-healing assay

A549 and NCI-H1299 cells transfected with POU6F1 overexpression plasmid or control plasmid were seeded into 6‐well plates. When the density of cancer cells was allowed to reach 70–80% confluence, 200 μl pipette tips were employed to scratch a wound. At 0 h and 24 h, the wound closure was observed and images were photographed under a light microscope for detecting the migration ability of cells.

### Transwell assay

Transwell assays were performed to detect the migration of cancer cells with 8 μm-pore size transwell chambers (Corning, Inc.). Briefly, 2 × 10^4^ cancer cells suspended in 200 μl of serum-free medium were put in the upper chamber and 500 μl of the medium was placed in the lower chamber. Cancer cells were fixed with 4% paraformaldehyde for 20 min and stained with crystal violet. Lastly, the number of positive-stained cells was counted under a light microscope.

### Soft agar assay

The soft agar assay was conducted as previously described [23]. Briefly, the 3 ml mixture of 1.2% agar and 2× complete medium was placed in the 6-well plates, waiting to solidify at room temperature. Then, cancer cells at a density of 5000 were resuspended in a 1.5 ml semisolid culture medium, which was mixed with 2× complete medium and 0.6% agar, and placed upper on the solidified 6-well plates for 2 weeks. Colonies were stained with crystal violet and assessed.

### Bimolecular fluorescence complementation system (BiFC)

For BiFC assay, pBiFC-VC155-POU6F1 was constructed by ligating an EcoR I- and Kpn I- treated 1836 bp fragment into digested EcoR I- and Kpn I- treated pBIFC-VC155. pBIFC-VN173-RORA was constructed by ligating a Hind III- and Kpn I-treated 1671 bp fragment into digested Hind III- and Kpn I-treated pBiFC-VN173 (Addgene). The corresponding primers were listed in Supplementary Table 5. In brief, A549 cells were co-transfected with pBiFC-VC155-POU6F1 and pBiFC-VN173-RORA by using lipofectamine 3000 for 24 h. Then, the coverslips with seeded cells were photographed under a Nikon A1Si Laser Scanning Confocal Microscope (Nikon Instruments Inc, Japan) at the excitation and emission wavelengths of 488 nm and 500 nm, respectively.

### Cell cycle analysis

The cell cycle kit (Beyotime, Shanghai, China) was used for cell cycle distribution according to the manufacturer’s protocol. In a nutshell, A549 and NCI-H1299 cells were plated into 6-well plates and transfected with pCMV-HA-POU6F1 or empty vector (pCMV-HA). Cancer cells were harvested by trypsinization without EDTA, washed with PBS twice, and fixed with ice-cold 75% ethanol for at least 30 min at 4 °C. Then, the cells were stained with propidium iodide (50 µg/ml) and RNase (100 µg/ml) for 15 min. Lastly, cells were performed with BD LSRFortessa^TM^X-20 Special Order Product (Becton Dickinson, USA) and analyzed with FlowJo software (version 10).

### Retroviral cell cycle reporter indicator

A549 cells were seeded on coverslips and transfected with pCMV-HA-POU6F1 or pCMV-HA, and those co-transfected with pRetroX-G1-Red (Clontech, 631463) and pRetroX-SG2M-Cyan (Clontech, 631462). Then, coverslips were fixed with paraformaldehyde, stained with 4',6-diamidino-2-phenylindole (DAPI, 300 nmol/l), and photographed under a Nikon A1Si Laser Scanning Confocal Microscope (Nikon Instruments Inc, Japan).

### Co‐immunoprecipitation (Co-IP) and mass spectrometry (MS)

Co‐IP and MS were carried out in A549 and HEK293T cells. Cells transfected with pCMV-HA-POU6F1 were plated into a 10-cm dish and cellular extracts were immunoprecipitated with antibodies for HA (Abcam, ab9110) or IgG (Abcam, ab172730) coupled with protein A/G magnetic beads (MCE, HY-K0202) followed by Coomassie blue staining, western blotting, or MS analysis at Novogene Bioinformatics Technology Co., Ltd.

### Immunofluorescence (IF) staining

Cancer cells grown on 24-coverslips were transfected with POU6F1 overexpression or control plasmid. Then, cells were fixed with 4% paraformaldehyde for 15 min, permeabilized with 0.5% Triton X-100 for 20 min at room temperature, and blocked with 3% of bovine serum albumin for 30 min. Then, cells were incubated with antibodies specific for POU6F1 (abclonal, A7299) or RORA (Santa cruz biotechnology, sc-518081) at 4 °C overnight. Subsequently, the coverslips were incubated with Alexa Fluor 488 goat anti-mouse IgG (Abcam, ab150113) or cy3-goat anti-rabbit IgG (Abcam, ab150080), and stained with DAPI.

### Chromatin immunoprecipitation assay (ChIP)

ChIP assay was performed according to the instructions of the ChIP assay kit (Millipore, Bedford, MA, USA). HEK293T cells were plated into a 10-cm culture dish and transfected with the pCMV-HA-POU6F1 plasmid for 72 h. Cells were fixed with 1% formaldehyde for 10 min and then quenched with 125 mmol/L glycines. After being washed with cold PBS twice, the cells were collected and sonicated. Afterward, IP was conducted by using antibodies for HA (Abcam, ab9110), POU6F1 (abclonal, A7299), or IgG (Abcam, EPR25A). The co-precipitated DNAs were purified with phenol/chloroform for further PCR and analysis. Primer sets were listed in Supplementary Table 5.

### Dual-luciferase reporter assay

The human promoter regions of ENO1 (−2000/+100), PDK1 (−2000/+100), PRKCB (−2000/+300), RORA (−2000/−263), and HIF1A (−2000/−150) due to high GC-content were directly synthesized at TSINGKE (Beijing, China) and then inserted into the pGL3-Basic (Promega, Madison, WI, USA). Dual-luciferase assay was conducted following the manufacturer’s instructions (Promega). In brief, cancer cells were transfected with indicated expression plasmids, reporter plasmid, and Renilla luciferase at a ratio of 20:1 with lipofectamine 3000. Cells were lysed after 24 h of transfection and Firefly and Renilla luciferase activities were measured. Relative luciferase was calculated by normalizing Firefly activity to Renilla activity.

### Ubiquitination assay

HEK293T cells were transfected with Flag-ubiquitination (Flag-Ub) for 48 h and treated with 5 μmol/l MG132 (MCE, HY-13259) for 6 h. Then, cell lysates were subjected to IP with RORA antibody (MCE, HY-K0202) and RORA ubiquitination levels were determined using the anti-Flag antibody (Abcam, ab205606).

### In vivo growth and metastasis assays

All animal experiments were performed in accordance with NIH Guidelines for the Care and Use of Laboratory Animals and were approved by the Animal Care Committee of Tongji Medical College, Wuhan, China (IACUC number: 2541). The in vivo tumor growth and metastasis assays (*n* = 5 per group) were carried out in randomized male BALB/c nude mice (4 weeks old) purchased from National Rodent Seeds Center (Shanghai, China) as previously reported [24]. Stable transfection of POU6F1 or control A549 cells (5 × 10^6^ cancer cells per mouse) were injected into the right dorsal flanks or tail-vein of the mice, respectively. The tumor volume was monitored every other day by measuring the long axis (*a*) and the short axis (*b*) with a vernier caliper and tumor volumes were calculated as follows: volume = 1/2 (*a* × *b*^2^). In vivo growth assay, tumors were fixed, embedded in paraffin, and then subjected to immunohistochemical staining of proliferation index Ki-67 and CD31-positive intratumoral microvessels. Hematoxylin-Eosin was used to stain lungs from mice in the metastasis assay.

### In vivo imaging

In vivo fluorescence images of xenografts in nude mice were photographed under the In Vivo Optical Imaging System (In Vivo FX PRO, Bruker Corporation). The mice imaging was excited at 570 nm and emission was collected with a 600 nm filter. And the total exposure time was set at 10 s.

### Statistical analysis

All data were presented as a mean ± standard error of the mean (SEM). Student’s *t*-tests and analysis of variance (ANOVA) were performed to assess the difference between two groups and multiple groups, respectively. Log-rank test, univariate, and multivariate Cox regression models were used to evaluate survival differences and hazard ratios of POU6F1 using the “forest plot” package of R software. All statistical tests were two-sided and a *P-*value < 0.05 was considered statistically significant wherein *P < 0.05, **P < 0.01, ***P < 0.001.

## Results

### POU6F1 is downregulated and predicts poor prognosis in LUAD

**Fig. 1:**
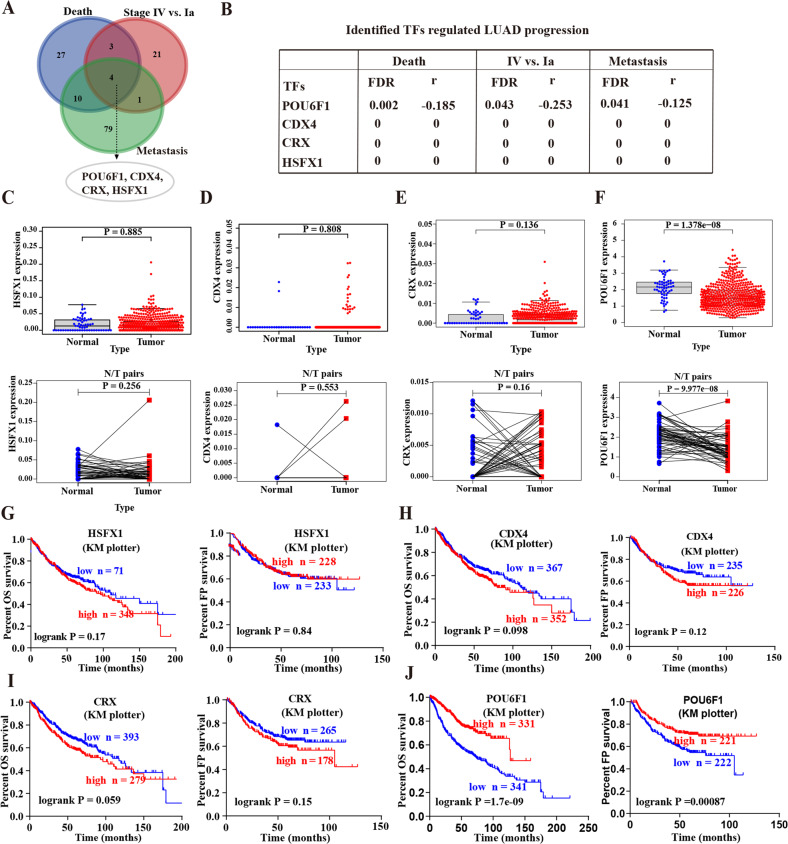
POU6F1 is identified as the potential transcription factor for regulating LUAD progression. **A**, **B** The potential transcription factors differentially expressed (*P* < 0.05) in 515 LUAD cases with the various status of death, clinical stage, or metastasis. **C**–**F** Scatter plot showing expression of HSFX1 (**C**), CDX4 (**D**), CRX (**E**), and POU6F1 (**F**) in unpaired (upper panel) or paired LUAD tissues (lower panel) compared with normal tissues. **G**–**J** Kaplan–Meier curves indicating overall survival (OS) and first progression (FP) of LUAD patients with high or low levels of HSFX1 (cutoff values = 25.00 and 24.00), CDX4 (cutoff values = 10.00 and 11.00), CRX (cutoff values = 5.00 and 5.00), and POU6F1 (cutoff values = 82.00 and 98.00) derived from Kaplan–Meier Plotter (http://kmplot.com/analysis).

To screen the essential TFs for regulating LUAD progression, we performed a comprehensive analysis of a public LUAD dataset in R2: Genomics Analysis and Visualization Platform (amc.nl) including 515 LUAD patients and identified 105, 29, and 94 significantly expressed TFs that were associated with the death, tumor stage, and metastasis, respectively (Fig. 1A and Supplementary Tables  1–3). Overlapping analyses eventually identified four potential TFs, including pou domain, class 6, transcription factor 1 (POU6F1), heat shock transcription factor family, x-linked 1 (HSFX1), caudal type homeobox 4 (CDX4), and cone-rod homeobox (CRX) (Fig. 1A, B). Mining of a public dataset (TCGA-LUAD) containing 535 LUAD cases and 59 normal cases revealed that there was no significant difference in the expression of HSFX1, CDX4, and CRX between LUAD tissues and normal tissues (*P* > 0.05, Fig. 1C–E). However, POU6F1 was significantly downregulated within LUAD tissues (*P* < 0.001, Fig. 1F). Kaplan–Meier analysis indicated that no significant survival difference in the overall survival (OS) and first progression (FP) was observed between the high expression and low expression groups of HSFX1, CDX4, and CRX (Fig. 1G–I). Conversely, the patients with low expression of POU6F1 had a significantly poor prognosis (*P* < 0.001, Fig. 1J). Based on the above analyses, we selected POU6F1 as the potential TF for further study.

**Fig. 2:**
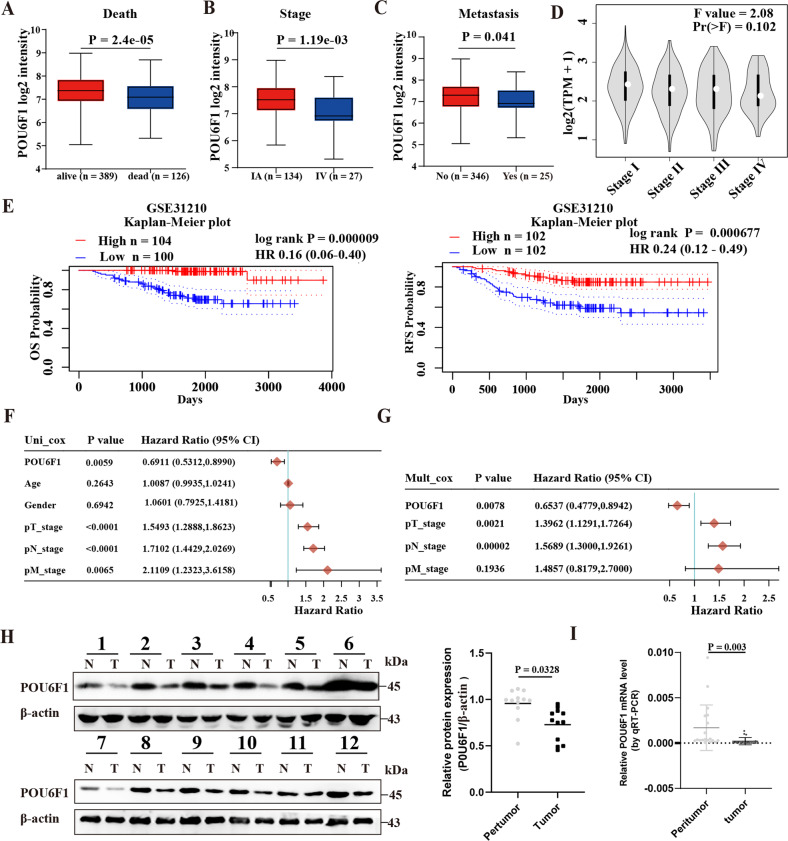
POU6F1 is downregulated and predicts poor prognosis in LUAD patients. **A**–**C** Mining the public dataset (LUAD-TCGA) showing the differential expression of POU6F1 in LUAD patients with different status of death (**A**), stage (**B**), and metastasis (**C**). **D** Homogeneity of variance analysis revealing the trend of POU6F1 expression in different tumor stages of LUAD patients. **E** Kaplan–Meier survival curve indicating overall survival (OS) and relapse-free survival (RFS) of LUAD patients with high or low levels of POU6F1 in the GEO dataset (GEO31210). **F**, **G** Pooled hazard ratios (HRs) based on univariate analysis (**F**) and multivariate analysis (**G**) for assessing the prognostic value of POU6F1 in LUAD patients. **H** Western blotting assay showing the expression of POU6F1 in human LUAD tissues and adjacent normal tissues (*n* = 12). Relative expression (**H** right panel) of POU6F1 was normalized to that of β-actin using ImageJ software. **I** Real-time qRT-PCR assay showing the expression of POU6F1 in LUAD tissues and adjacent normal tissues (*n* = 19).

Notably, POU6F1 was lowly expressed in LUAD tissues with death (*P* = 2.4 × 10^−5^), progression (*P* = 1.19 × 10^−3^), and metastasis (*P* = 0.041) (Fig. 2A–C). Additionally, POU6F1 expression decreased significantly with the increase of LUAD stage and the F value was 2.08 using Gene Expression Profiling Interactive Analysis (GEPIA) website (Fig. 2D and Supplementary Fig. 1). Moreover, survival analysis of POU6F1 was also validated using the GEO database: GEO31210 (Fig. 2E). We found that the expression of POU6F1 in LUAD was closely associated with clinical and pathological features, including gender, tumor stage, and N stage (Supplementary Table 6). POU6F1 was an independent protective prognostic factor in LUAD with hazard ratios less than 1 (Fig. 2F, G). Western blotting as well as real-time qRT-PCR showed the downregulation of POU6F1 in LUAD tissues (Fig. 2H, I). In summary, those results indicated that POU6F1 was significantly underregulated in LUAD tissues and maybe suppressive.

### Pan-cancer analysis of POU6F1

To further explore the roles of POU6F1 in various carcinogenesis, we investigated the POU6F1 expression level in multiple human cancers using Oncomine and TIMER. Our research revealed that the POU6F1 level was downregulated in various cancers, such as bladder urothelial carcinoma and lung squamous cell carcinoma, etc. (Supplementary Fig. 2A, B). On the contrary, the expression level of POU6F1 was markedly upregulated in cholangiocarcinoma and liver hepatocellular carcinoma. These results indicated that POU6F1 has a dual role in the carcinogenesis of cancers.

### POU6F1 represses the growth and aggressiveness of LUAD

**Fig. 3:**
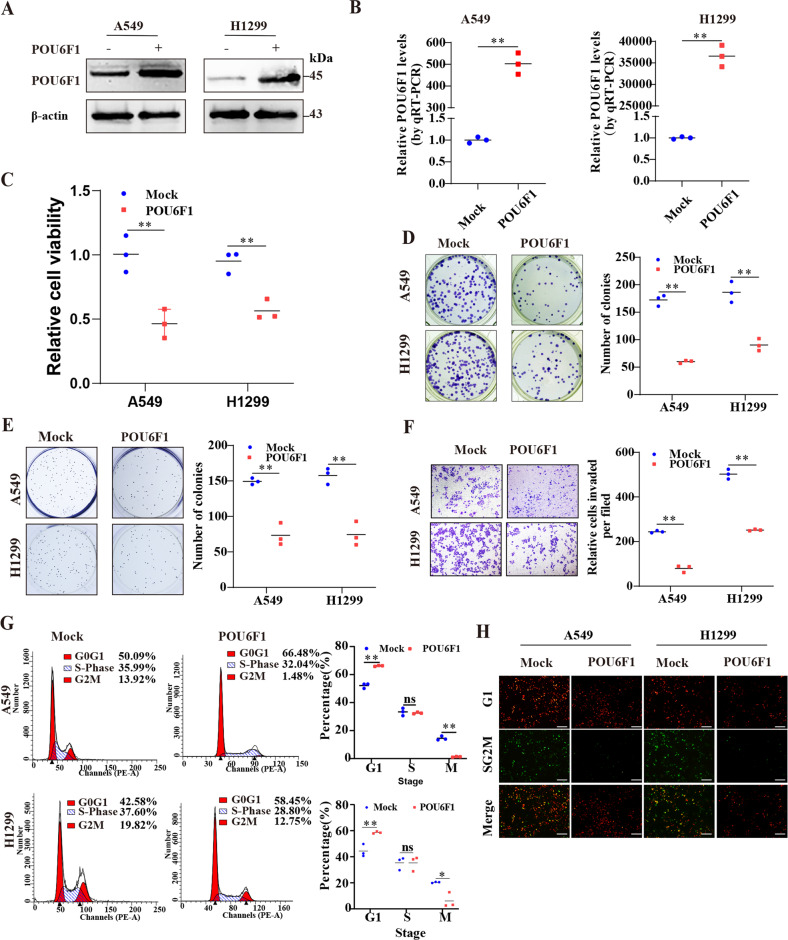
POU6F1 represses the growth and aggressiveness of LUAD cells in vitro. **A**, **B** Western blotting and real-time qRT-PCR assays revealing the POU6F1 expression in A549 and NCI-H1299 cells stably transfected with empty vector (mock) or POU6F1. **C**, **D** CCK8 and colony formation assays indicating cell viability of A549 and NCI-H1299 cells stably transfected with mock or POU6F1. **E**, **F** Representative images (left panel) and quantification (right panel) of soft agar and transwell assays revealing the migration ability of A549 and NCI-H1299 cells stably transfected with mock or POU6F1. **G** Cell cycle detection in A549 and NCI-H1299 cells transfected with empty vector (pCMV-HA) or pCMV-HA-POU6F1. **H** Immunofluorescence revealing cycle change of A549 and NCI-H1299 cells transfected with pCMV-HA or pCMV-HA-POU6F1, and those co-transfected with pRetroX-G1-Red and pRetroX-SG2M-Cyan vector. Scale bar: 100 μm. The student’s *t*-test compared the difference in **B**–**G**. *P < 0.05, **P < 0.01.

**Fig. 4:**
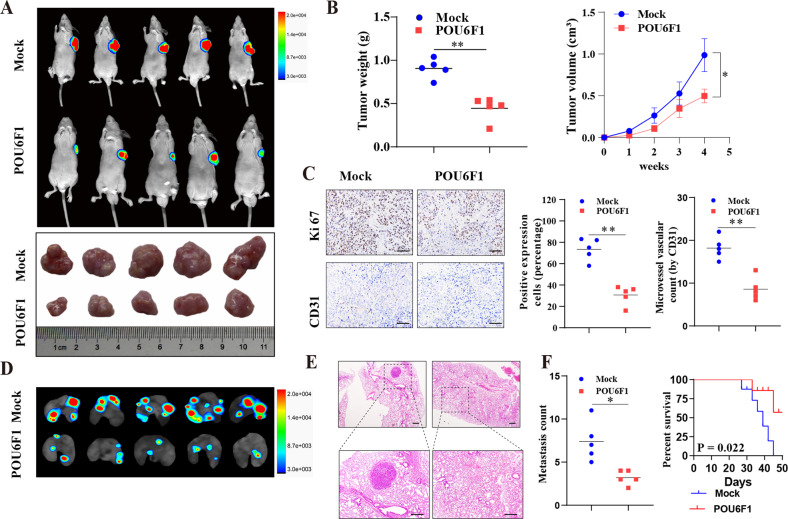
POU6F1 represses the growth and aggressiveness of LUAD cells in vivo. **A** Representative images of xenografts formed by subcutaneous injection of A549 cells stably transfected with empty vector (mock) or POU6F1 into the dorsal flanks of nude mice (*n* = 5 for each group). **B** Tumor weight at the endpoints (**B** left panel) and in vivo growth curve (**B** right panel) of xenografts formed by subcutaneous injection of A549 cells stably transfected with mock or POU6F1 into the dorsal flanks of nude mice (*n* = 5 for each group). **C** Representative images (left panel) and quantification (right panel) of immunohistochemical staining revealing the expression of Ki-67 and CD31 within xenografts formed by subcutaneous injection of A549 cells stably transfected with mock or POU6F1 into dorsal flanks of nude mice. Scale bar: 100 μm. **D**–**F** Representative images (**D**), HE staining (**E**), metastatic counts of lungs (**F** left panel), and Kaplan–Meier curves (**F** right panel) of nude mice treated with tail vein injection of A549 cells stably transfected with mock or POU6F1 (*n* = 5 for each group). Scale bar: 100 μm. *P < 0.05, **P < 0.01.

We further explored the biological behaviors of POU6F1 in LUAD cells. Stable LUAD cell lines transfected with POU6F1 overexpression or control vectors were constructed after selection with puromycin (Fig. 3A, B). Meanwhile, based on clustered regularly interspaced short palindromic repeats (CRISPR) interference (CRISPRi), we constructed two independent gRNAs in A549 and NCI-H1299 cell lines against POU6F1 (CRISPRi-POU6F1) (Supplementary Fig. 3A, B). CCK8 and colony formation assays revealed that overexpressing POU6F1 decreased the viability of A549 and NCI-H1299 cells (Fig. 3C, D). In soft agar, transwell, and scratch assays, forced expression of POU6F1 reduced the growth and invasion of A549 and NCI-H1299 cells (Fig. 3E, F and Supplementary Fig. 3C). Moreover, upregulation of POU6F1 resulted in an increased percentage of cells in the G0/G1 phase and a decreased percentage of cells in the G2/M phase (Fig. 3G, H). In addition, ectopic expression of POU6F1 led to a marked reduction in the growth and tumor weight of subcutaneous xenograft tumors (Fig. 4A, B). The intensity of proliferation index Ki-67 and CD31-positive intratumoral microvessels in stable overexpressing POU6F1 tumors was decreased as determined by immunohistochemical staining of xenografts (Fig. 4C). In experimental metastasis assay, fewer lung metastatic counts and a higher survival possibility of nude mice via the tail vein were noted in the overexpression of POU6F1 A549 cells (Fig. 4D–F). Collectively, these results suggested the suppressive roles of POU6F1 in tumorigenesis and aggressiveness of LUAD.

### POU6F1 inactivates HIF1A signaling pathway and transcriptionally regulates ENO1, PDK1, and PRKCB expression

**Fig. 5:**
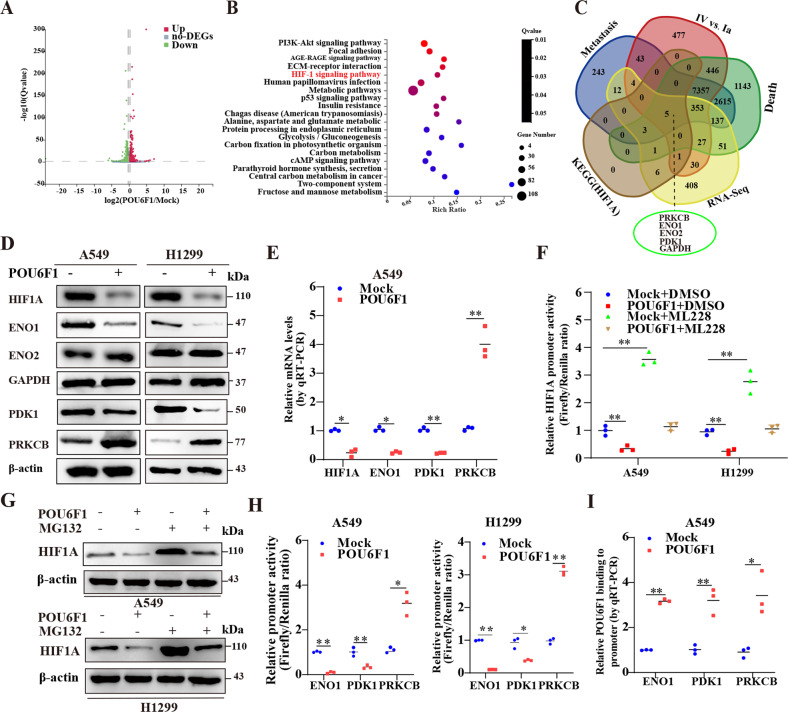
POU6F1 inactivates HIF1A signaling pathway and transcriptionally regulates ENO1, PDK1, and PRKCB expression. **A** Volcano plot showing differential expression genes (DEGs) in A549 cells stably transfected with POU6F1 compared with empty vector (mock). **B** Human KEGG pathway analysis of DEGs indicating participated pathways in A549 cells stably transfected with POU6F1 relative to mock. **C** Venn diagram revealing comprehensive analysis of genes, that were involved with the HIF1A signaling pathway and associated with LUAD progression in various status of death, tumor stage, and metastasis. **D** Western blotting assay showing the expression of HIF1A, ENO1, ENO2, GAPDH, PDK1, and PRKCB in A549 and NCI-H1299 cells stably transfected with mock or POU6F1. **E** Real-time qRT-PCR assay indicating the expression of HIF1A, ENO1, PDK1, and PRKCB in A549 cells stably transfected with mock or POU6F1. **F** Dual-luciferase assay indicating relative activity of HIF1A promoter in A549 and NCI-H1299 cells stably transfected with mock or POU6F1, and those treated with DMSO or ML228 (1.0 μmol/l). **G** Western blotting assay showing the expression of HIF1A in A549 and NCI-H1299 cells stably transfected with mock or POU6F1, and those treated with DMSO or MG132 (5 μmol/l) for 6 h. **H** Dual-luciferase assay showing relative activity of ENO1, PDK1, and PRKCB promoter in A549 and NCI-H1299 cells transfected with mock or POU6F1. **I** ChIP and qPCR assays indicating relative POU6F1 enrichment on ENO1, PDK1, and PRKCB promoter in A549 cells stably transfected with mock or POU6F1. Student *t*-test and ANOVA compared the difference in **E**, **F** and **H**, **I**. *P < 0.05, **P < 0.01.

To reveal the mechanism via which POU6F1 suppressed tumorigenesis, we performed RNA‑seq assay to screen differential expressed genes (DEGs), which revealed 1269 upregulated and 1467 downregulated genes with the condition false discovery rate less than 0.05 in A549 cells upon POU6F1 overexpression (Fig. 5A). Kyoto Encyclopedia of Genes and Genomes (KEGG) pathway category and Gene Ontology (GO) analysis indicated that DEGs were involved in various cellular biological processes, such as cell growth and death, cell adhesion, etc. (Supplementary Fig. 4A, B). The DEGs were closely related to negative regulation of cell proliferation and positive regulation of the apoptotic process, which further validated the inhibitory effect of POU6F1 (Supplementary Fig. 4B). What is more, KEGG functional enrichment analysis revealed that POU6F1 participated in several growth- and invasion-related pathways, such as HIF-1 signaling pathway and PI3K-AKT signaling pathway (Fig. 5B). We made an overlapping analysis and identified five differentially expressed genes that were involved with HIF1A signaling pathway and associated with LUAD progression, including enolase 1 (ENO1), enolase 1 (ENO2), pyruvate dehydrogenase kinase 1 (PDK1), protein kinase C beta (PRKCB), and GAPDH (Fig. 5C). It has been reported that these genes were closely associated with cancer progression, including ENO1 [25, 26], PRKCB [27], ENO2 [28, 29], and PDK1 [30].

Next, we probed the effect of POU6F1 on the expression of HIF1A and five target genes. HIF1A, ENO1, and PDK1 expression were downregulated and PRKCB was upregulated in A549 and NCI-H1299 cells with overexpression of POU6F1, but not of GAPDH and ENO2 as indicated by western blotting and real-time qRT-PCR (Fig. 5D, E and Supplementary Fig. 5A–C). Dual-luciferase assay revealed that overexpressing POU6F1 decreased HIF1A activity and abolished the effects of HIF1A activator 1.0 μmol/l ML228 [31] (Fig. 5F). Furthermore, we determined whether POU6F1 regulated HIF1A expression by ubiquitination and found that the decreased HIF1A expression induced by overexpression of POU6F1 was abolished by treatment with a proteasome inhibitor (MG132) (Fig. 5G), suggesting that POU6F1 increased HIF1A ubiquitination and degradation.

Previous studies have revealed that ENO1 and PDK1 were target genes of HIF1A [32, 33]. We wondered whether POU6F1 could directly bind to three genes promoter regions. Dual-luciferase reporter assay exhibited that forced POU6F1 expression decreased ENO1 and PDK1 activity and enhanced PRKCB activity in A549 and NCI-H1299 cells (Fig. 5H). But, HEK293T cells displayed dropped PDK1 activity and increased ENO1 and PRKCB activity (Supplementary Fig. 5D). The ChIP and real-time qRT-PCR assays revealed that overexpression of POU6F1 enhanced the enrichment of POU6F1 on the promoter regions of ENO1, PDK1, and PRKCB in A549 cells (Fig. 5I). Collectively, POU6F1 could inhibit the transcriptional activity of HIF1A and directly bind to the promoter regions of three genes to affect their expression.

### POU6F1 physically interacts with RORA in LUAD cells

**Fig. 6:**
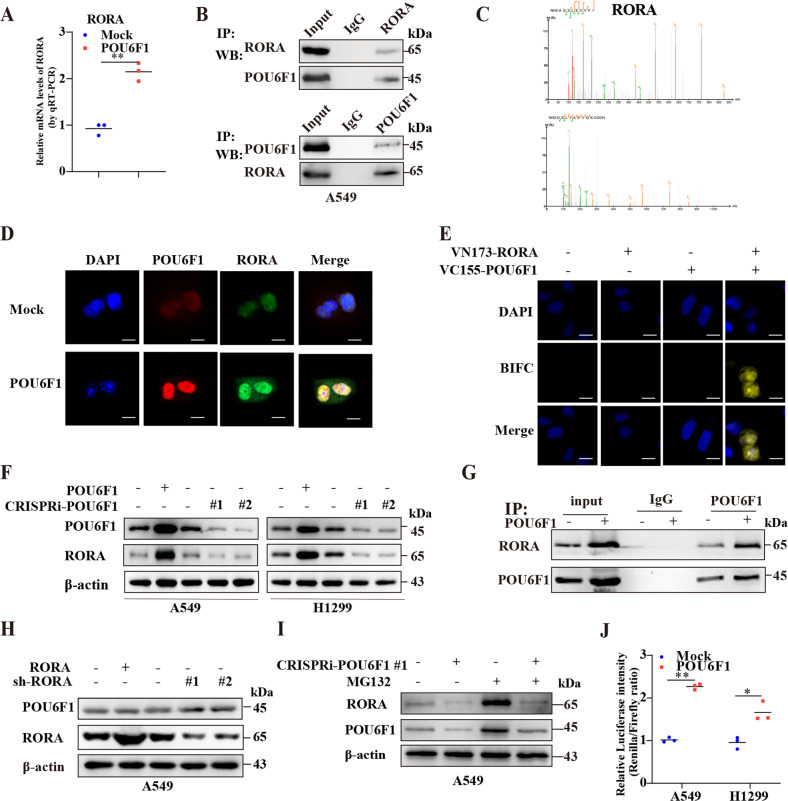
POU6F1 binds RORA and enhances the stabilization and activity of RORA. **A** Real-time qRT-PCR assay indicating the expression of RORA in A549 cells stably transfected with empty vector (mock) or POU6F1. **B** Co-IP and western blotting assays indicating the endogenous interaction between POU6F1 and RORA in A549 cells. The immunoglobulin G (IgG)-bound protein was taken as the negative control. **C** Mass spectrometry detection showing the spectrogram of RORA in A549 and HEK293T cells pulled down by POU6F1 antibody. **D** Immunofluorescence assay confirming the colocalization of POU6F1 and RORA in A549 cells transfected with empty vector (pCMV-HA) or pCMV-HA-POU6F1. Scale bars:10 µm. **E** Confocal images of BiFC showing the direct visualization of POU6F1 and RORA in A549 cells co-transfected with pBiFC-VC155-POU6F1 and pBiFC-VN173-RORA. Scale bars:10 µm. **F** Western blotting assay showing the expression of RORA in A549 and NCI-H1299 cells transfected with mock, POU6F1, CRISPRi-Scb, CRISPRi-POU6F1 #1, or CRISPRi-POU6F1 #1. **G** Co-IP and western blotting assays indicating the interaction between POU6F1 and RORA in A549 cells transfected with mock or POU6F1. **H** Western blotting assay showing the expression of POU6F1 and RORA in A549 cells transfected with mock, RORA, scramble shRNA (sh-Scb), sh-RORA #1, or sh-RORA #2. **I** Western blotting assay showing the expression of RORA in A549 cells transfected with CRISPRi-Scb or CRISPRi-POU6F1 #1, and those treated with MG132 (5 μmol/l) for 6 h. **J** Dual-luciferase assay indicating relative promoter activity of RORA in A549 and NCI-H1299 cells transfected with mock or POU6F1. Student *t*-test compared the difference in **A** and **J**. *P < 0.05, **P < 0.01.

IF and western blotting revealed that the POU6F1 was mainly localized to the nucleus in A549 and NCI-H1299 cells (Supplementary Fig. 6A, B). Interestingly, RNA‑seq showed that the expression of RORA was increased in POU6F1 overexpression A549 cells (Fig. 6A). It has been reported that RORA regulated transcriptional activity of HIF1A and participated in the HIF1A signaling pathway [19, 20]. Meanwhile, RORA has been extensively studied in various cancers [14, 15, 34]. Therefore, we explored whether an interaction existed between POU6F1 and RORA. The endogenous interaction between POU6F1 and RORA was performed via IP followed by western blotting in A549 cells (Fig. 6B). Furthermore, exogenous IP and MS further validated the interaction between POU6F1 and RORA in A549 and HEK293T cells (Fig. 6C and Supplementary Table 7). IF confirmed the colocalization of POU6F1 and RORA in the nucleus of A549 cells (Fig. 6D). BiFC assay was further performed for direct visualization of their interaction (Fig. 6E). These findings collectively suggested the physical interaction between POU6F1 and RORA.

### POU6F1 influences the stabilization and activity of RORA

Notably, overexpression or knockdown of POU6F1 increased and decreased protein level of RORA, respectively (Fig. 6F). Moreover, the Co-IP and western blotting indicated that overexpressing POU6F1 promoted the interaction between POU6F1 and RORA in A549 cells (Fig. 6G). Lower expression of RORA was observed in LUAD cell lines than that of HBE (Supplementary Fig. 6C). Overexpression of RORA led to its upregulation, while, knockdown of RORA by two independent short hairpin RNAs resulted in a reduction of RORA (Supplementary Fig. 6D). But, ectopic expression or knockdown of RORA did not affect the expression of POU6F1 (Fig. 6H and Supplementary Fig. 6E, F). These results proved that POU6F1 regulated RORA expression. Then, we explored the mechanism by which POU6F1 regulated RORA expression. As expected, RORA displayed ubiquitylation (Supplementary Fig. 6G). We found that the decreased expression of RORA induced by knockdown of POU6F1 could be reversed by treatment with 5 μmol/l MG132, suggesting that POU6F1 stabilized RORA (Fig. 6I). Noteworthy, using the JASPAR database (https://jaspar.genereg.net/), we found POU6F1 binding sites in the RORA promoter region (Supplementary Table 8). Therefore, we constructed RORA promoter-luciferase reporter and dual-luciferase assay showed that ectopic expression of POU6F1 increased the activity of RORA promoter in A549 and NCI-H1299 cells (Fig. 6J).

POU6F1 bore a significant positive correlation with RORA (*R* = 0.77, *P* = 4.0 × 10^−163^) using GEPIA (Supplementary Fig. 6H). The relation was also validated in 515 LUAD cases from the TCGA database, revealing a relatively modest correlation (*R* = 0.39, *P* = 3.3 × 10^−20^), which probably resulted from LUAD histological heterogeneity (Supplementary Fig. 6I). Taken together, these results indicated that POU6F1 directly bound to the RORA promoter region and increased transcriptional activity of RORA in LUAD cells.

We then explored the biological role of RORA in LUAD. RORA expression was found to be significantly associated with death, tumor stage, and metastasis of LUAD (Supplementary Fig. 7A). The expression of RORA was downregulated in LUAD tissues and lower expression of RORA was associated with a poor survival probability of LUAD patients (Supplementary Fig. 7B, C). We detected RORA expression in our LUAD samples and the result was in line with the above findings (Supplementary Fig. 7D). Dual-luciferase assay showed that overexpression of RORA suppressed promoter activity of HIF1A, ENO1, or PDK1, and increased promoter activity of PRKCB in A549 cells (Supplementary Fig. 7E, F). In summary, these results indicated that RORA acted as a suppressor in LUAD and affected the activity of HIF1A and HIF1A pathway-associated genes.

### POU6F1 coordinates with RORA to inhibit cancer progression

**Fig. 7:**
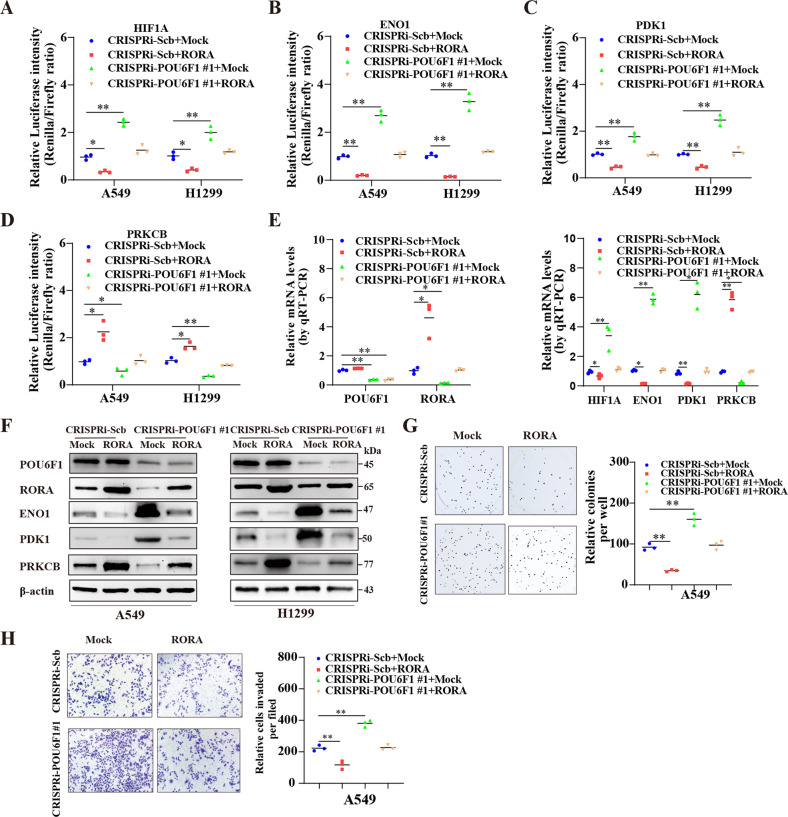
POU6F1 coordinates with RORA to inhibit LUAD progression. **A**–**D** Dual-luciferase assay indicating the relative promoter activity of HIF1A, ENO1, PDK1, and PRKCB in A549 and NCI-H1299 cells transfected with mock, RORA, CRISPRi-Scb, or CRISPRi-POU6F1 #1. **E**, **F** Real-time qRT-PCR and western blotting assays showing the expression of POU6F1, RORA, HIF1A, ENO1, PDK1, and PRKCB in A549 cells transfected with mock, RORA, CRISPRi-Scb, or CRISPRi-POU6F1 #1. **G**, **H** Representative images (left panel) and quantification (right panel) of soft-agar (**G**) and transwell (**H**) assays indicating the growth and invasion of A549 cells transfected with mock, RORA, CRISPRi-Scb, or CRISPRi-POU6F1 #1. Student’s *t*-test and ANOVA compared the difference in **A**–**E** and **G**–**H**. *P < 0.05, **P < 0.01.

We further examined the functional interplay between POU6F1 and RORA in the regulation of LUAD progression. In the dual-luciferase assay, overexpression of RORA attenuated the increased HIF1A activity elicited by silencing of POU6F1 (Fig. 7A). Knockdown of POU6F1 increased the promoter activity of ENO1 and PDK1 and decreased PRKCB activity, which was prevented by ectopic expression of RORA in A549 and NCI-H1299 cells (Fig. 7B–D). Furthermore, forced expression of RORA suppressed the increased expression of HIF1A, ENO1, or PDK1 and reduction of PRKCB in LUAD cells induced by impaired POU6F1 expression (Fig. 7E, F and Supplementary Fig. 8A, B). Knockdown of POU6F1 caused an increase in the growth and invasiveness, which were decreased by forced expression of RORA in A549 and NCI-H1299 cells (Fig. 7G, H and Supplementary Fig. 8C, D). Mining public dataset indicated that ENO1 and PDK1 expression were highly expressed in LUAD tissues and patients with higher ENO1 and PDK1 expression were linked with poor outcomes (Supplementary Fig. 9A, B). At the same time, PRKCB was reduced in tumor tissues and downregulated PRKCB expression was indicative of a low survival possibility in LUAD patients (Supplementary Fig. 9C). In consistence with this, the expression of ENO1 and PDK1 were elevated and PRKCB was decreased in matched LUAD tumor tissues compared with normal tissues (Supplementary Fig. 9D). Collectively, these results revealed that POU6F1 cooperated with RORA to exert the inhibitory effect in LUAD cells.

## Discussion

Transcriptional regulation is one of the fundamental molecular processes taking place in cells [35]. Aberrant transcription, a hallmark of practically all kinds of cancers, is responsible for the growth, proliferation, and invasiveness of cells [36, 37]. In the present study, we identified POU6F1 as a transcription factor, which was closely associated with tumor stage and death by mining a public dataset. We revealed that POU6F1 was downregulated in LUAD and decreased expression predicted unfavorable outcomes in LUAD.

In the study, we determined that upregulation of POU6F1 suppressed tumor proliferation and aggressiveness in vitro and in vivo. Of note, upregulated POU6F1 decreased 24 h LUAD cell viability and decreased cell migration ability resulting from overexpressing POU6F1 might be ascribed directly to the impaired cell migration or indirectly to the decreased cell growth ability. Moreover, cell cycle revealed that POU6F1 reduced G2/M phase population and there existed a significant difference in the G2/M phase of cell cycle in A549 and NCI-H1299 cells induced by POU6F1 overexpression. It might be ascribed to the properties of A549 (p53-positive) and NCI-H1299 cells (p53-null) and further studies were needed to address this issue [38, 39]. Suzuki et al. showed that POU6F1 promoted the proliferation of clear cell adenocarcinoma cell lines [10]. The results suggested that POU6F1 played promoting or suppressive roles in different cancers in a context-specific way.

The mechanism research revealed that POU6F1 was involved in the HIF1A signaling pathway. We showed that POU6F1 suppressed HIF1A transcriptional activity. It has been previously reported that HIF1A plays an essential role in cellular proliferation and angiogenesis by regulating the transcription of diverse genes, such as VEGF and PD-L1 [40, 41]. HIF1A stabilization can be regulated by a wide array of regulators [42, 43]. For example, the small ubiquitin-like modifier-1 was co-immunoprecipitated with HIF1A, increased HIF1A stability, and enhanced its transcriptional activity [44]. In this study, we found that POU6F1 promoted HIF1A ubiquitination and degradation in LUAD cells. HIF1A directly transactivates various genes, such as PDK1 [33]. In this study, we demonstrated that POU6F1 enriched on the promoter of genes ENO1, PDK1, and PRKCB, and affected the luciferase activities of three genes. But ENO1 activity in LUAD cells and HEK293T cells displayed a contrary trend, which was interesting and needed further studies to gain sight into the cause. The three genes were correlated with cancer proliferation. For example, ENO1 is elevated in various tissue and involved in the oncogenesis of human cancers [45]. ENO1 promotes cellular glycolysis, growth, invasion, and metastasis in NSCLC through the FAK-mediated PI3K/AKT pathway [46]. PDK1 plays a vital role in the oncogenesis and progression of human cancers and contributes to poor prognosis [30, 47].

As a transcriptional regulator, POU6F1 has been reported to enhance PRL gene expression directly through CBP and indirectly by the activation of Pit-1 gene expression [48]. In the current study, based on the Co-IP, BiFC, and MS assays, we revealed that POU6F1 could interact with RORA in LUAD cells. In addition, POU6F1 could enhance transcriptional activity and stabilization of RORA. Moreover, we constructed RORA luciferase reporters with approximately 2 kb promoter sequences and verified that overexpressing POU6F1 enhanced the promoter activity of RORA. However, we did not conduct further studies to determine the exact binding sequence between POU6F1 and RORA.

We identified that RORA expression was significantly downregulated in LUAD tissues, which was further confirmed in LUAD cell lines compared with HBE. RORA could act as a repressor in LUAD and the finding was consistent with previous studies on cancers, such as prostate cancer and hepatocellular carcinoma [49, 50]. In the study, dual-luciferase assay showed that RORA suppressed the transactivation of HIF1A in LUAD cells. However, Lee et al. suggested that RORA mediated the activation of HIF1A under hypoxic conditions [19]. The discrepancy may be due to varied models and different vectors used. Of note, RORA influenced the transcript alternation of target genes and cancer growth induced by the knockdown of POU6F1, indicating the suppressive role of POU6F1/RORA in the progression of LUAD.

There are some limitations in the study. First, our research mainly studied the inhibitory effect and working mechanism of POU6F1 in LUAD cells. Therefore, whether the function and mechanisms could be extrapolated to other types of cancers warrants further investigation. Due to word limit, we could not include more cancers in this study. Second, our study principally examined its effect on the widely studied HIF1A signaling pathway and we could not cover other cell signaling pathways in LUAD because of space limitation.

## Conclusion

In summary, we identified that POU6F1 was markedly downregulated in LUAD tissues and downregulation of POU6F1 was associated with the unfavorable outcome of LUAD patients. In vitro and in vivo assays revealed that POU6F1 suppressed cell growth and migration of LUAD cells. Mechanistically, POU6F1 bound and stabilized RORA to inhibit the transcriptional activity of HIF1A and alter the expression of HIF1A signaling-associated genes, thereby leading to the suppression of LUAD cells. POU6F1 analog peptides may be developed based on the functional domains of POU6F1 for the treatment of LUAD in the further. And targeting the HIF1A signaling pathway may be a therapeutic strategy for the inhibition of LUAD with common HIF1A inhibitors.

## Supplementary information

Reproducibility Checklist

Supplementary Material

Supplementary Figure and Table legends

POU6F1 WB

Supplementary Figure 1

Supplementary Figure 2

Supplementary Figure 3

Supplementary Figure 4

Supplementary Figure 5

Supplementary Figure 6

Supplementary Figure 7

Supplementary Figure 8

Supplementary Figure 9

Supplementary Table 1

Supplementary Table 2

Supplementary Table 3

Supplementary Table 4

Supplementary Table 5

Supplementary Table 6

Supplementary Table 7

Supplementary Table 8

## Data Availability

All the data supporting the conclusions of this article are presented within the article and its Supplementary materials.
