# Supplementary material for: POU6F1 cooperates with RORA to suppress the proliferation of lung adenocarcinoma by downregulating HIF1A signaling pathway
Source: Cell Death Dis. 2022 May 3;13(5):427. doi: 10.1038/s41419-022-04857-y (PMC9065044; doi:10.1038/s41419-022-04857-y)
Supplement: Supplementary file 2 — Supplementary Material [file 41419_2022_4857_MOESM2_ESM.docx]

**Supplementary information**

**Supplementary Figures**


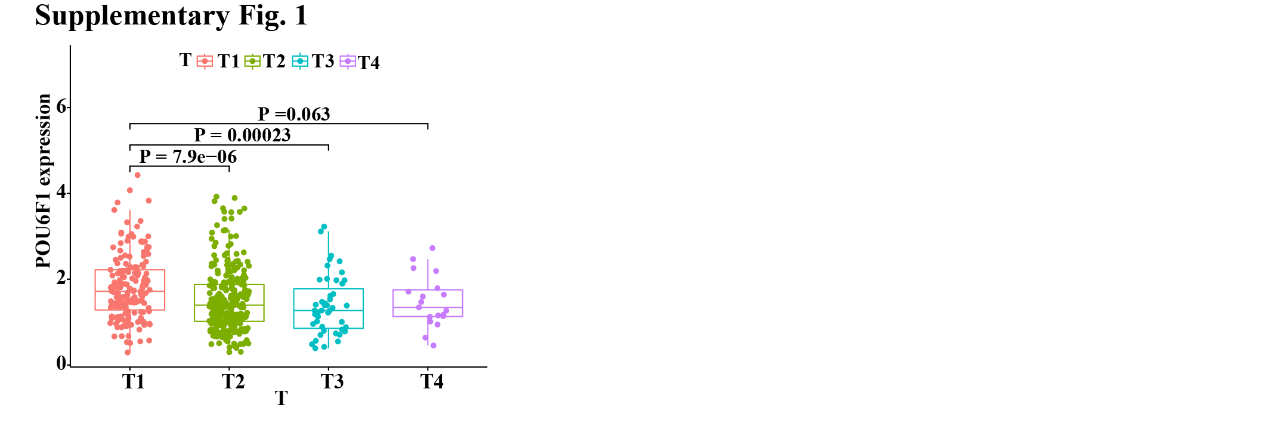


Homogeneity of variance analysis revealing the trend of POU6F1 expression level in the T stage of LUAD patients.

**
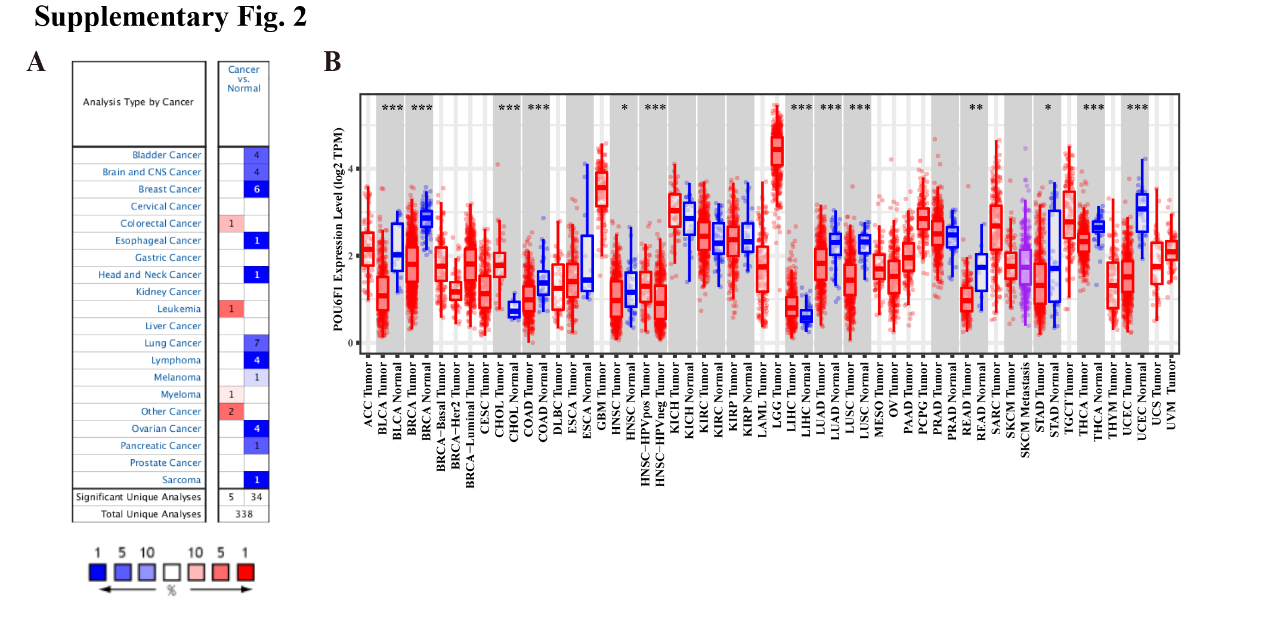
A.** Oncomine database indicating the pan-cancer summary of POU6F1. The threshold *P*-value is limited to less than 0.001 and the fold change is 1.5. **B.** TIMER database showing the differential expression level of POU6F1 between tumor and adjacent normal tissues in all TCGA tumors. ACC, adrenocortical carcinoma; BLCA, bladder urothelial carcinoma; BRCA, breast invasive carcinoma, CESC, cervical squamous cell carcinoma and endocervical adenocarcinoma; CHOL, cholangio carcinoma; COAD, colon adenocarcinoma; DLBC, lymphoid neoplasm diffused large B-cell Lymphoma; ESCA, esophageal carcinoma; GBM, glioblastoma multiforme; HNSC, head and neck squamous cell carcinoma; KICH, kidney chromophobe; KIRC, kidney renal clear cell carcinoma; KIRP, kidney renal papillary cell carcinoma; LAML, acute myeloid leukemia; LGG, lower grade glioma; LIHC, liver hepatocellular carcinoma; LUAD, lung adenocarcinoma; LUSC, lung squamous cell carcinoma; MESO, mesothelioma; OV, ovarian serous cystadenocarcinoma; PAAD, pancreatic adenocarcinoma; PCPG pheochromocytoma and paraganglioma; PRAD, prostate adenocarcinoma; READ, rectum adenocarcinoma; SARC, sarcoma; SKCM, skin cutaneous melanoma; STAD, stomach adenocarcinoma; TGCT, testicular germ cell tumors; THCA, thyroid carcinoma; THYM, thymoma; UCEC, uterine corpus endometrial carcinoma; UCS, uterine carcinosarcoma; UVM, uveal melanoma. ∗*P* < 0.05; ∗∗*P* < 0.01; ∗∗∗*P* < 0.001.

**
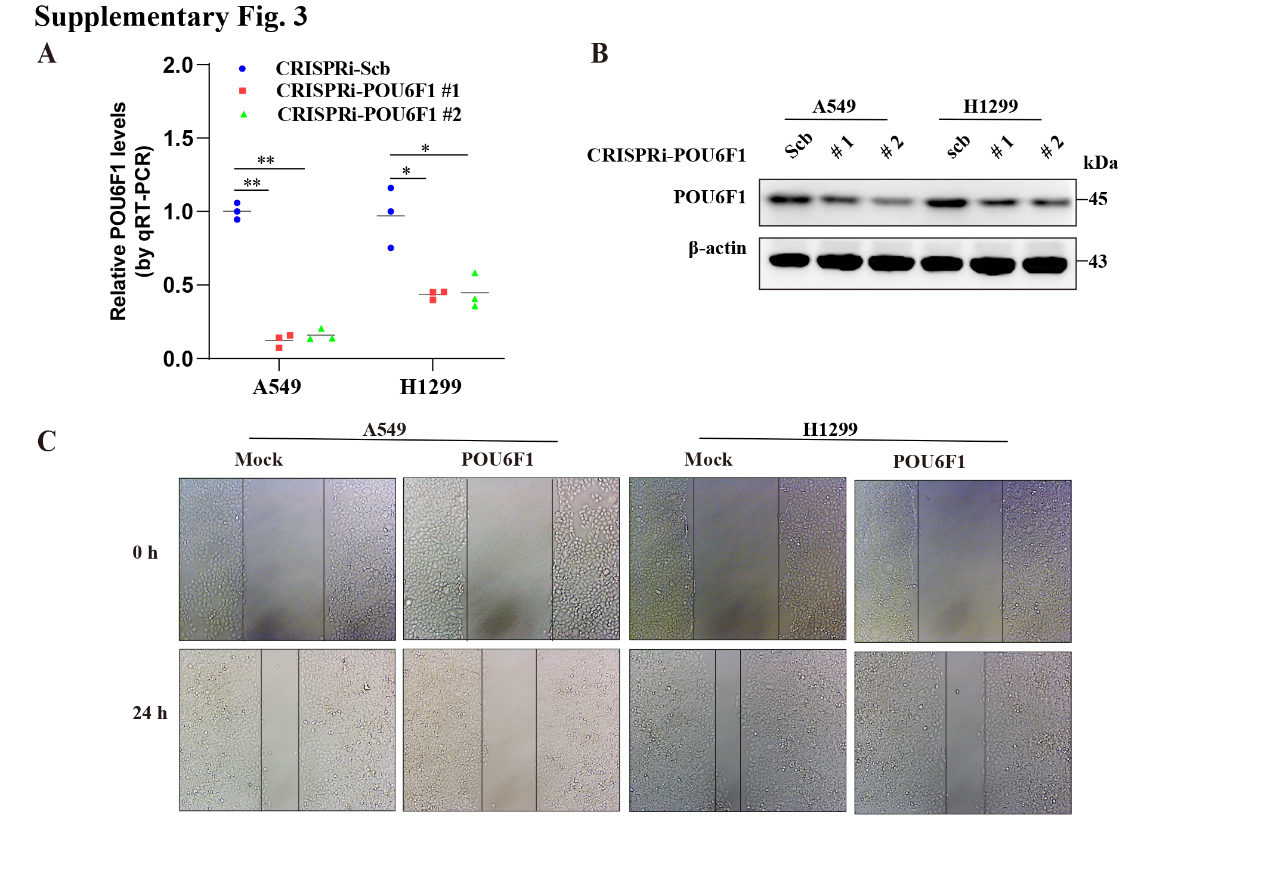
**

**A-B.** Real-time qRT-PCR and western blotting assays indicating the expression of POU6F1 in A549 and NCI-H1299 cells transfected with CRISPRi-Scb, CRISPRi-POU6F1 #1, or CRISPRi-POU6F1 #2. **C.** Wound-healing assay revealing the migration ability of A549 and NCI-H1299 cells stably transfected with empty vector (mock) or POU6F1. Student’s t-test compared the difference in A. **P* < 0.05, ***P* < 0.01.

**
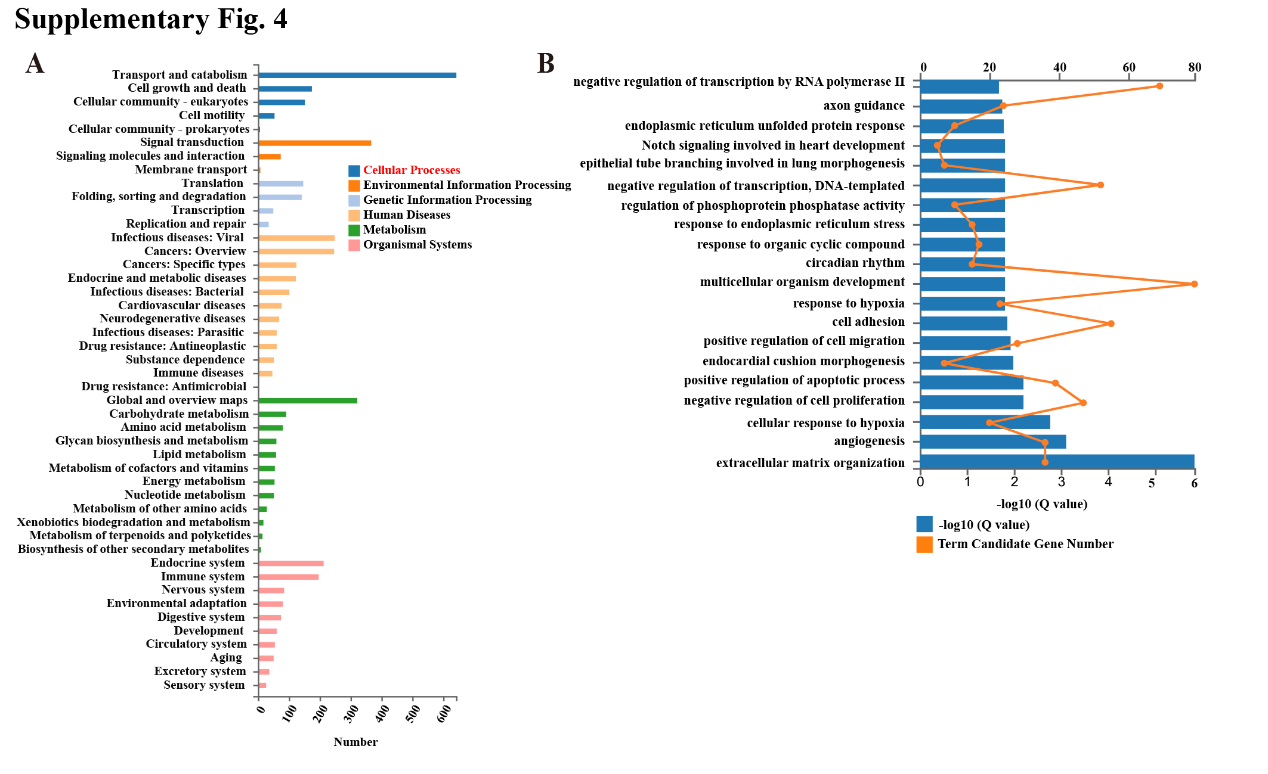
**

**A.** Kyoto Encyclopedia of Genes and Genomes (KEGG) classification analysis of differential expression genes (DEGs) in A549 cells stably transfected with POU6F1 compared with empty vector (mock). **B.** Gene Ontology (GO) analysis indicating the involved pathway of DEGs in A549 cells stably transfected with POU6F1 relative to mock.

**
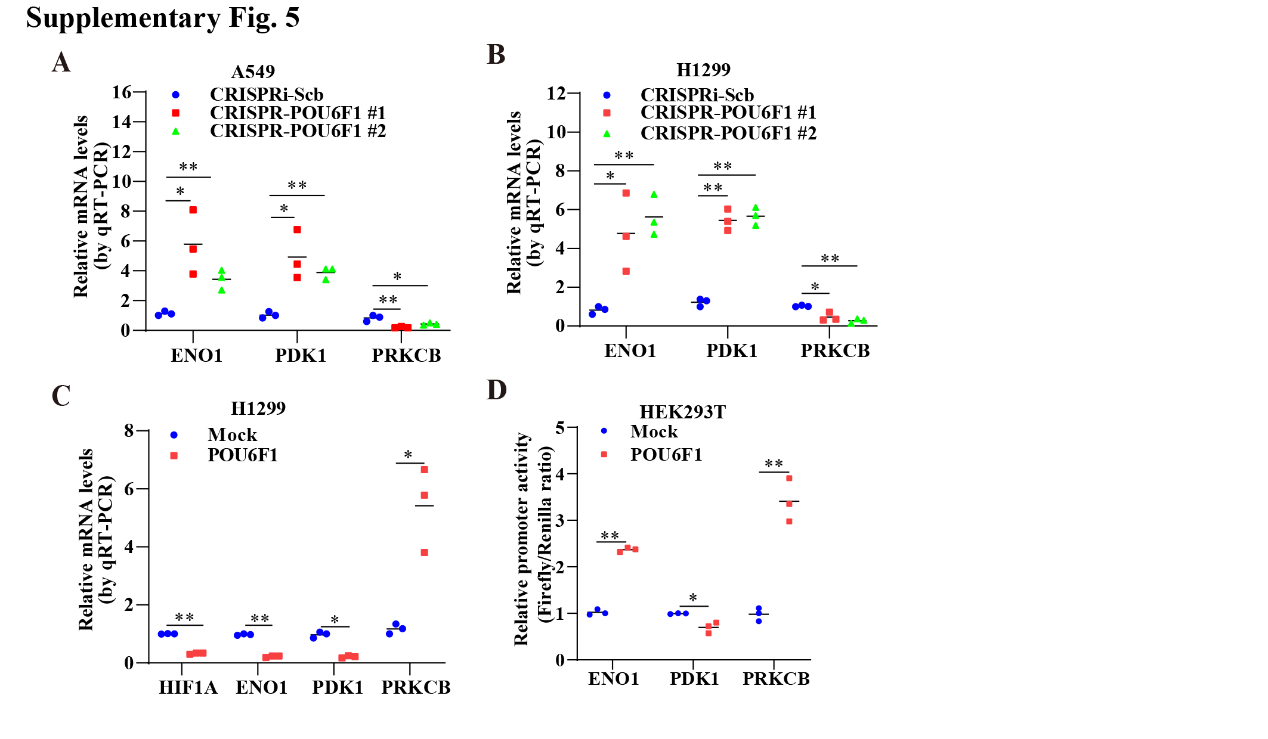
**

**A-B.** Real-time qRT-PCR assay indicating the expression of ENO1, PDK1, and PRKCB in A549 and NCI-H1299 cells transfected with CRISPRi-Scb, CRISPR-POU6F1 #1, or CRISPR-POU6F1 #2. **C.** Real-time qRT-PCR assay showing the expression of HIF1A, ENO1, PDK1, and PRKCB in NCI-H1299 cells transfected with empty vector (mock) or POU6F1. **D.** Dual-luciferase assay showing relative activity of ENO1, PDK1, and PRKCB promoter in HEK293T cells transfected with mock or POU6F1. Student’s t-test and ANOVA compared the difference in A–D. **P* < 0.05, ***P* < 0.01.

**
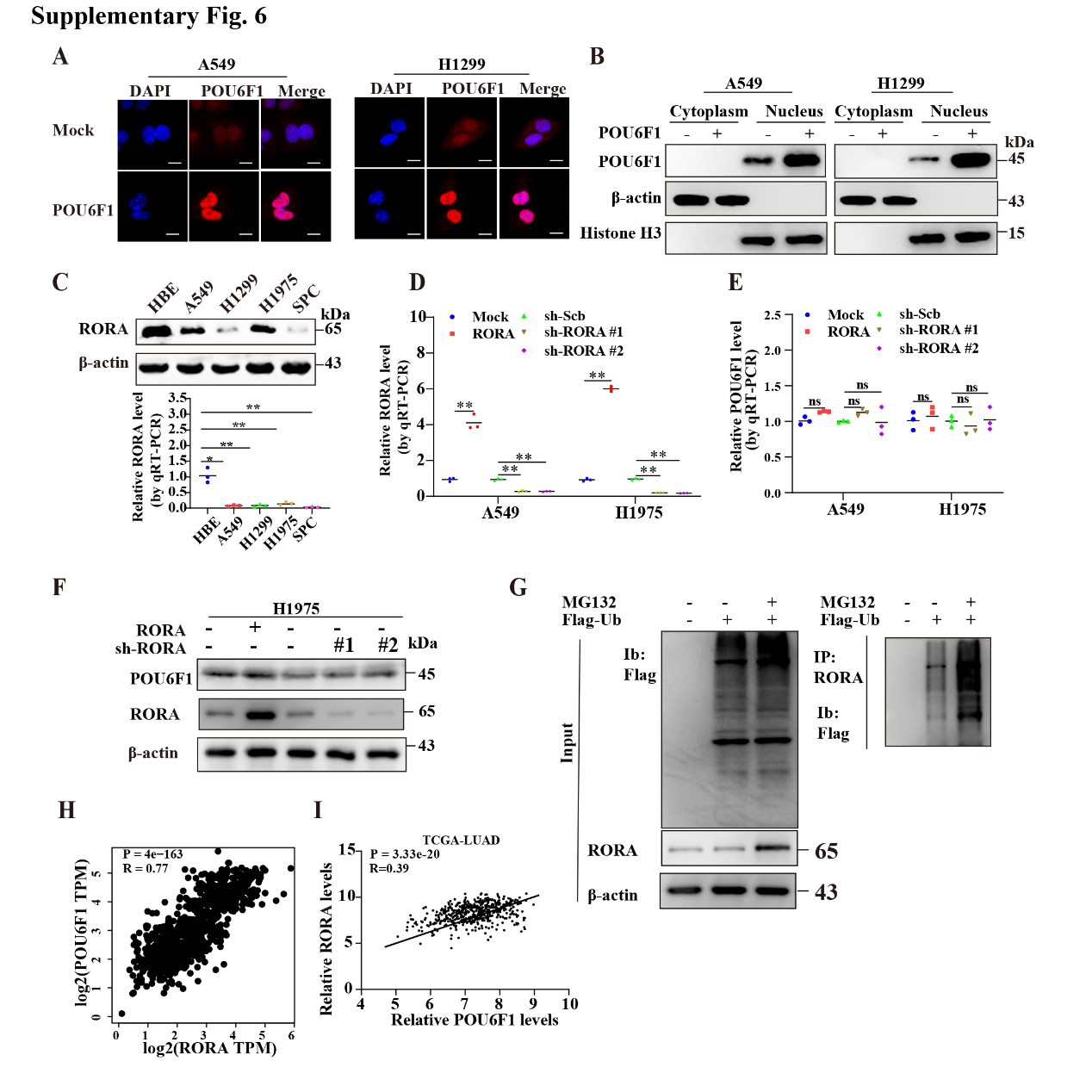
**

**A.** Immunofluorescence assay revealing the location of POU6F1 in A549 and NCI-H1299 cells transfected with empty vector (pCMV-HA) or pCMV-HA-POU6F1. Scale bar: 10µm. **B.** Western blotting assay showing the cytoplasmic and nuclear expression of POU6F1 in A549 and NCI-H1299 cells transfected with mock or POU6F1. **C.** Western blotting and real-time qRT-PCR assays showing the expression of RORA in A549, NCI-H1299, H1975, and SPC compared with HBE. **D-E.** Real-time qRT-PCR assay indicating the expression of RORA and POU6F1 in A549 and H1975 cells transfected with mock, RORA, scramble shRNA (sh-Scb), sh-RORA #1, or sh-RORA #2. **F**. Western blotting showing the expression of RORA and POU6F1 in H1975 cells transfected with mock, RORA, sh-Scb, sh-RORA #1, or sh-RORA #2. **G.** Ubiquitylation assay showing the ubiquitination level of RORA in HEK293T cells transfected with Flag-Ub, and those treated with DMSO or MG132 (5 μmol/l). **H-I.** Public dataset analysis showing the correlation between POU6F1 and RORA using GEPIA and TCGA database. Student’s t-test and ANOVA compared the difference in C-E. Pearson’s correlation coefficient analysis compared the correlation between POU6F1 and RORA in H and I. **P* < 0.05, ***P* < 0.01.

**
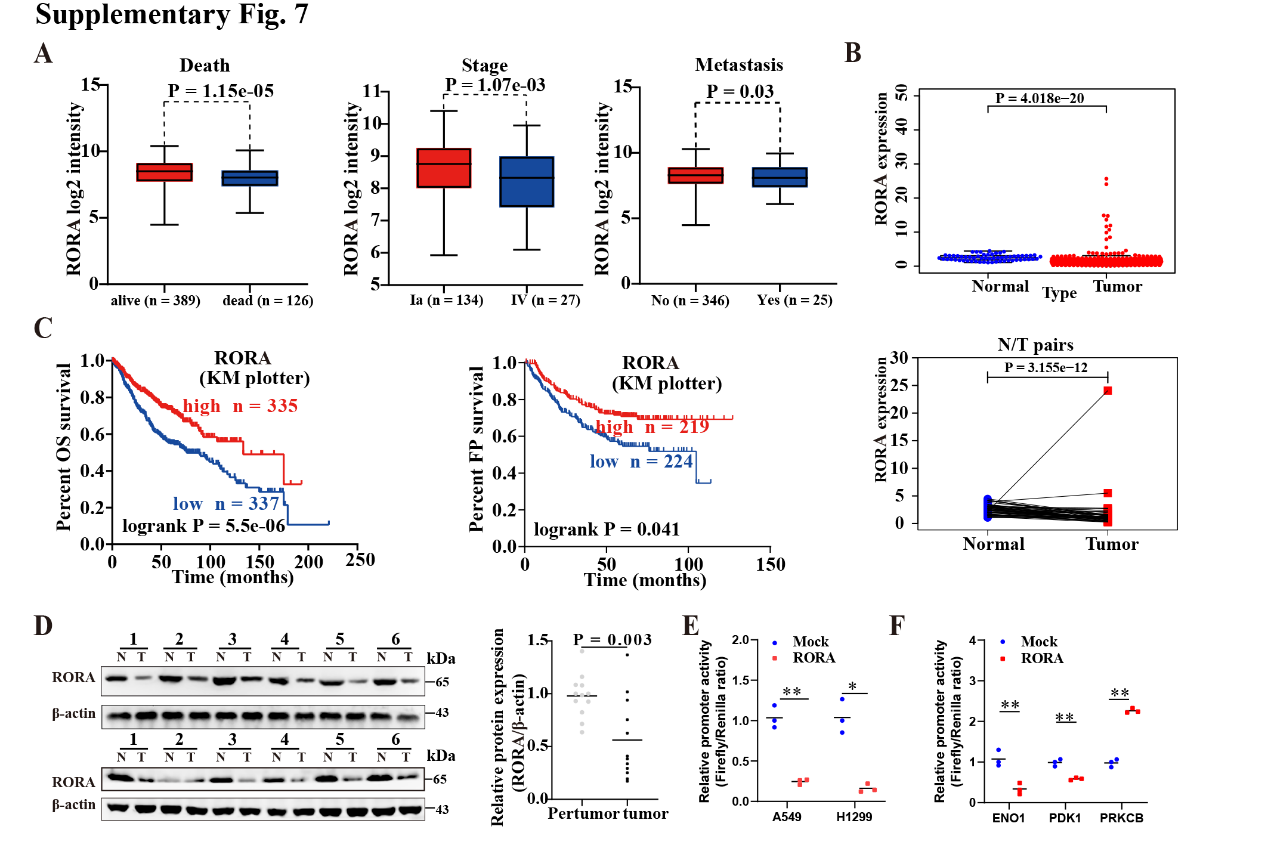
**

**A.** Mining public dataset (TCGA-LUAD) showing the differential level of RORA in LUAD patients with different statuses of death, tumor stage (IV vs. Ia), and metastasis. **B.** Expression level of RORA in unpaired (upper panel) or paired (lower panel) LUAD tissues compared with normal tissues derived from TCGA-LUAD dataset. **C.** Kaplan-Meier curves revealing overall survival (OS) and first progression (FP) of LUAD patients with high or low levels of RORA. **D.** Proteins were extracted from LUAD tissues and adjacent normal tissues and assessed by western blotting assay (D, left panel). The relative protein expression (D, right panel) of RORA was normalized to that of β-actin using ImageJ software. **E-F.** Dual-luciferase assay indicating the relative promoter activity of HIF1A, ENO1, PDK1, and PRKCB in A549 cells transfected with empty vector (mock) or RORA. Log-rank test for survival comparison in C. Student’s t-test and ANOVA compared the difference in D-F **P* < 0.05, ***P* < 0.01.

**
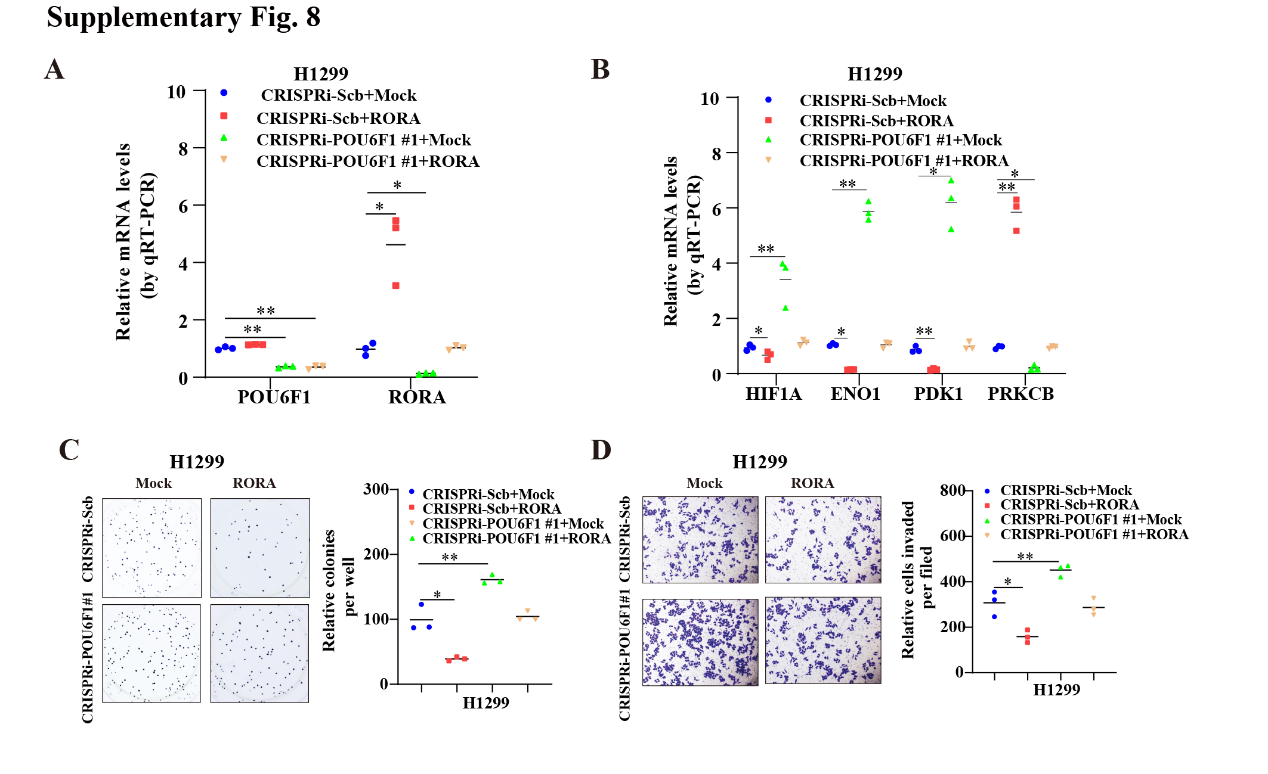
**

**A-B.** Real-time qRT-PCR assay indicating the expression of POU6F1, RORA, HIF1A, ENO1, PDK1, and PRKCB in NCI-H1299 cells transfected with empty vector (mock), RORA, CRISPRi-Scb, or CRISPRi-POU6F1 #1. **C-D.** Representative images (left panel) and quantification (right panel) of soft-agar (C) and transwell (D) assays indicating the growth and invasion of NCI-H1299 cells transfected with mock, RORA, CRISPRi-Scb, or CRISPRi-POU6F1 #1. Student’s t-test and ANOVA compared the difference in A–D. **P* < 0.05, ***P* < 0.01.

**
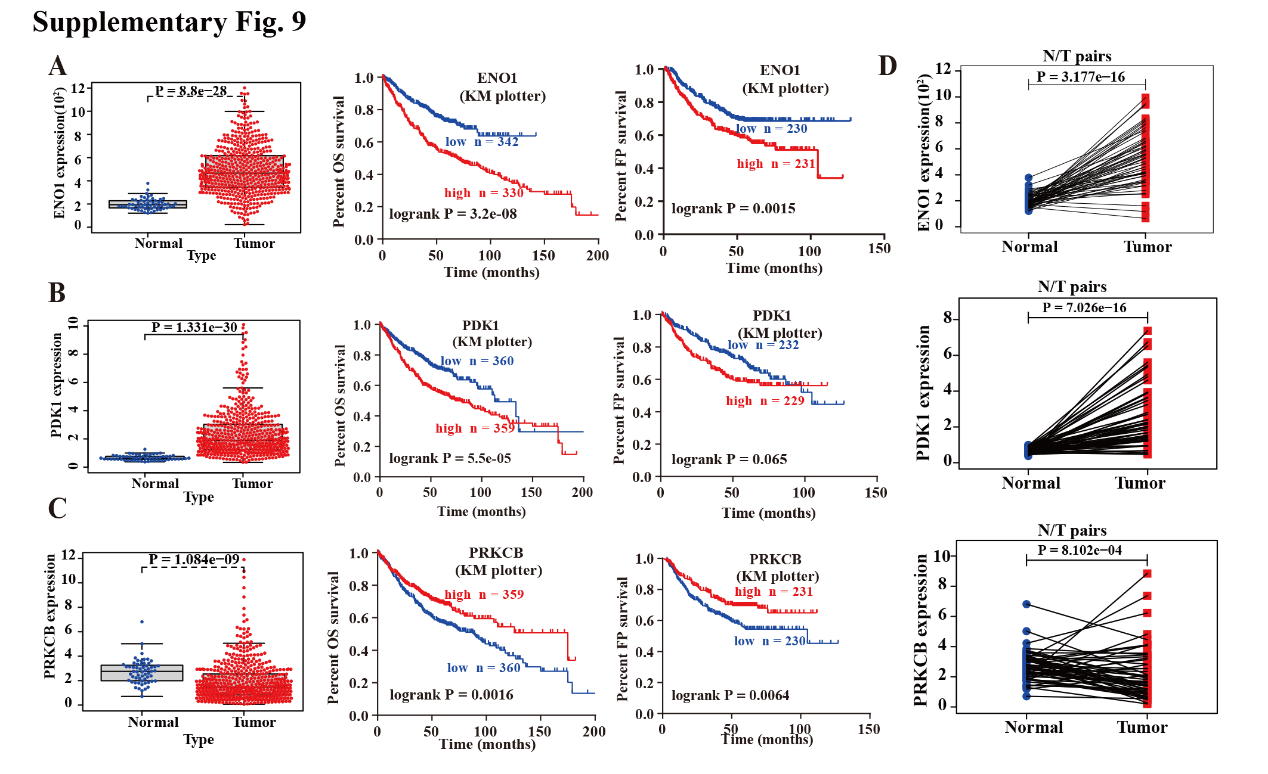
**

**A-C.** The expression (left panel), overall survival (OS, middle panel), and first progression (FP, right panel) of ENO1 (A), PDK1 (B), and PRKCB (C) in LUAD tissues compared with normal tissues. **D.** Expression levels of ENO1 (upper panel), PDK1 (middle panel), and PRKCB (lower panel) in LUAD tissues relative to matched normal tissues.

**Figures and Supplementary Figures**


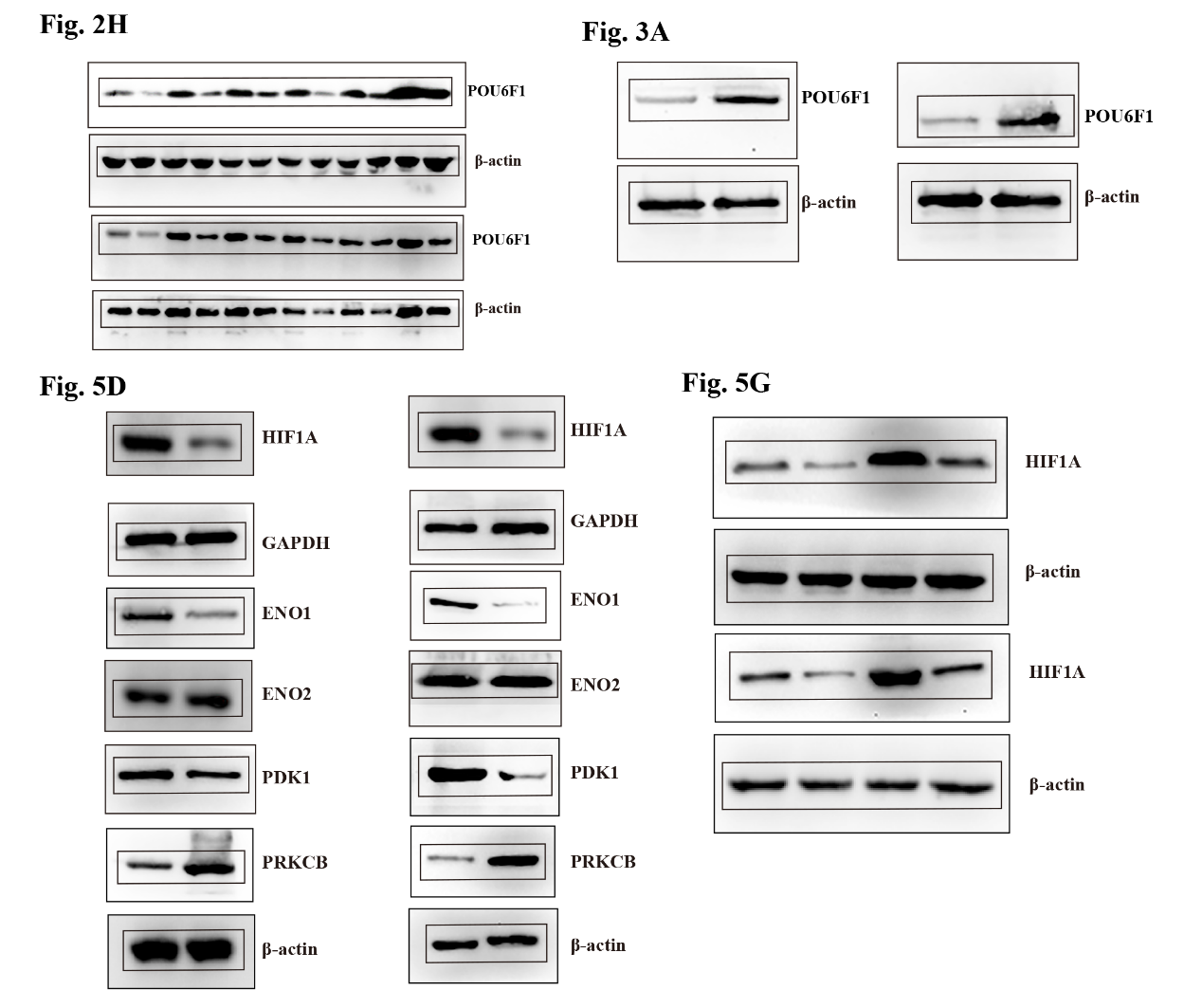


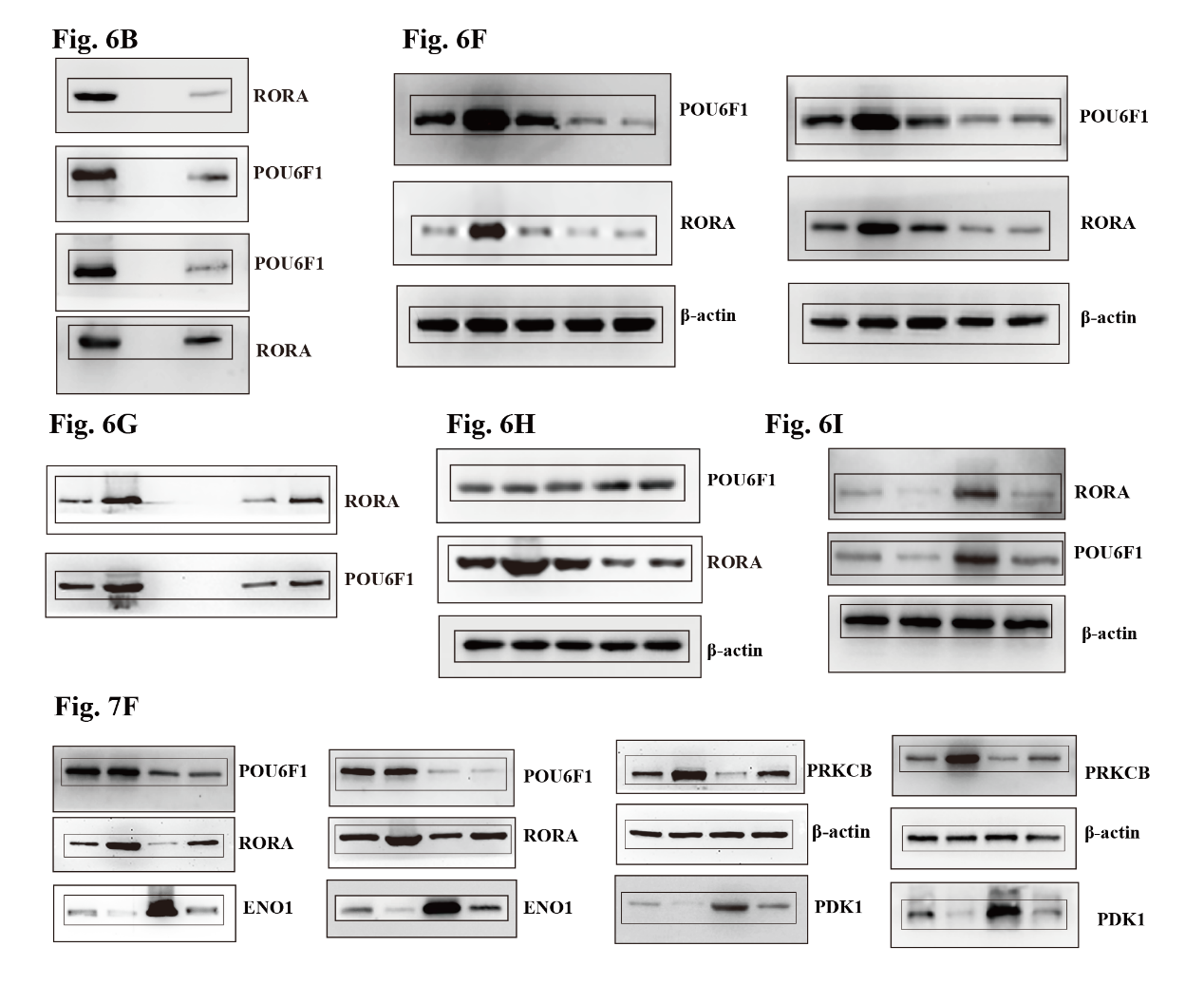


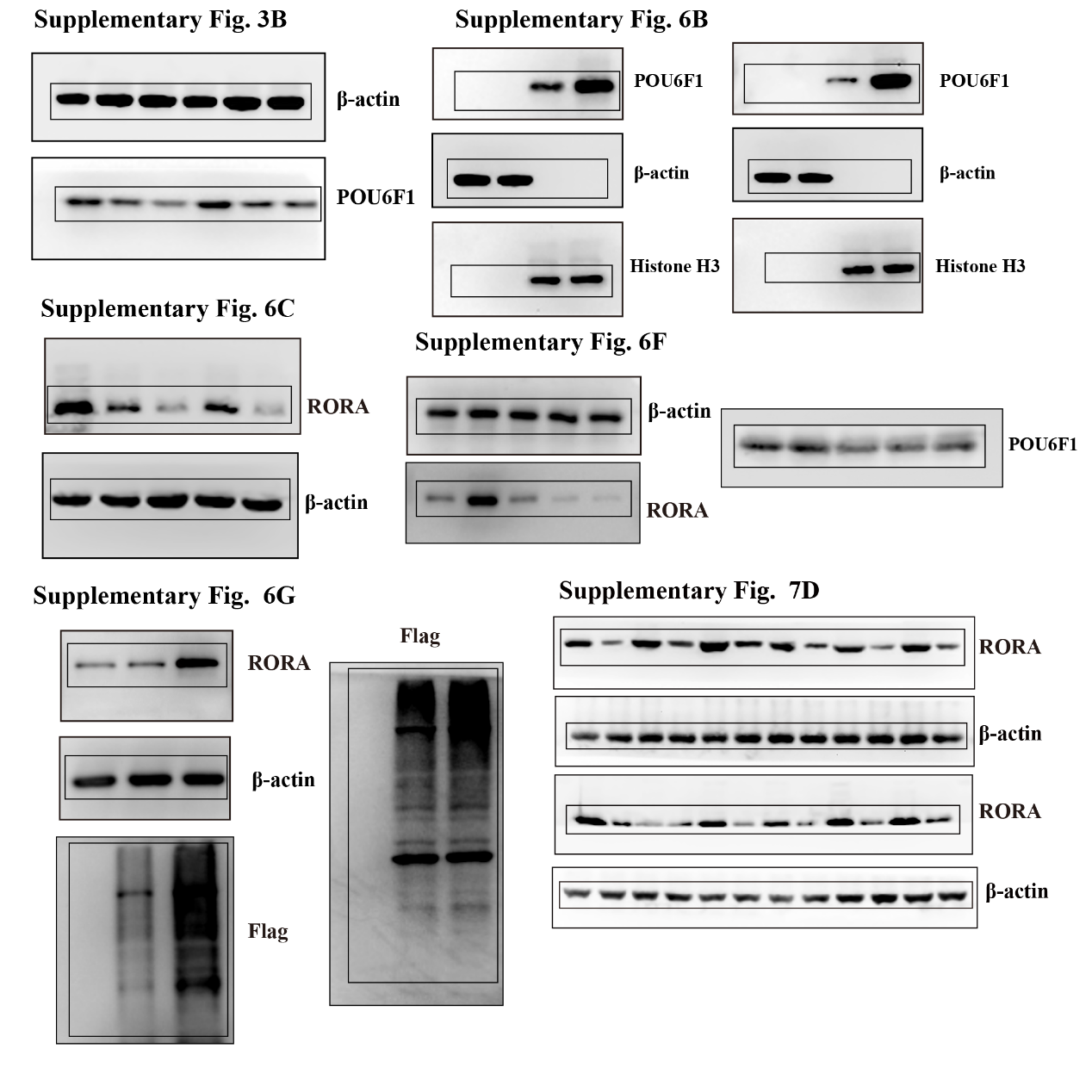


**Supplementary Tables**

**Supplementary Table 1. Transcription factors details list that was associated with the death of LUAD patients**

| 1) CDX4 | 31) FOXM1 | 61) NFATC1 | 91) FOXL2 |
| --- | --- | --- | --- |
| 2) CRX | 32) RFX5 | 62) ZNF189 | 92) STAT3 |
| 3) HSFX1 | 33) IRX6 | 63) ERG | 93) PHF1 |
| 4) HMGA1 | 34) RFXAP | 64) POU3F1 | 94) TEF |
| 5) BTG2 | 35) ARNTL | 65) HCLS1 | 95) ZNF41 |
| 6) ZNF154 | 36) HLF | 66) NFATC2 | 96) FOXN3 |
| 7) ZNF397 | 37) IRF4 | 67) CREBZF | 97) PBX4 |
| 8) RORA | 38) ZNF396 | 68) IRX4 | 98) ZHX3 |
| 9) FOSL1 | 39) YEATS4 | 69) KLF2 | 99) LHX1 |
| 10) ZNF449 | 40) ELF5 | 70) TFEB | 100) XBP1 |
| 11) YBX1 | 41) IRX5 | 71) ATRX | 101) NR2E3 |
| 12) DMTF1 | 42) MYBL2 | 72) HSF5 | 102) HDAC2 |
| 13) NR0B2 | 43) FMNL2 | 73) EGR3 | 103) EOMES |
| 14) CBFA2T3 | 44) HSF1 | 74) ZNF81 | 104) E2F7 |
| 15) IKZF4 | 45) CCRN4L | 75) PKNOX2 | 105) KLF15 |
| 16) TEAD4 | 46) EHF | 76) UBP1 |  |
| 17) POU6F1 | 47) PRDM16 | 77) TRIM22 |  |
| 18) ZNF80 | 48) PHF5A | 78) GAS7 |  |
| 19) VAX1 | 49) NR3C2 | 79) SETD2 |  |
| 20) REL | 50) ARID4A | 80) RUNX1T1 |  |
| 21) TGIF1 | 51) TRIM28 | 81) ZNF70 |  |
| 22) ZNF33A | 52) ZNF19 | 82) EGR2 |  |
| 23) ENO1 | 53) ZNF83 | 83) IRX2 |  |
| 24) ZNF169 | 54) PRDM2 | 84) NFIX |  |
| 25) VENTX | 55) SOX6 | 85) ZNF500 |  |
| 26) ZNF483 | 56) BTAF1 | 86) UHRF1 |  |
| 27) ZNF211 | 57) PFDN1 | 87) IRF8 |  |
| 28) PITX3 | 58) NR2C2 | 88) FOXO4 |  |
| 29) TFAM | 59) ZNF91 | 89) PEG3 |  |
| 30) PA2G4 | 60) MLLT10 | 90) ZSCAN22 |  |

The screened transcription factors (TFs) closely associated with death, derived from a public LUAD dataset of 515 cases.

**Supplementary Table 2. Transcription factors details list that was associated with tumor stage of LUAD patients**

| 1) ARGFX | 11) IRF8 | 21) RUNX3 |
| --- | --- | --- |
| 2) ATF1 | 12) IRX4 | 22) SMC3 |
| 3) ATF2 | 13) MYF5 | 23) SPIB |
| 4) CDX4 | 14) NEUROG1 | 24) TAF5 |
| 5) CRX | 15) NFIB | 25) TBX21 |
| 6) EOMES | 16) NFXL1 | 26) VAV1 |
| 7) FOXP3 | 17) NKX2-2 | 27) VAX1 |
| 8) HCLS1 | 18) PBX4 | 28) ZNF444 |
| 9) HSFX1 | 19) POU2F2 | 29) ZNF80 |
| 10) IRF4 | 20) POU6F1 |  |

The screened transcription factors (TFs) closely associated with tumor stage, derived from a public LUAD dataset of 515 cases.

**Supplementary Table 3. Transcription factors details list that was associated with metastasis of LUAD patients**

| 1) ARID4A | 31) HIRA | 61) SCRT1 | 91) ZNF83 |
| --- | --- | --- | --- |
| 2) ARNT2 | 32) HMGA1 | 62) SETD2 | 92) ZNFX1 |
| 3) ATF1 | 33) HMX3 | 63) SMARCA4 | 93) ZSCAN18 |
| 4) BTAF1 | 34) HOXB3 | 64) ST18 | 94) ZSCAN22 |
| 5) CBFA2T2 | 35) HSFX1 | 65) SUPT4H1 |  |
| 6) CBL | 36) LZTR1 | 66) TAF13 |  |
| 7) CDX4 | 37) MGA | 67) TARDBP |  |
| 8) CITED1 | 38) MLX | 68) TBX19 |  |
| 9) CNBP | 39) MTF1 | 69) TEAD1 |  |
| 10) CNOT8 | 40) MZF1 | 70) TEF |  |
| 11) CREB3 | 41) NFAT5 | 71) TFAM |  |
| 12) CREBZF | 42) NFIA | 72) TULP4 |  |
| 13) CREM | 43) NFIL3 | 73) YEATS4 |  |
| 14) CRX | 44) NME2 | 74) ZHX3 |  |
| 15) DLX1 | 45) NR2C2 | 75) ZKSCAN1 |  |
| 16) DMTF1 | 46) NR2E3 | 76) ZNF134 |  |
| 17) E2F5 | 47) PAX6 | 77) ZNF135 |  |
| 18) ELF5 | 48) PEG3 | 78) ZNF154 |  |
| 19) ESR2 | 49) PFDN1 | 89) ZNF175 |  |
| 20) ETV6 | 50) PHF5A | 80) ZNF18 |  |
| 21) FMNL2 | 51) PITX2 | 81) ZNF202 |  |
| 22) FOXC1 | 52) POU3F1 | 82) ZNF207 |  |
| 23) FOXD1 | 53) POU6F1 | 83) ZNF211 |  |
| 24) FOXI1 | 54) PRDM2 | 84) ZNF256 |  |
| 25) FOXJ3 | 55) PURB | 85) ZNF274 |  |
| 26) FOXK1 | 56) RB1 | 86) ZNF33A |  |
| 27) FOXN3 | 57) REL | 87) ZNF37A |  |
| 28) GATAD2B | 58) RERE | 88) ZNF445 |  |
| 29) GTF2A2 | 59) RUNX1 | 89) ZNF70 |  |
| 30) HAND2 | 60) SCMH1 | 90) ZNF81 |  |

The screened transcription factors (TFs) closely associated with metastasis, derived from a public LUAD dataset of 515 cases.

**Supplementary Table 4. Primer sets used for qRT-PCR and ChIP**

| **Primer set** | **Primers** | **Sequence** | **Product size (bp)** | **Application** |
| --- | --- | --- | --- | --- |
| POU6F1 | Forward | 5’-TGCTCAGGGACAGGTTATTGG-3' | 201 | qRT-PCR |
|  | Reverse | 5’-GACTCAGGTGTGCTTGGCTTC-3' |  |  |
| GAPDH | Forward | 5’-ATGTCCCAGCTCTCCTCCACC-3' | 160 | qRT-PCR |
|  | Reverse | 5’-CTACATTCGGGAGGGCGGGCT-3' |  |  |
| β-actin | Forward | 5'-TGCCCATCTACGAGGGGTATG-3’ | 156 | qRT-PCR |
|  | Reverse | 5'-TCTCCTTAATGTCACGCACGATTT-3’ |  |  |
| RORA | Forward | 5'-GATGCTTTTGTTCTTACTGGCG-3’ | 220 | qRT-PCR |
|  | Reverse | 5'-CATTGCTTTGCTGACTTCTCCT-3’ |  |  |
| ENO1 | Forward | 5'-GCCTCCTGCTCAAAGTCAAC-3’ | 102 | qRT-PCR |
|  | Reverse | 5'-AACGATGAGACACCATGACG-3’ |  |  |
| ENO2 | Forward | 5'-GTCATCAAGGACAAATACGGCAAG-3’ | 174 | qRT-PCR |
|  | Reverse | 5'-ATAAAACTCTGAGGCAGCAACATC-3’ |  |  |
| PDK1 | Forward | 5'-TGGTTTTGGTTATGGATTGCCC-3’ | 194 | qRT-PCR |
|  | Reverse | 5'-CAGCCTCGTGGTTGGTGTTGTA-3’ |  |  |
| PRKCB | Forward | 5'- AGCCCCACGTTTTGTGACC-3’ | 117 | qRT-PCR |
|  | Reverse | 5'-GCTGGGAACATTCATCACGC-3’ |  |  |
| HIF1A | Forward | GCCTTGGATGGTTTTGTTATGGTT | 216 | qRT-PCR |
|  | Reverse | GCTTCGCTGTGTGTTTTGTTCTTT |  |  |
| ENO1 (-991/-853) | Forward | TCCAACTCCTTCCGTATTCCAC | 284 | ChIP |
|  | Reverse | TTACATCCCTCTCATTCCCATC |  |  |
| PDK1 (-922/-680) | Forward | AACCGCCAAAAACCGTTTTCTC | 242 | ChIP |
|  | Reverse | AGCCTCCGTCTCTTTACCTGAA |  |  |
| PRKCB (-1234/-1034) | Forward | CATAGGGTTGTTGCGGGGGCTA | 170 | ChIP |
|  | Reverse | TATTGGTTGATTGAGTGAGGGC |  |  |

POU6F1, POU domain, class 6, transcription factor 1; ACTB, beta-actin; GAPDH, glyceraldehyde 3-phosphate dehydrogenase; RORA, retinoid-related orphan receptor alpha; ENO1, enolase 1; ENO2, enolase 2; PDK1, pyruvate dehydrogenase kinase 1; PRKCB, protein kinase C beta; and HIF1A, hypoxia inducible factor 1, alpha subunit; ChIP, chromatin immunoprecipitation.

**Supplementary Table 5. Oligonucleotide sets used for constructs and short hairpin RNAs**

| **Oligo Set** | **Sequences** |
| --- | --- |
| POU6F1-CV186 | 5'-GCTGCAGGTCGACTCTAGAGGATCCATGGATCCTGGAGCCGGGTCA-3' (Forward) |
|  | 5'-ACTGACACACATTCCACAGGCTAGCCTAAGGGATCTGAAAGACGTT-3' (Reverse); |
| pCMV-HA -POU6F1 | 5'-CCGGAATTCGGATGGATCCTGGAGCCGGGTCA-3' (Forward) |
|  | 5'-CCGCTCGAGCTAAGGGATCTGAAAGACGTT-3' (Reverse); |
| sgRNA-CRISPRi-POU6F1 #1 | 5'-CACCGATACATGTAAAAATTTATCAAGG-3' (Forward) |
|  | 5'-AAACCCTTGATAAATTTTTACATGTATC-3' (Reverse); |
| sgRNA-CRISPRi-POU6F1 #2 | 5'-CACCGTTTTGTTTCTGTGTTTATTGGGG-3' (Forward) |
|  | 5'-AAACCCCCAATAAACACAGAAACAAAAC-3' (Reverse); |
| sh-Scb | 5'-CCGGTTCTCCGAACGTGTCACGTCTCGAGACGTGACACGTTCGGAGAATTTTTG-3' (Forward) |
|  | 5'-GATCCAAAAATTCTCCGAACGTGTCACGTCTCGAGACGTGACACGTTCGGAGAA-3' (Reverse); |
| sh-POU6F1 #1 | 5'-CCGGTGAACACCAGCAAGCTGAACCTCGAGGTTCAGCTTGCTGGTGTTCTTTTTG-3' (Forward) |
|  | 5'-GATCCAAAAAGAACACCAGCAAGCTGAACCTCGAGGTTCAGCTTGCTGGTGTTCA-3' (Reverse); |
| sh-POU6F1 #2 | 5'-CCGGTGCCAAGCACACCTGAGTCCCTCGAGGGACTCAGGTGTGCTTGGCTTTTTG-3' (Forward) |
|  | 5'-GATCCAAAAAGCCAAGCACACCTGAGTCCCTCGAGGGACTCAGGTGTGCTTGGCA-3' (Reverse); |
| sh-RORA #1 | 5'-CCGGTGAGCCAGAAGGGATGAACTTTCTCGAGAAAGTTCATCCCTTCTGGCTCTTTTTG-3' (Forward) |
|  | 5'-GATCCAAAAAGAGCCAGAAGGGATGAACTTTCTCGAGAAAGTTCATCCCTTCTGGCTCA-3' (Reverse); |
| sh-RORA #2 | 5'-CCGGTCCGGATGCAGCAGCAGCAGCTCGAGCTGCTGCTGCTGCATCCGGTTTTTG-3' (Forward) |
|  | 5'-GATCCAAAAACCGGATGCAGCAGCAGCAGCTCGAGCTGCTGCTGCTGCATCCGGA-3' (Reverse); |
| RORA-CV186 | 5'-ATGAATGAAGGAGCACCTGGCG-3' (Forward) |
|  | 5'-CCCATCAATTTGCATTGCTGGC-3' (Reverse); |
| pCMV-3Tag-1-RORA | 5'-CGCGGATCCATGAATGAAGGAGCACCTGG-3' (Forward) |
|  | 5'-CCCAAGCTTCTACCCATCAATTTGCATTG-3' (Reverse); |
| pBIFC-POU6F1-VC155 | 5'-CCCGAATTCGCATGGATCCTGGAGCCGGGTCAG-3' (Forward) |
|  | 5'-CGGGGTACCAGGGATCTGAAAGACGTTCAGCTTG-3' (Reverse); |
| pBIFC-RORA-VN173 | 5'-CCCAAGCTTATGAATGAAGGAGCACCTGGCG-3' (Forward) |
|  | 5'-CGGGGTACCGCCCCATCAATTTGCATTGCTGGC-3' (Reverse) |

**Supplementary Table 6. Correlation between POU6F1 expression and clinical parameters in LUAD patients**

| **Parameter** |  | **Number** | **POU6F1 expression** | | **P value** |
| --- | --- | --- | --- | --- | --- |
|  |  |  | **Low (n=257)** | **High (n=258)** |  |
| Age | <=65 | 238 | 120 | 118 | 0.8602 |
|  | >65 | 258 | 127 | 131 |  |
|  | unknow | 19 | 10 | 9 |  |
| Gender | female | 277 | 152 | 125 | 0.019 |
|  | male | 238 | 105 | 133 |  |
| Tumor stage | I-II | 403 | 214 | 189 | 0.006 |
|  | III-IV | 111 | 42 | 69 |  |
| T stage | T 1-2 | 446 | 231 | 215 | 0.027 |
|  | T 3-4 | 66 | 24 | 42 |  |
|  | unknow | 3 | 2 | 1 | 0.012 |
| N stage | N0+NX | 343 | 185 | 158 |  |
|  | N1 | 172 | 72 | 100 |  |
| M stage | M0+MX | 490 | 249 | 241 | 0.103 |
|  | M1 | 25 | 8 | 17 |  |

**Supplementary Table 7. Mass spectrometry (MS) analysis of POU6F1-interacting proteins**

**MS analysis of POU6F1-interacting proteins in A549 cells**

| \| RORA \| \| --- \| \| POU6F1 \| \| KRT18 \| \| ZC3HAV1 \| \| FARP2 \| \| SYF2 \| \| KIF2C \| \| MCRIP2 \| \| DDX41 \| \| SLIRP \| \| DDX50 \| \| RPL17 \| \| UPF3B \| \| STAU1 \| \| AP2B1 \| \| SRSF7 \| \| DDX3X \| \| PHF6 \| \| PUF60 \| \| HLA-A \| \| C4A \| \| DDX17 \| \| HBB \| \| MOGS \| \| RGPD3 \| \| MATR3 \| \| EWSR1 \| \| HNRNPC \| \| MSI2 \| \| CARS1 \| \| HNRNPLL \| \| ANKZF1 \| \| ELOB \| \| FGG \| \| CYCS \| \| MBNL1 \| \| TMEM33 \| \| EIF4E \| \| CSNK1A1 \| \| EXOSC9 \| \| TBCA \| \| AP3M2 \| \| PRRC2C \| \| EZR \| \| RBMS1 \| \| SEPTIN10 \| \| LAMB1 \| \| CALD1 \| \| PPIE \| \| LMO7 \| \| SF3B2 \| \| EEF1D \| \| SEC16A \| \| MYO1C \| \| MRE11 \| \| CPSF6 \| \| NUP160 \|   SEC24C  ANAPC1   \| L1RE1 \| \| --- \| \| STUB1 \| \| LAMTOR2 \| \|  \| | \| PABPC4 \| \| --- \| \| ESRP1 \| \| PUM1 \| \| CRIP2 \| \| ATXN2 \| \| MAN2A2 \| \| ATXN2L \| \| RNPS1 \| \| SLTM \| \| DHX30 \| \| ITIH4 \| \| CP \| \| SRSF2 \| \| HP \| \| SNRPN \| \| NUDT16L1 \| \| RPS15 \| \| DAZAP1 \| \| EXOSC5 \| \| ZNF841 \| \| FBL \| \| SUGP2 \| \| GTPBP1 \| \| IGF2BP3 \| \| KPNA3 \| \| KMT2D \| \| UQCRQ \| \| YKT6 \| \| DHX15 \| \| PRPF4 \| \| SART1 \| \| PLRG1 \| \| ZNF207 \| \| NUDT21 \| \| RAD21 \| \| KPNA6 \| \| DKC1 \| \| DNAJA2 \| \| PRPF40A \| \| SF3B1 \| \| PRKRA \| \| SNRNP200 \| \| ARL6IP5 \| \| BCAS2 \| \| SMNDC1 \| \| FLOT1 \| \| SRP72 \| \| PRPF6 \| \| PTBP3 \| \| AP2A1 \| \| BAG2 \| \| SERPINA1 \| \| A2M \| \| C3 \| \| IGHG1 \| \| IGHG2 \| \| APOA1 \| \| APOC3 \| \| FGA \| \| FGB \|   AKAP8L  SNX9 | \| ORM1 \| \| --- \| \| TF \| \| HPX \| \| CAT \| \| HRG \| \| APOA4 \| \| GPX1 \| \| SNRPB2 \| \| SNRNP70 \| \| VIM \| \| CLTA \| \| CLTB \| \| HNRNPA1 \| \| SNRPA1 \| \| HSPA8 \| \| PABPC1 \| \| HNRNPL \| \| AKR1B1 \| \| GSPT1 \| \| DDX5 \| \| LGALS3 \| \| TCN1 \| \| PCMT1 \| \| SFPQ \| \| EIF4B \| \| EEF1B2 \| \| MSN \| \| DDX6 \| \| U2AF2 \| \| PTBP1 \| \| VARS1 \| \| EEF1G \| \| EEF1D \| \| HNRNPH3 \| \| HNRNPH1 \| \| KIF5B \| \| HSPA4 \| \| PPM1A \| \| SRP14 \| \| EIF4A3 \| \| RPL3 \| \| BUD31 \| \| MTREX \| \| RANGAP1 \| \| CRKL \| \| RPS9 \| \| PRRC2A \| \| RBM25 \| \| RANBP2 \| \| FXR1 \| \| FXR2 \| \| RAB9A \| \| HNRNPA3 \| \| HNRNPM \| \| NCBP2 \| \| HNRNPF \| \| NUP98 \| \| AP2S1 \| \| VCP \| \| AFDN  LSM2  MED31 \| | \| ADAR \| \| --- \| \| SEC13 \| \| HNRNPH2 \| \| RRP1 \| \| NUP107 \| \| WDR5 \| \| HNRNPK \| \| SNRPE \| \| SNRPF \| \| LSM3 \| \| SNRPD1 \| \| SNRPD2 \| \| SNRPD3 \| \| RPS4X \| \| TRA2B \| \| PPP2R2A \| \| SUMO1 \| \| DYNLL1 \| \| HBA1 \| \| RAE1 \| \| SARNP \| \| SRSF3 \| \| PURA \| \| CLTC \| \| U2AF1 \| \| EXOSC10 \| \| OTUD4 \| \| EIF4G1 \| \| YWHAH \| \| EEF1A2 \| \| FMR1 \| \| SRSF1 \| \| DHX9 \| \| GOLGA2 \| \| NCBP1 \| \| AHNAK \| \| CPSF1 \| \| SF3A3 \| \| MYO1E \| \| TARDBP \| \| HNRNPA0 \| \| SRSF9 \| \| SRSF5 \| \| SRSF6 \| \| G3BP1 \| \| PABPC4 \| \| SQSTM1 \| \| TRA2A \| \| EXOSC2 \| \| CIRBP \| \| HNRNPD \| \| SAFB2 \| \| GIT2 \| \| MAP7 \| \| TRIM25 \| \| CAPRIN1 \| \| RBM39 \| \| ITPR3 \| \| SMC1A  PPME1  SUPT16H  TIMM13 \| | \| USP10 \| \| --- \| \| EXOSC7 \| \| EFTUD2 \| \| PLEC \| \| NONO \| \| RBBP5 \| \| SF3B3 \| \| CNN3 \| \| SAFB \| \| SF3B4 \| \| SF3A2 \| \| SF3A1 \| \| TRIP6 \| \| ELAVL1 \| \| DBN1 \| \| SMU1 \| \| LSM12 \| \| SNRPG \| \| HSP90AB2P \| \| ZNF326 \| \| SRSF10 \| \| RRP12 \| \| CRNKL1 \| \| EXOSC6 \| \| CDC40 \| \| HP1BP3 \| \| SRSF11 \| \| HMGB1 \| \| ATAD3B \| \| ZCCHC8 \| \| EDC4 \| \| PRPF8 \| \| LARP1 \| \| FIP1L1 \| \| RPS27L \| \| PHF5A \| \| NUFIP2 \| \| KTN1 \| \| ALYREF \| \| THOC6 \| \| MISP \| \| CHERP \| \| CCAR1 \| \| CCAR2 \| \| LRRC47 \| \| STAG2 \| \| MINK1 \| \| CPSF7 \| \| LSM14A \| \| THOC2 \| \| NUP133 \| \| PSPC1 \| \| DDX1 \| \| TAF15 \| \| RAD50 \| \| UPF1 \| \| ARHGEF2 \| \| ZNF622 \| \| MAGOHB \| \| FAF2 \| \| CCDC124  PFDN2 \| | \| SNRNP40 \| \| --- \| \| DNAJA3 \| \| PTCD3 \| \| MED30 \| \| PGAM5 \| \| FUBP3 \| \| THOC3 \| \| ZFR \| \| RBM14 \| \| PURB \| \| NIBAN2 \| \| CDC5L \| \| TRIR \| \| GRWD1 \| \| MMTAG2 \| \| SF3B5 \| \| SRRT \| \| EIF2A \| \| POLDIP3 \| \| TBL1XR1 \| \| PAIP1 \| \| XRN2 \| \| DHX36 \| \| PNN \| \| WDR26 \| \| MOV10 \| \| EXOSC4 \| \| ATAD3A \| \| TMOD3 \| \| FAM120A \| \| IGF2BP1 \| \| MYOF \| \| CWC15 \| \| ATXN10 \| \| NXF1 \| \| CPSF3 \| \| NUDT5 \| \| RALY \| \| GTF3C4 \| \| AGO2 \| \| PRPF19 \| \| G3BP2 \| \| SRRM2 \| \| SMC3 \| \| RUVBL1 \| \| SF3B6 \| \| PPIL1 \| \| RTCB \| \| LSM5 \| \| RBM7 \| \| YTHDF2 \| \| RBM8A \| \| EXOSC1 \| \| EMG1 \| \| YTHDF3 \| \| BAG6 \| \| CEP43 \| \| PEG10 \| \| AP2M1  IGF2BP2  EMC4  ACIN1 \| | \| RNF114 \| \| --- \| \| WDR47 \| \| PRPF38B \| \| WDR36 \| \| DDX39A \| \| UBE2A \| \| MRPS18B \| \| RBFOX2 \| \| PACSIN2 \| \| MED17 \| \| HNRNPA1P48 \| \| CLPB \| \| IMPDH1 \| \| UROD \| \| RBM15 \| \| MED27 \| \| CTNND1 \| \| ARHGEF7 \| \| CLK2 \| \| HNRNPC \| \| NCOA1 \| \| RTN3 \| \| PPIL3 \| \| EIF4E2 \| \| CUTA \| \| MKRN2 \| \| PALM2AKAP2 \| \| LPP \| \| POP7 \| \| MKRN1 \| \| CDV3 \| \| UBE2D3 \| \| CCNC \| \| STAU2 \| \| COPS6 \| \| UBR5 \| \| U2SURP \| \| PRPF31 \| \| SMC4 \| \| WDR6 \| \| MED22 \| \| EEF1D \| \| EEF1D \| \| ZC3H11A \| \| LSM8 \| \| VPS37B \| \| PAAF1 \| \| QKI \| \| SLC25A10 \| \| LARP4 \| \| RBMS2 \| \| ECI2 \| \| SACM1L \| \| ZC3H14 \| \| SERPINA3 \| \| KLC1 \| \| IBTK \| \| DLAT \| \| YARS2  MED13 \|   DDX20 | \| CERS2 \| \| --- \| \| SRPK1 \| \| JPT2 \| \| ARPP19 \| \| MT1G \| \| AAAS \| \| PDIA3 \| \| MRM3 \| \| TSR1 \| \| GEMIN4 \| \| MAZ \| \| MED19 \| \| NUP85 \| \| MPDU1 \| \| BAG1 \| \| POLRMT \| \| GLYR1 \| \| HS2ST1 \| \| SARS2 \| \| POLD1 \| \| KIF2A \| \| SAP18 \| \| ZNF593 \| \| PPP6C \| \| UBE2C \| \| CHD1 \| \| LSM1 \| \| NKRF \| \| CASC3 \| \| PPM1G \| \| STX7 \| \| POLR1G \| \| AKR7A2 \| \| MED7 \| \| PRC1 \| \| MED14 \| \| AQR \| \| BUB1B \| \| NBN \| \| DUSP11 \| \| MED24 \| \| PSIP1 \| \| MED6 \| \| AP2A2 \| \| LUC7L3 \| \| CPSF4 \| \| ECEL1 \| \| BAG3 \| \| TOP3B \| \| MT-CYB \| \| APOB \| \| HNRNPC \| \| UQCRH \| \| SNRPA \| \| LTA4H \| \| LAMP1 \| \| SON \| \| PTMS \| \| SCP2 \| \| POLR2A  DHRS7 \| | \| PSMB4 \| \| --- \| \| PPIF \| \| ADSS2 \| \| POLR2B \| \| GPD2 \| \| PTDSS1 \| \| PSMB2 \| \| YLPM1 \| \| RIDA \| \| METAP1 \| \| CLTCL1 \| \| MFAP1 \| \| CKS1B \| \| VBP1 \| \| DCAF7 \| \| YPEL5 \| \| NOP14 \| \| MRPS21 \| \| LACTB \| \| RBM10 \| \| REEP5 \| \| KMT2A \| \| AP1B1 \| \| ILK \| \| GOLGA4 \| \| MED21 \| \| NAE1 \| \| SNW1 \| \| THOC5 \| \| TRIM29 \| \| NCOA6 \| \| WTAP \| \| MAPRE2 \| \| SF1 \| \| MED1 \| \| MLF2 \| \| ACTBL2 \| \| HSP90AB4P \| \| SNX5 \| \| SH3BGRL3 \| \| HNRNPK \| \| MRPS2 \| \| LMNA \| \| CEP350 \| \| ATP5F1EP2 \| \| RBM17 \| \| VIRMA \| \| THOC7 \| \| ALKBH5 \| \| WDR82 \| \| GIGYF2 \| \| PAXIP1 \| \| MED12 \| \| MYH14 \| \| WAPL \| \| TMED4 \| \| IRF2BP1 \| \| RBM45 \| \| TBC1D10C  MED31 \|   RRP15 | \| MICU2 \| \| --- \| \| CMTR1 \| \| C12orf29 \| \| FAM98A \| \| LINC01006 \| \| UHMK1 \| \| GEMIN5 \| \| CCDC12 \| \| RNF138 \| \| GEMIN6 \| \| BRI3BP \| \| CTNNBL1 \| \| LARP4B \| \| TFG \| \| DCPS \| \| TRIM11 \| \| MED8 \| \| FYTTD1 \| \| MED15 \| \| WRNIP1 \| \| RANBP9 \| \| CLCC1 \| \| STRBP \| \| RGPD5 \| \| COPS4 \| \| CIZ1 \| \| PAGR1 \| \| MED10 \| \| NDUFAF3 \| \| RPP25 \| \| UTP14A \| \| KIFC1 \| \| TARS2 \| \| YTHDF1 \| \| WDR11 \| \| DPY30 \| \| WDR33 \| \| MED28 \| \| EHD4 \| \| ACBD3 \| \| C8orf33 \| \| PHAX \| \| MED20 \| \| AGO3 \| \| XAB2 \| \| PREB \| \| MED4 \| \| NIT2 \| \| ANLN \| \| DIABLO \| \| LANCL2 \| \| KLC4 \| \| ZCCHC3 \| \| RBM22 \| \| MED9 \| \| OCIAD1 \| \| HYPK \| \| MED11 \| \| CPSF2  TFIP11 \|   ASH2L |
| --- | --- | --- | --- | --- | --- | --- | --- | --- | --- | --- | --- | --- | --- | --- | --- | --- | --- | --- | --- | --- | --- | --- | --- | --- | --- | --- | --- | --- | --- | --- | --- | --- | --- | --- | --- | --- | --- | --- | --- | --- | --- | --- | --- | --- | --- | --- | --- | --- | --- | --- | --- | --- | --- | --- | --- | --- | --- | --- | --- | --- | --- | --- | --- | --- | --- | --- | --- | --- | --- | --- | --- | --- | --- | --- | --- | --- | --- | --- | --- | --- | --- | --- | --- | --- | --- | --- | --- | --- | --- | --- | --- | --- | --- | --- | --- | --- | --- | --- | --- | --- | --- | --- | --- | --- | --- | --- | --- | --- | --- | --- | --- | --- | --- | --- | --- | --- | --- | --- | --- | --- | --- | --- | --- | --- | --- | --- | --- | --- | --- | --- | --- | --- | --- | --- | --- | --- | --- | --- | --- | --- | --- | --- | --- | --- | --- | --- | --- | --- | --- | --- | --- | --- | --- | --- | --- | --- | --- | --- | --- | --- | --- | --- | --- | --- | --- | --- | --- | --- | --- | --- | --- | --- | --- | --- | --- | --- | --- | --- | --- | --- | --- | --- | --- | --- | --- | --- | --- | --- | --- | --- | --- | --- | --- | --- | --- | --- | --- | --- | --- | --- | --- | --- | --- | --- | --- | --- | --- | --- | --- | --- | --- | --- | --- | --- | --- | --- | --- | --- | --- | --- | --- | --- | --- | --- | --- | --- | --- | --- | --- | --- | --- | --- | --- | --- | --- | --- | --- | --- | --- | --- | --- | --- | --- | --- | --- | --- | --- | --- | --- | --- | --- | --- | --- | --- | --- | --- | --- | --- | --- | --- | --- | --- | --- | --- | --- | --- | --- | --- | --- | --- | --- | --- | --- | --- | --- | --- | --- | --- | --- | --- | --- | --- | --- | --- | --- | --- | --- | --- | --- | --- | --- | --- | --- | --- | --- | --- | --- | --- | --- | --- | --- | --- | --- | --- | --- | --- | --- | --- | --- | --- | --- | --- | --- | --- | --- | --- | --- | --- | --- | --- | --- | --- | --- | --- | --- | --- | --- | --- | --- | --- | --- | --- | --- | --- | --- | --- | --- | --- | --- | --- | --- | --- | --- | --- | --- | --- | --- | --- | --- | --- | --- | --- | --- | --- | --- | --- | --- | --- | --- | --- | --- | --- | --- | --- | --- | --- | --- | --- | --- | --- | --- | --- | --- | --- | --- | --- | --- | --- | --- | --- | --- | --- | --- | --- | --- | --- | --- | --- | --- | --- | --- | --- | --- | --- | --- | --- | --- | --- | --- | --- | --- | --- | --- | --- | --- | --- | --- | --- | --- | --- | --- | --- | --- | --- | --- | --- | --- | --- | --- | --- | --- | --- | --- | --- | --- | --- | --- | --- | --- | --- | --- | --- | --- | --- | --- | --- | --- | --- | --- | --- | --- | --- | --- | --- | --- | --- | --- | --- | --- | --- | --- | --- | --- | --- | --- | --- | --- | --- | --- | --- | --- | --- | --- | --- | --- | --- | --- | --- | --- | --- | --- | --- | --- | --- | --- | --- | --- | --- | --- | --- | --- | --- | --- | --- | --- | --- | --- | --- | --- | --- | --- | --- | --- | --- | --- | --- | --- | --- | --- | --- | --- | --- | --- | --- | --- | --- | --- | --- | --- | --- | --- | --- | --- | --- | --- | --- | --- | --- | --- | --- | --- | --- | --- | --- | --- | --- | --- | --- | --- | --- | --- | --- | --- | --- | --- | --- | --- | --- | --- | --- | --- | --- | --- | --- | --- | --- | --- | --- | --- | --- | --- | --- | --- | --- | --- | --- | --- | --- | --- | --- | --- | --- | --- | --- | --- | --- | --- | --- | --- | --- | --- | --- | --- | --- | --- | --- | --- | --- | --- | --- | --- | --- | --- | --- | --- | --- | --- | --- | --- | --- | --- | --- | --- | --- | --- | --- | --- | --- | --- | --- | --- | --- | --- | --- | --- | --- |

**MS analysis of POU6F1-interacting proteins in HEK293T cells**

| SYF2  KIF2C  KRT18  ZC3HAV1  DDX41  MCRIP2  RORA  FARP2  POU6F1  CCNA2  TRIM33  ACIN1  RAB14  PRPS2  SF3A3  UPF1  COPE  NOP58  RPL23  HNRNPA0  KIF2A  SLC25A11  PSMC3  MRPS18B  LAMTOR2  TMEM33  YLPM1  SRSF10  PEF1  THOC7  TFB2M  HDAC1  RPS11  FAM120A  SAFB  LSM12  SLC25A13  ATP5F1C  KHDRBS3  HSPA6  PDCD4  RPS3A  SLC25A5  ALKBH5  GPKOW  RBM4B  PURB  WDR6 | SF3B2  GEMIN5  PPP2R2A  RPL36  RBM45  CWC15  EMC8  EIF5B  RAB3D  EEF1G  CDKN2A  POLR2B  SF3A1  GEMIN6  RTCB  DNAJB1  IGF2BP2  EEF1D  MRPS22  STAU2  USP7  GFPT1  EPRS1  HBS1L  L2HGDH  ELAVL1  RBMS1  PRPSAP1  FUBP3  TRIR  NDUFB6  HDHD5  ASPH  APOC3  EMD  IARS1  YBX1  MCM5  RPL30  ASS1  GNL3  IGF2BP1  TRIM25  TCOF1  RRAS2  ZCCHC3  CAPRIN1  PSPC1 | U2AF1  GALK1  MT-CO2  RANBP2  ATP5PO  SRSF1  SYNCRIP  SF3B5  TOR1AIP1  LCLAT1  UQCRC2  LUC7L2  RPL10  EHD4  EMC4  PSMD1  SMARCE1  CPVL  RAB21  MRPS26  OLA1  MED14  SLC25A22  ILF3  ISY1  UBE2M  YBX3  STAT1  NCBP2  RER1  SUGP2  RPL29  RELA  HNRNPH2  PIP  BCAS2  AQR  PRPF40A  NUSAP1  WDR33  ELAVL2  NUP35  NUP160  RGPD5  C8orf33  AGO1  BAG6  NKRF | CHD5  RBM25  RANBP9  HSD17B12  NUP155  TBL2  ADAR  ARF6  DBT  THOC6  MSI1  MBNL1  PMPCB  AMOT  EEF1D  EEF1E1  EXOSC7  EXOSC2  MRPS2  RAB11FIP1  PTBP1  SNW1  MED8  LARP4B  HNRNPK  SAMM50  PHF5A  ZC3H11A  RHEB  XRN2  HNRNPH1  NDUFA13  RNPS1  DHCR7  MBOAT7  RPP25  SBDS  EFTUD2  HUWE1  ITPR3  PPIE  RBFOX2  CYC1  GINS3  CDC5L  MRPS23  CPSF1  NUP85 | MRPL53  GNA13  NCBP1  MAZ  RPL27  RMND5A  ITPA  AP3D1  POLR2C  PPP6C  RFC4  HNRNPK  KIF5B  PSMD4  CLTA  IRS4  NUP107  HNRNPF  HNRNPL  RUVBL1  RPS15  THOC1  DHX36  HAX1  BUD31  EEF1B2  MKRN2  RANGAP1  XAB2  PTCD3  REL  SRSF5  UPF3B  PABPC1  NUP133  RRP15  HSPA1B  EXOSC6  PRC1  PRPF19  SF3B6  DCAF7  TRA2A  SNRNP40  PSMD2  PRRC2A  AP3M2  MED9 | CPSF3  MED28  CCAR2  PRPF8  RUVBL2  CDC40  NDUFAF4  HDAC2  RBX1  MRPS14  HNRNPA3  MRPS9  NDUFB4  IBTK  OTOP2  MRPS35  HSPA8  RGPD3  ATAD3A  CPSF4  SMARCB1  GTF3C6  RPL10  PSMG3  PLRG1  SMN1  RNF126  HS2ST1  DYNC1LI1  DHX9  PSMD5  GEMIN2  AIMP2  PHF6  MRPS7  FIP1L1  DNAJA3  DNAJA2  THOC3  ZC3H13  HNRNPM  TMEM126A  MED27  BAIAP2L1  RAB9A  MRPL18  MOGS  STUB1 | STAU1  KDM1A  FHL3  CEP350  AKAP8L  LGALS3  MED6  TARS2  ATAD3B  GTF3C5  UTP14A  NUDT16L1  APOA1  CCNC  ERLIN1  POLD1  CCDC85C  EHD1  FAF2  NCOA1  GTF3C4  MAGEB2  EXOSC1  DNAJA1  SNRNP200  POLDIP3  POLR3A  CLTCL1  PRKRA  MYO1E  DDX20  MED15  GIGYF2  EXOSC4  CLTC  FAM120B  CRNKL1  RBM22  EXOSC3  SAFB2  EXOSC5  COQ8A  UBR5  HBB  MATR3  ZFR  HBA1 | KPNA1  C4A  SHROOM3  TSR1  SMARCC1  CLTB  WDR26  POLRMT  BAG4  MCCC2  ZNF622  TRAPPC3  DYNLL1  PPP1R12A  EXOSC10  PPIL4  PSIP1  MPDU1  TPM1  COBLL1  GIT2  SMU1  MRPL58  DCTN1  CALD1  CLPX  GET4  MED20  FYTTD1  THOC2  GOLGA3  CNIH4  SCCPDH  PABPC1  GPX4  BRI3BP  SEH1L  MED31  KDM6A  B2M  MED4  NCOA5  RFC1  DPY30  ECEL1  ARHGAP5  C7orf50  MED12 | WDR5  DNAJB6  EIF4B  FLOT2  CPS1  TMEM200B  MED17  HNRNPC  ZNF326  STAG2  UHMK1  BPIFB1  DDX54  NOP14  MED30  RALY  HRG  HNRNPC  MYH14  FHL2  IGHG1  MED1  HSP90AB4P  BAG5  BAG2  MED19  ASH2L  MED7  SMC1A  KMT2A  C3  GOLGA4  NKAP  LAMB1  STRBP  APOA4  CEP43  MED10  MED22  TFIP11  AKR1C3  RBM7  PAAF1  MED21  HP  ARHGEF7  TBC1D10C  NUDT15 | MED11  NCOA6  TRIM11  LZTS1  CHMP4B  RAD21  TFG  NAT2  PALM2AKAP2  MRE11  CCDC142  AFDN  RBBP5  MLF2  MAN2A2  PAGR1  CIZ1  ZCCHC8  TP53  KMT2D  AFDN  RAD50  PAXIP1  MTREX  TBL1XR1  PPM1F  ACAD9  DHX15  AK6  DAP3  QARS1  RPL21  SMC3  YTHDF1  RABGGTB  NBN  ESRP1  RFC3  PABPC4  SF3B1 |
| --- | --- | --- | --- | --- | --- | --- | --- | --- | --- |

**Supplementary Table 8. Putative binding sequences of POU6F1 in the RORA promoter region**

| **Name** | **Score** | **Relative Score** | **Start** | **End** | **Strand** | **Predicted binding sites** |
| --- | --- | --- | --- | --- | --- | --- |
| POU6F1  POU6F1  POU6F1  POU6F1  POU6F1  POU6F1  POU6F1 | 9.168  6.607  6.512  6.470  6.468  6.332  6.131 | 0.9065  0.8604  0.8587  0.858  0.8579  0.8555  0.8518 | 625  296  237  722  971  721  725 | 634  305  246  731  980  730  734 | -  -  -  -  -  +  - | ATAATCAGCA  GTAATGTTTA  CCAATGAGGC  ATGATGATTA  ATCATGACGT  TTAATCATCA  TTCATGATGA |
