# Supplementary material for: POU6F1 cooperates with RORA to suppress the proliferation of lung adenocarcinoma by downregulating HIF1A signaling pathway
Source: Cell Death Dis. 2022 May 3;13(5):427. doi: 10.1038/s41419-022-04857-y (PMC9065044; doi:10.1038/s41419-022-04857-y)
Supplement: Supplementary file 3 — Supplementary Figure and Table legends [file 41419_2022_4857_MOESM3_ESM.docx]

**Supplementary Figure and Table legends**

**Supplementary Fig. 1.** Homogeneity of variance analysis revealing the trend of POU6F1 expression level in the T stage of LUAD patients.

**Supplementary Fig. 2**

**A.** Oncomine database indicating the pan-cancer summary of POU6F1. The threshold *P*-value is limited to less than 0.001 and the fold change is 1.5. **B.** TIMER database showing the differential expression level of POU6F1 between tumor and adjacent normal tissues in all TCGA tumors. ACC, adrenocortical carcinoma; BLCA, bladder urothelial carcinoma; BRCA, breast invasive carcinoma, CESC, cervical squamous cell carcinoma and endocervical adenocarcinoma; CHOL, cholangio carcinoma; COAD, colon adenocarcinoma; DLBC, lymphoid neoplasm diffused large B-cell Lymphoma; ESCA, esophageal carcinoma; GBM, glioblastoma multiforme; HNSC, head and neck squamous cell carcinoma; KICH, kidney chromophobe; KIRC, kidney renal clear cell carcinoma; KIRP, kidney renal papillary cell carcinoma; LAML, acute myeloid leukemia; LGG, lower grade glioma; LIHC, liver hepatocellular carcinoma; LUAD, lung adenocarcinoma; LUSC, lung squamous cell carcinoma; MESO, mesothelioma; OV, ovarian serous cystadenocarcinoma; PAAD, pancreatic adenocarcinoma; PCPG pheochromocytoma and paraganglioma; PRAD, prostate adenocarcinoma; READ, rectum adenocarcinoma; SARC, sarcoma; SKCM, skin cutaneous melanoma; STAD, stomach adenocarcinoma; TGCT, testicular germ cell tumors; THCA, thyroid carcinoma; THYM, thymoma; UCEC, uterine corpus endometrial carcinoma; UCS, uterine carcinosarcoma; UVM, uveal melanoma. ∗*P* < 0.05; ∗∗*P* < 0.01; ∗∗∗*P* < 0.001.

**Supplementary Fig. 3**

**A-B.** Real-time qRT-PCR and western blotting assays indicating the expression of POU6F1 in A549 and NCI-H1299 cells transfected with CRISPRi-Scb, CRISPRi-POU6F1 #1, or CRISPRi-POU6F1 #2. **C.** Wound-healing assay revealing the migration ability of A549 and NCI-H1299 cells stably transfected with empty vector (mock) or POU6F1. Student’s t-test compared the difference in A. **P* < 0.05, ***P* < 0.01.

**Supplementary Fig. 4**

**A.** Kyoto Encyclopedia of Genes and Genomes (KEGG) classification analysis of differential expression genes (DEGs) in A549 cells stably transfected with POU6F1 compared with empty vector (mock). **B.** Gene Ontology (GO) analysis indicating the involved pathway of DEGs in A549 cells stably transfected with POU6F1 relative to mock.

**Supplementary Fig. 5**

**A-B.** Real-time qRT-PCR assay indicating the expression of ENO1, PDK1, and PRKCB in A549 and NCI-H1299 cells transfected with CRISPRi-Scb, CRISPR-POU6F1 #1, or CRISPR-POU6F1 #2. **C.** Real-time qRT-PCR assay showing the expression of HIF1A, ENO1, PDK1, and PRKCB in NCI-H1299 cells transfected with empty vector (mock) or POU6F1. **D.** Dual-luciferase assay showing relative activity of ENO1, PDK1, and PRKCB promoter in HEK293T cells transfected with mock or POU6F1. Student’s t-test and ANOVA compared the difference in A–D. **P* < 0.05, ***P* < 0.01.

**Supplementary Fig. 6**

**A.** Immunofluorescence assay revealing the location of POU6F1 in A549 and NCI-H1299 cells transfected with empty vector (pCMV-HA) or pCMV-HA-POU6F1. Scale bar: 10µm. **B.** Western blotting assay showing the cytoplasmic and nuclear expression of POU6F1 in A549 and NCI-H1299 cells transfected with mock or POU6F1. **C.** Western blotting and real-time qRT-PCR assays showing the expression of RORA in A549, NCI-H1299, H1975, and SPC compared with HBE. **D-E.** Real-time qRT-PCR assay indicating the expression of RORA and POU6F1 in A549 and H1975 cells transfected with mock, RORA, scramble shRNA (sh-Scb), sh-RORA #1, or sh-RORA #2. **F**. Western blotting showing the expression of RORA and POU6F1 in H1975 cells transfected with mock, RORA, sh-Scb, sh-RORA #1, or sh-RORA #2. **G.** Ubiquitylation assay showing the ubiquitination level of RORA in HEK293T cells transfected with Flag-Ub, and those treated with DMSO or MG132 (5 μmol/l). **H-I.** Public dataset analysis showing the correlation between POU6F1 and RORA using GEPIA and TCGA database. Student’s t-test and ANOVA compared the difference in C-E. Pearson’s correlation coefficient analysis compared the correlation between POU6F1 and RORA in H and I. **P* < 0.05, ***P* < 0.01.

**Supplementary Fig. 7**

**A.** Mining public dataset (TCGA-LUAD) showing the differential level of RORA in LUAD patients with different statuses of death, tumor stage (IV vs. Ia), and metastasis. **B.** Expression level of RORA in unpaired (upper panel) or paired (lower panel) LUAD tissues compared with normal tissues derived from TCGA-LUAD dataset. **C.** Kaplan-Meier curves revealing overall survival (OS) and first progression (FP) of LUAD patients with high or low levels of RORA. **D.** Proteins were extracted from LUAD tissues and adjacent normal tissues and assessed by western blotting assay (D, left panel). The relative protein expression (D, right panel) of RORA was normalized to that of β-actin using ImageJ software. **E-F.** Dual-luciferase assay indicating the relative promoter activity of HIF1A, ENO1, PDK1, and PRKCB in A549 cells transfected with empty vector (mock) or RORA. Log-rank test for survival comparison in C. Student’s t-test and ANOVA compared the difference in D-F **P* < 0.05, ***P* < 0.01.

**Supplementary Fig. 8**

**A-B.** Real-time qRT-PCR assay indicating the expression of POU6F1, RORA, HIF1A, ENO1, PDK1, and PRKCB in NCI-H1299 cells transfected with empty vector (mock), RORA, CRISPRi-Scb, or CRISPRi-POU6F1 #1. **C-D.** Representative images (left panel) and quantification (right panel) of soft-agar (C) and transwell (D) assays indicating the growth and invasion of NCI-H1299 cells transfected with mock, RORA, CRISPRi-Scb, or CRISPRi-POU6F1 #1. Student’s t-test and ANOVA compared the difference in A–D. **P* < 0.05, ***P* < 0.01.

**Supplementary Fig. 9**

**A-C.** The expression (left panel), overall survival (OS, middle panel), and first progression (FP, right panel) of ENO1 (A), PDK1 (B), and PRKCB (C) in LUAD tissues compared with normal tissues. **D.** Expression levels of ENO1 (upper panel), PDK1 (middle panel), and PRKCB (lower panel) in LUAD tissues relative to matched normal tissues.

**Supplementary Tables**

**Supplementary Table 1. Transcription factors details list that was associated with the death of LUAD patients**

The screened transcription factors (TFs) closely associated with death, derived from a public LUAD dataset of 515 cases.

**Supplementary Table 2. Transcription factors details list that was associated with tumor stage of LUAD patients**

The screened transcription factors (TFs) closely associated with tumor stage, derived from a public LUAD dataset of 515 cases.

**Supplementary Table 3. Transcription factors details list that was associated with metastasis of LUAD patients**

The screened transcription factors (TFs) closely associated with metastasis, derived from a public LUAD dataset of 515 cases.

**Supplementary Table 4. Primer sets used for qRT-PCR and ChIP**

POU6F1, POU domain, class 6, transcription factor 1; ACTB, beta-actin; GAPDH, glyceraldehyde 3-phosphate dehydrogenase; RORA, retinoid-related orphan receptor alpha; ENO1, enolase 1; ENO2, enolase 2; PDK1, pyruvate dehydrogenase kinase 1; PRKCB, protein kinase C beta; and HIF1A, hypoxia inducible factor 1, alpha subunit; ChIP, chromatin immunoprecipitation.

**Supplementary Table 5. Oligonucleotide sets used for constructs and short hairpin RNAs**

**Supplementary Table 6. Correlation between POU6F1 expression and clinical parameters in LUAD patients.**

**Supplementary Table 7. Mass spectrometry (MS) analysis of POU6F1-interacting proteins**

**Supplementary Table 8. Putative binding sequences of POU6F1 in the RORA promoter region**
