# Supplementary figures and images for: POU6F1 cooperates with RORA to suppress the proliferation of lung adenocarcinoma by downregulating HIF1A signaling pathway
Source: Cell Death Dis. 2022 May 3;13(5):427. doi: 10.1038/s41419-022-04857-y (PMC9065044; doi:10.1038/s41419-022-04857-y)

**Main Figures**

**
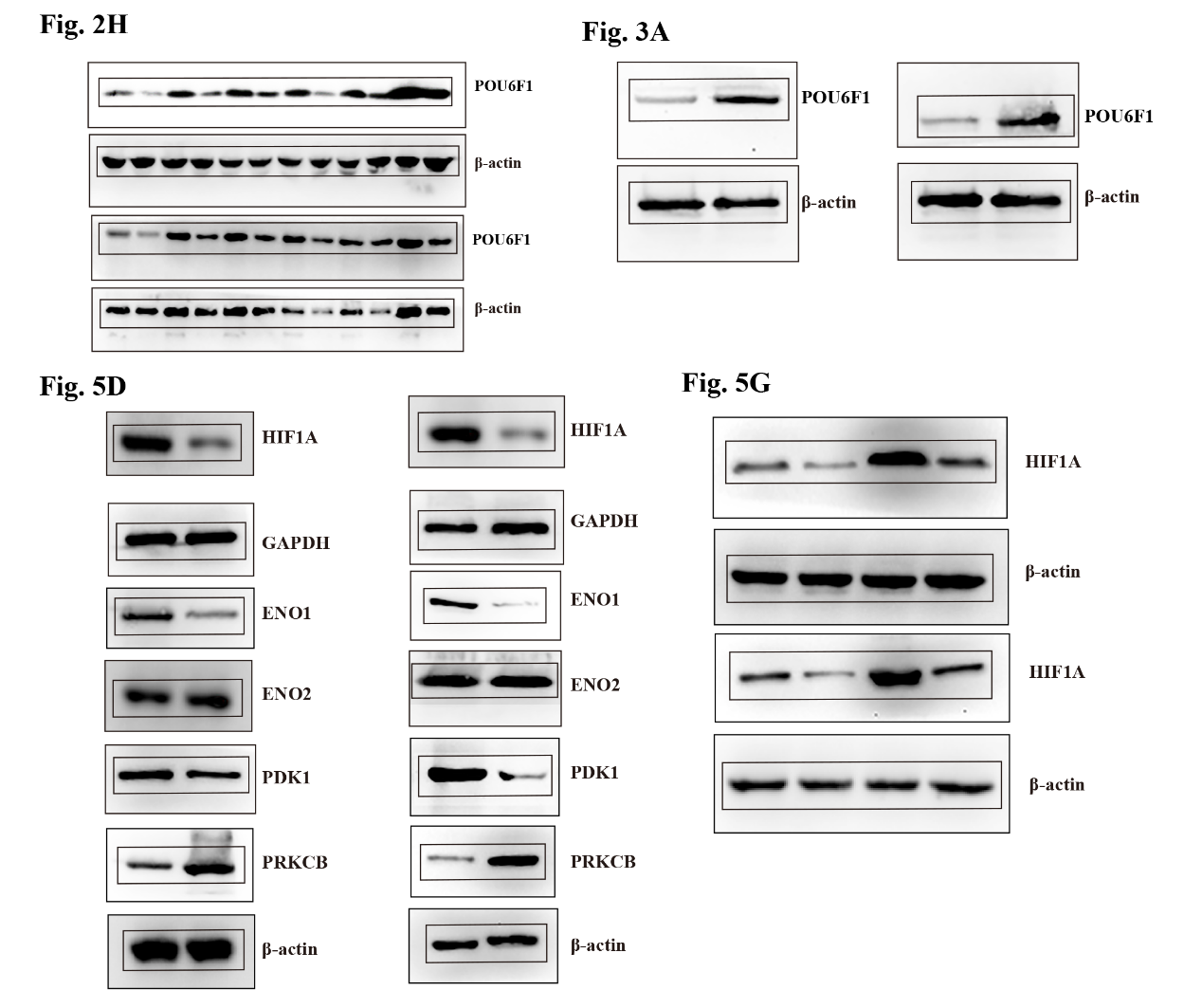
**

**
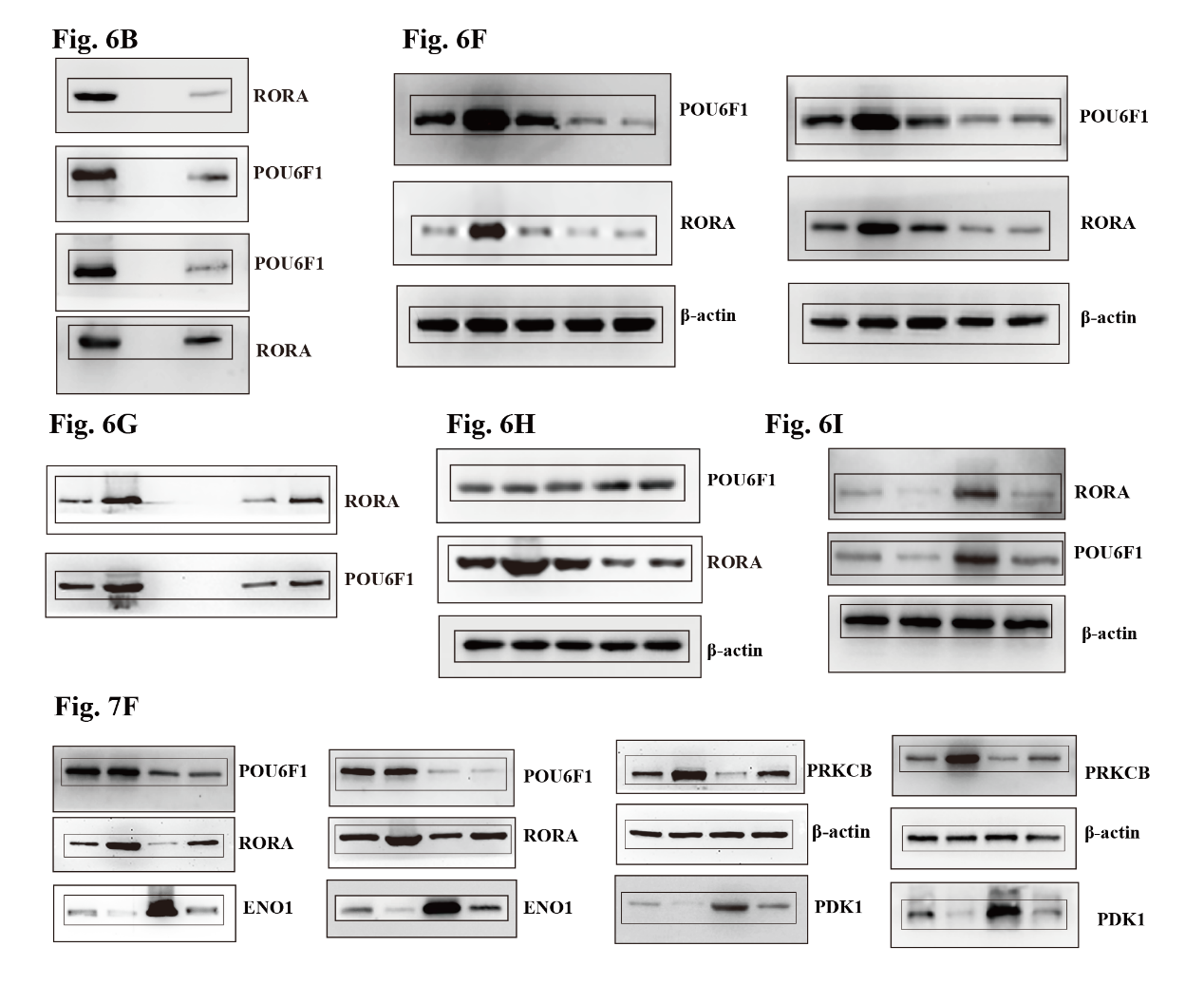
**

**Supplemenatry Figures**

**
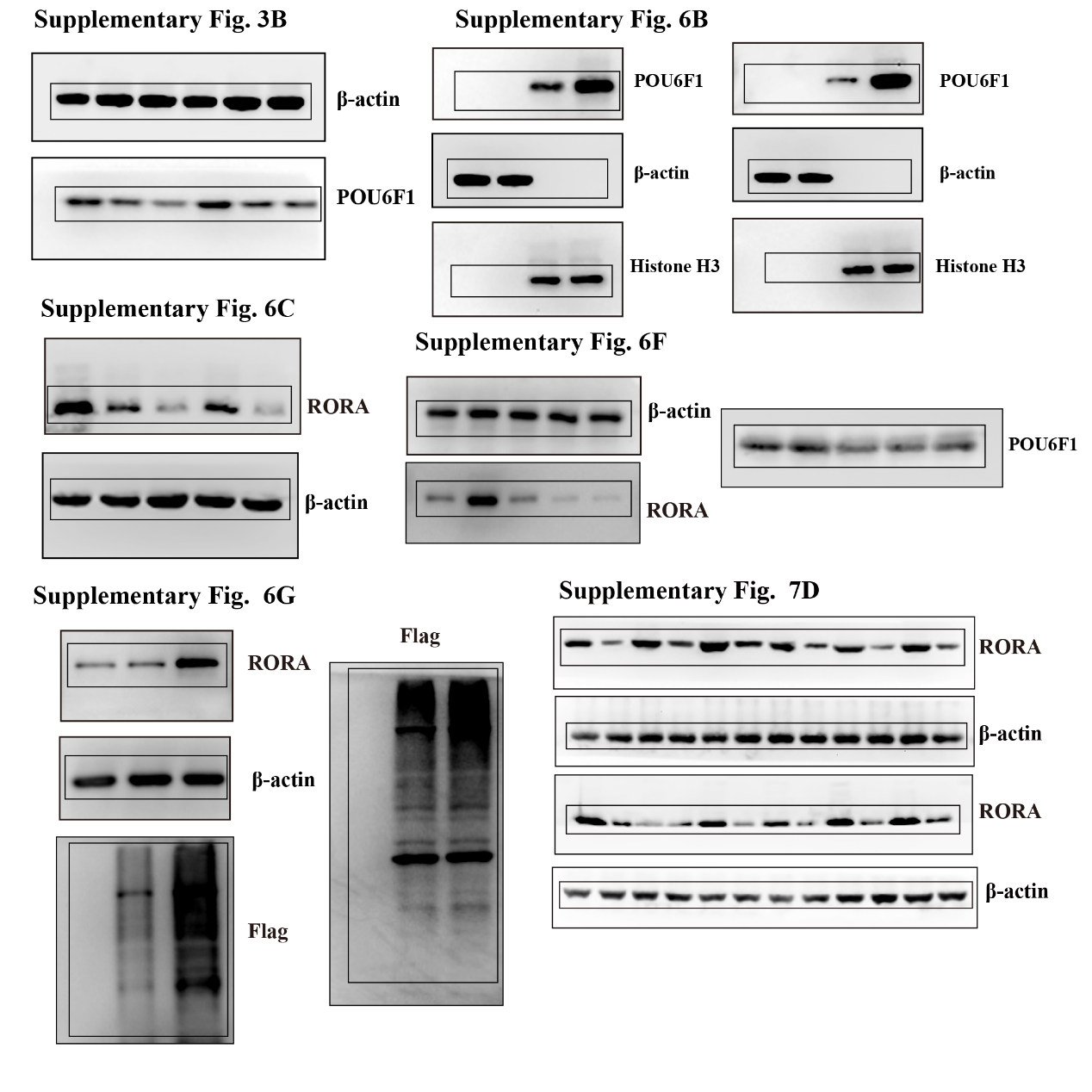
**

Supplement: Supplementary file 4 — POU6F1 WB [file 41419_2022_4857_MOESM4_ESM.docx]

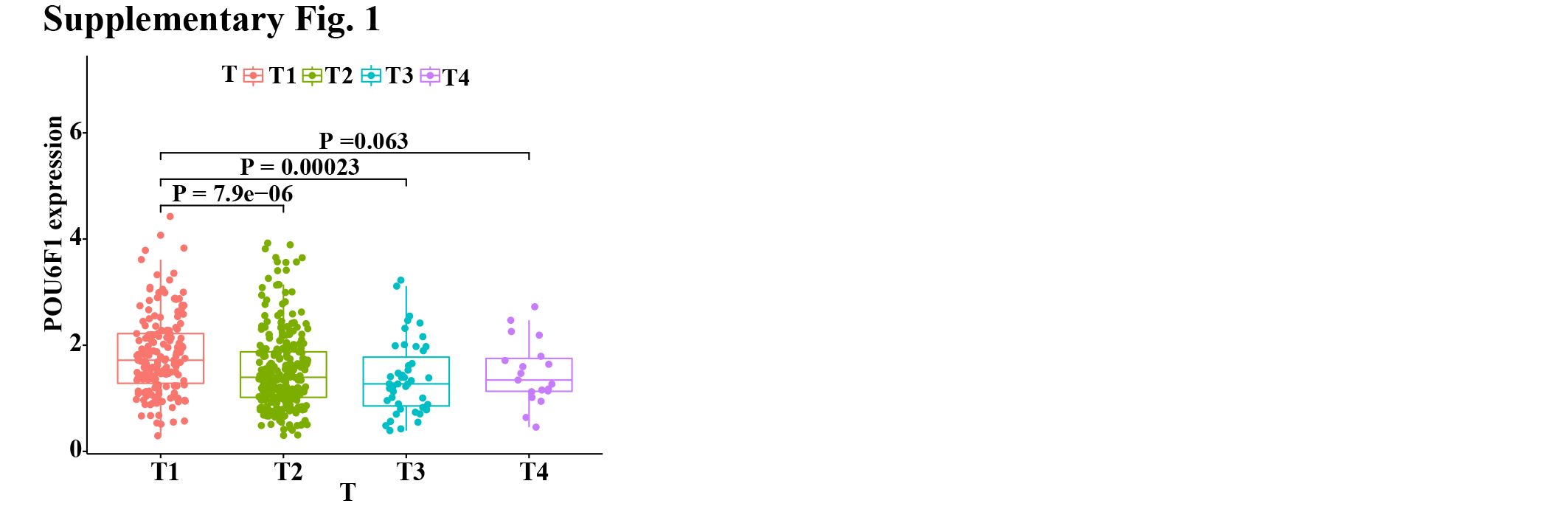

Supplement: Supplementary file 5 — Supplementary Figure 1 [file 41419_2022_4857_MOESM5_ESM.tif]

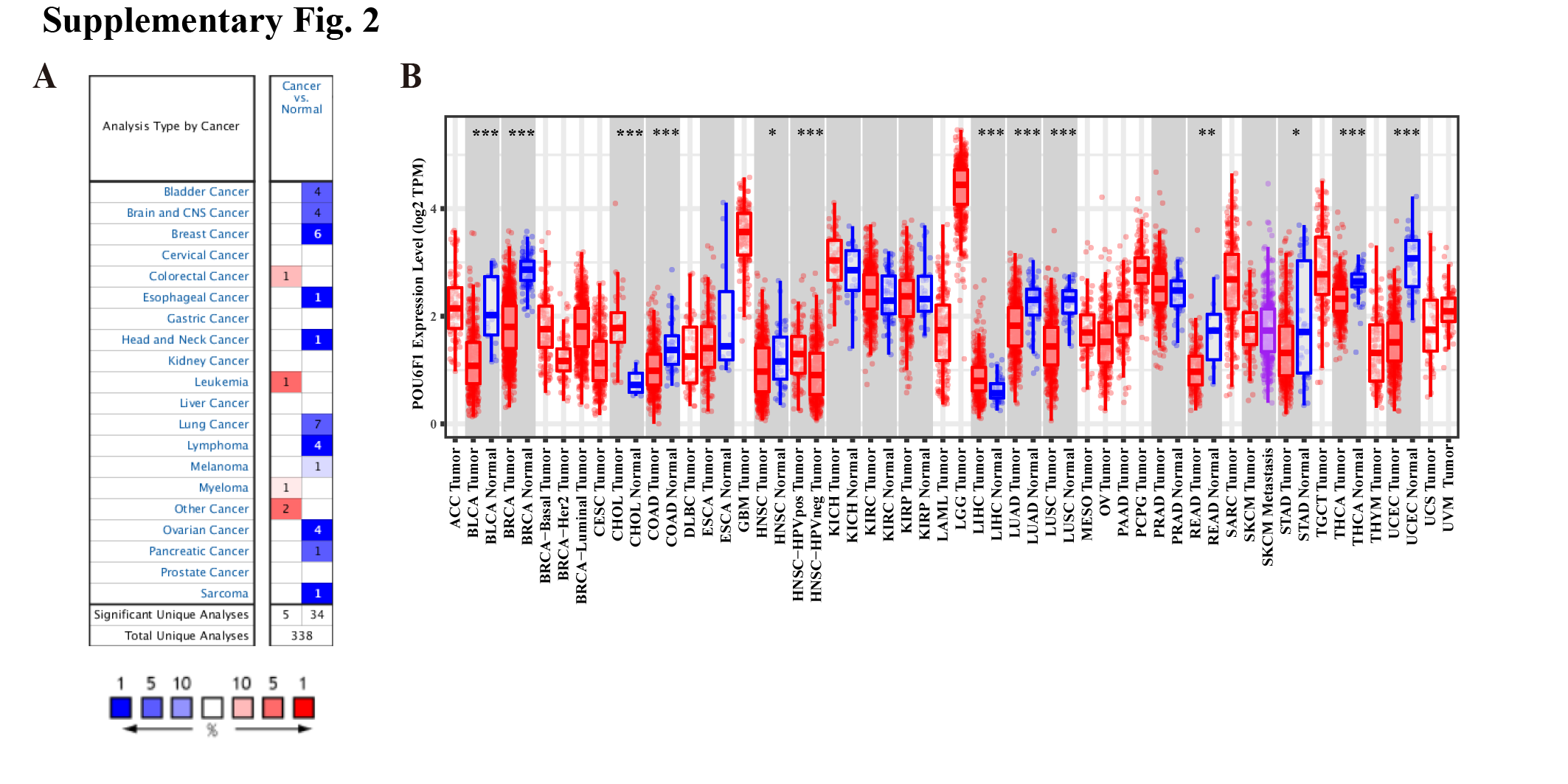

Supplement: Supplementary file 6 — Supplementary Figure 2 [file 41419_2022_4857_MOESM6_ESM.tif]

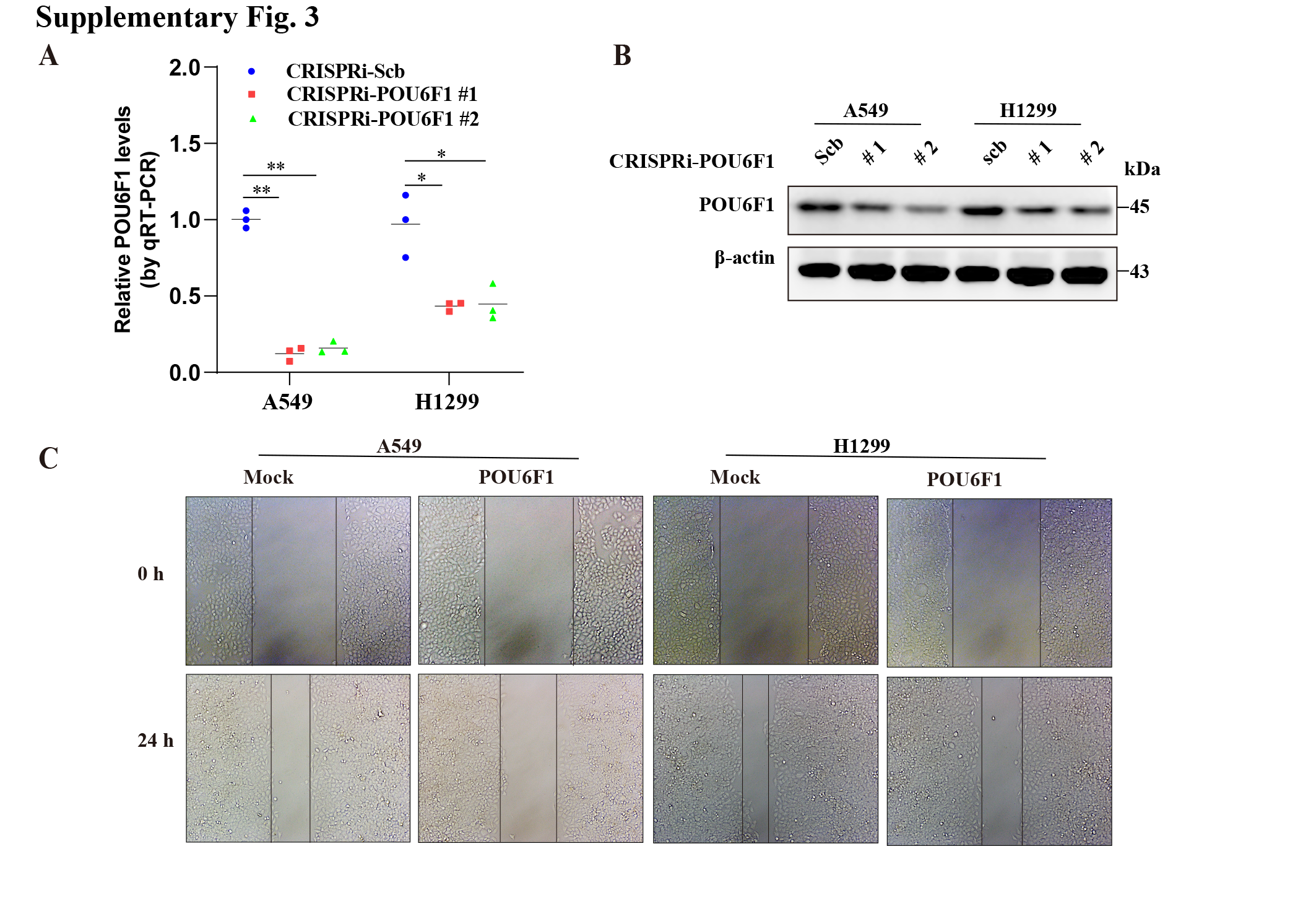

Supplement: Supplementary file 7 — Supplementary Figure 3 [file 41419_2022_4857_MOESM7_ESM.tif]

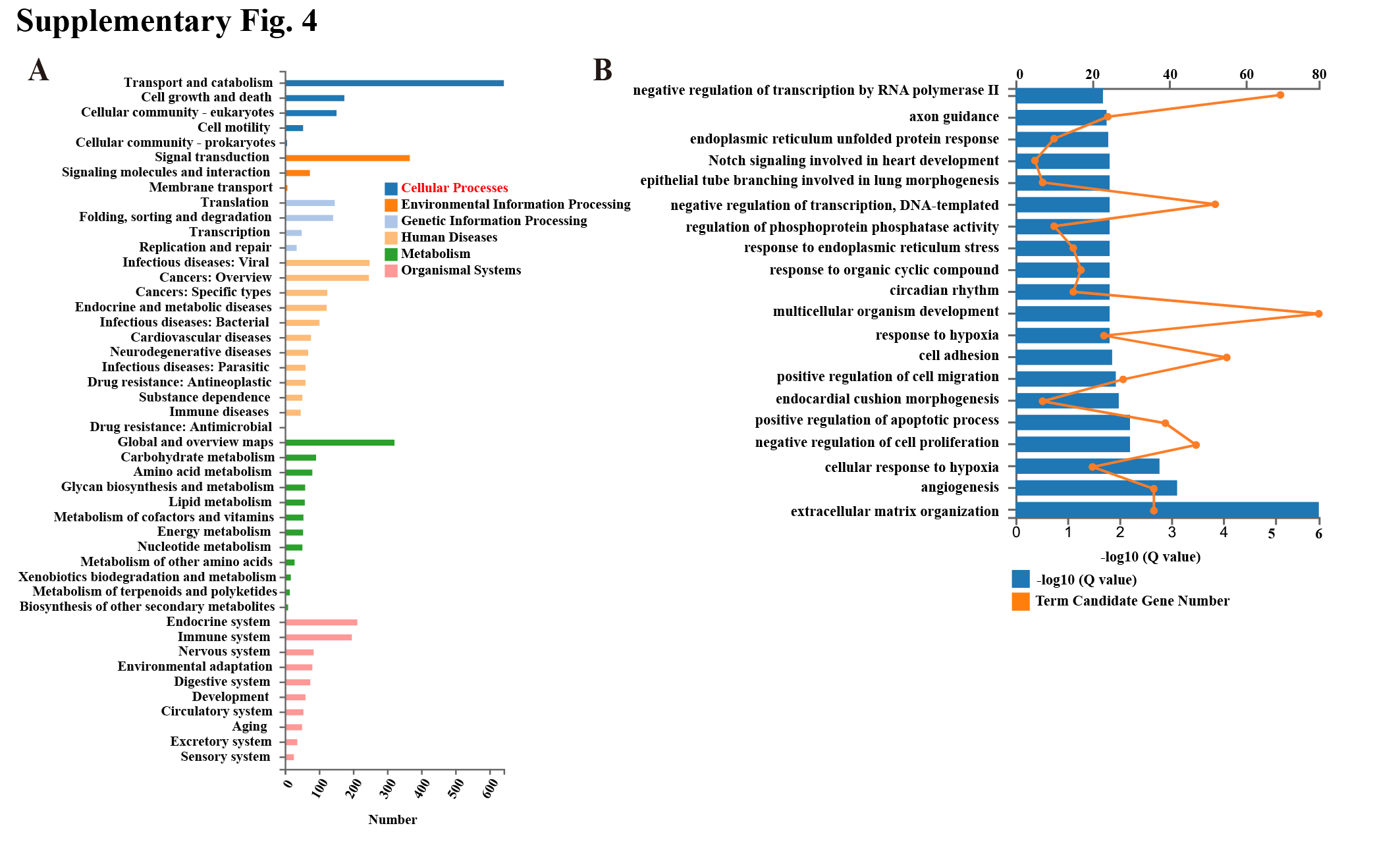

Supplement: Supplementary file 8 — Supplementary Figure 4 [file 41419_2022_4857_MOESM8_ESM.tif]

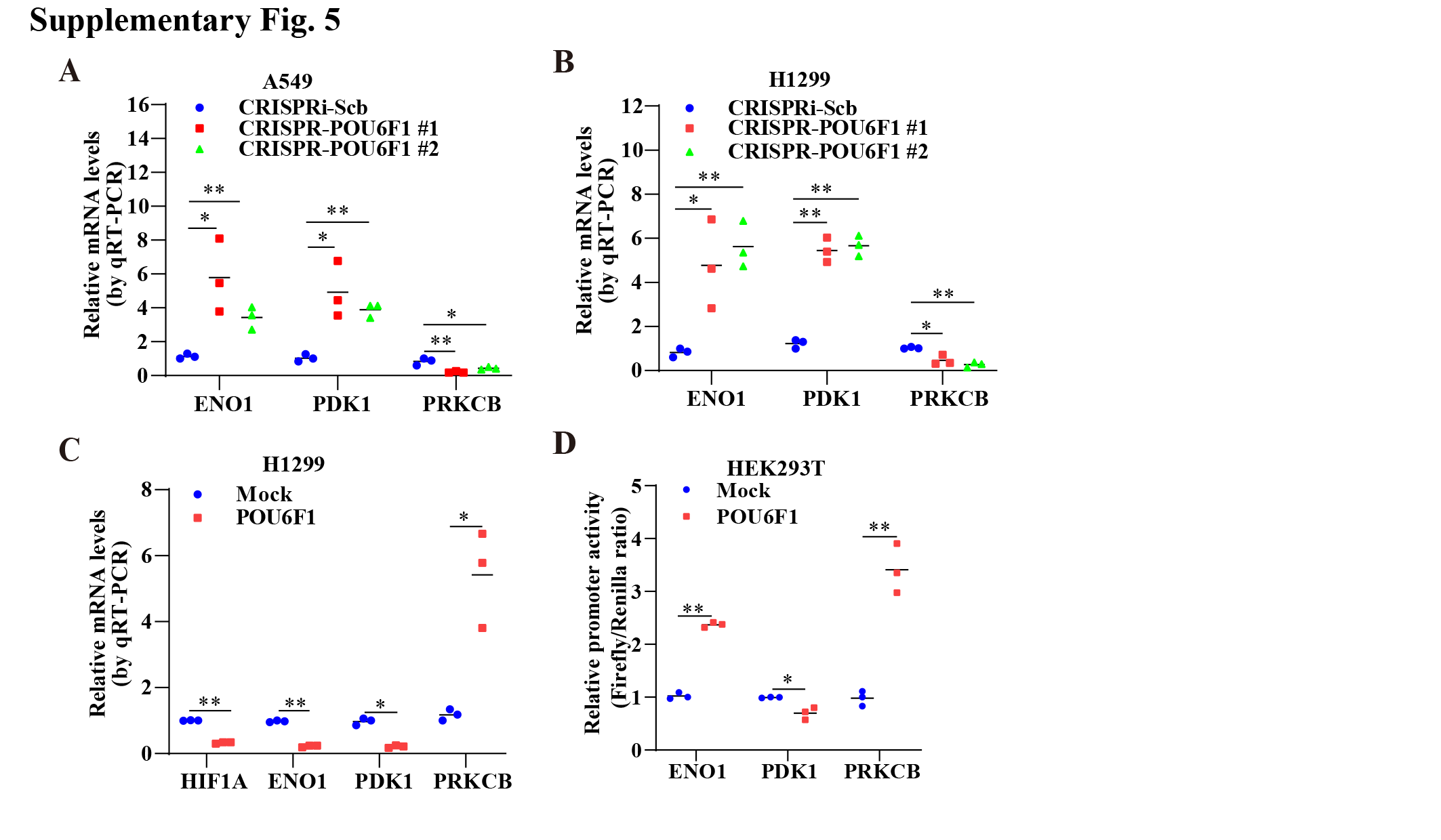

Supplement: Supplementary file 9 — Supplementary Figure 5 [file 41419_2022_4857_MOESM9_ESM.tif]

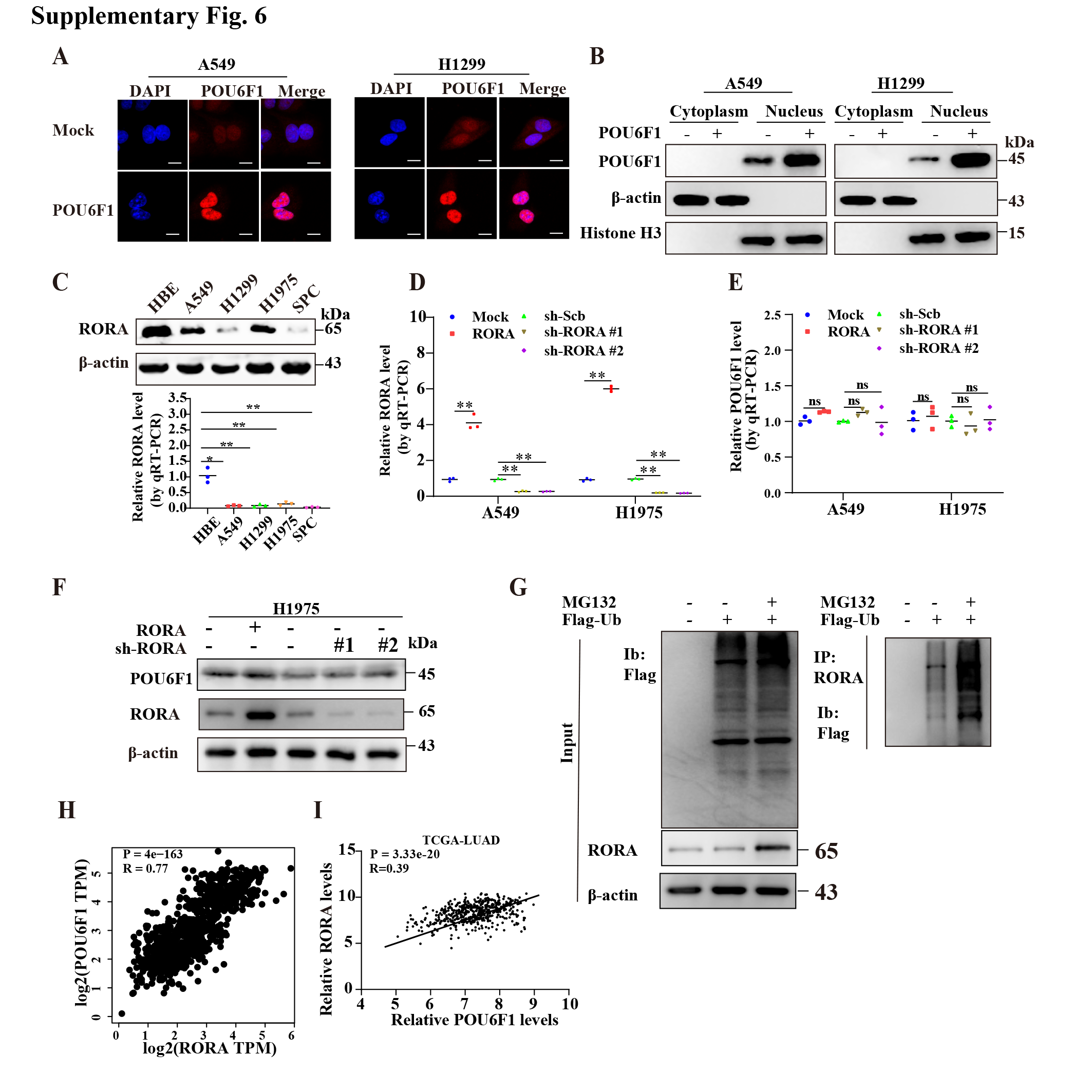

Supplement: Supplementary file 10 — Supplementary Figure 6 [file 41419_2022_4857_MOESM10_ESM.tif]

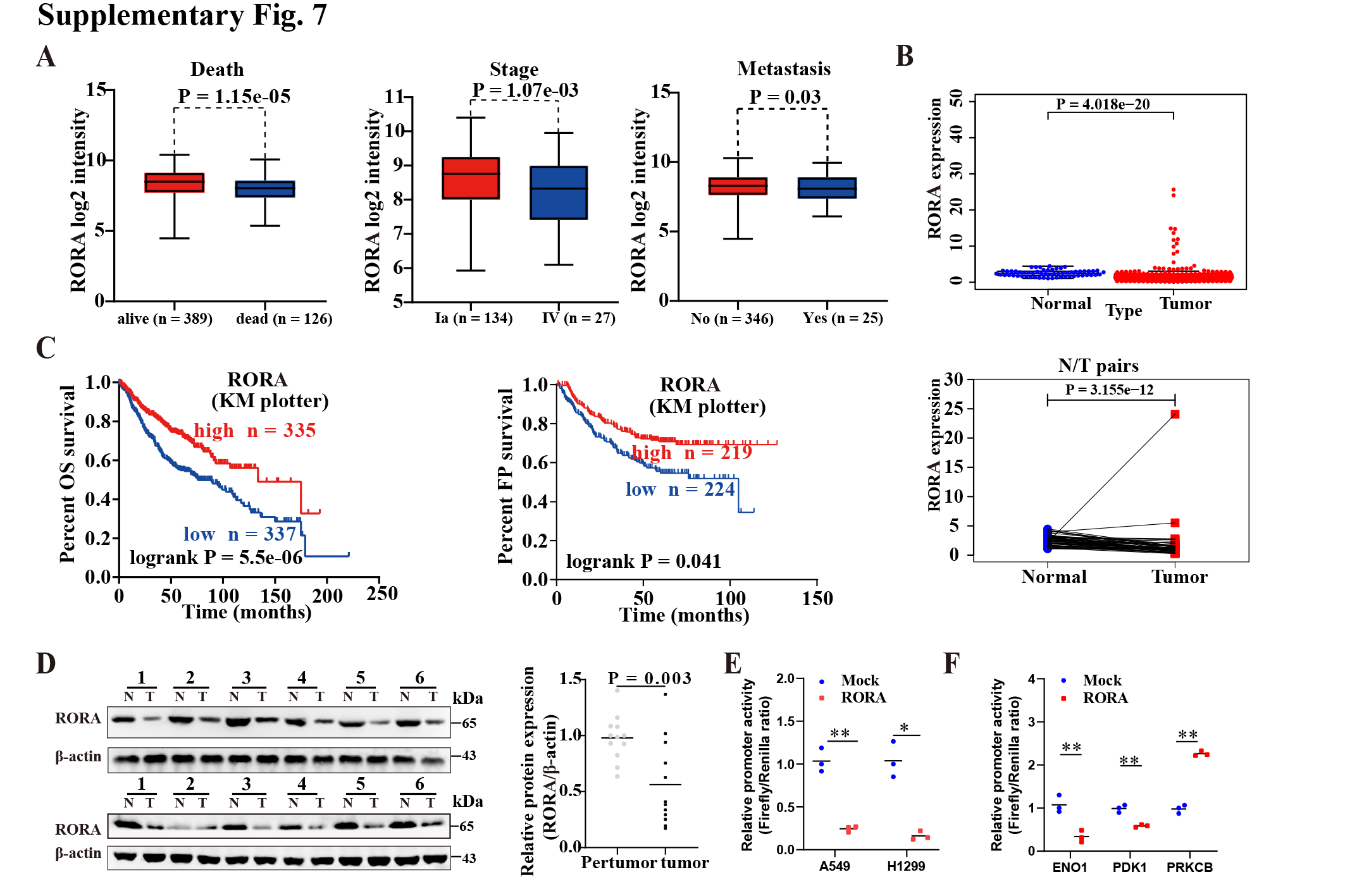

Supplement: Supplementary file 11 — Supplementary Figure 7 [file 41419_2022_4857_MOESM11_ESM.tif]

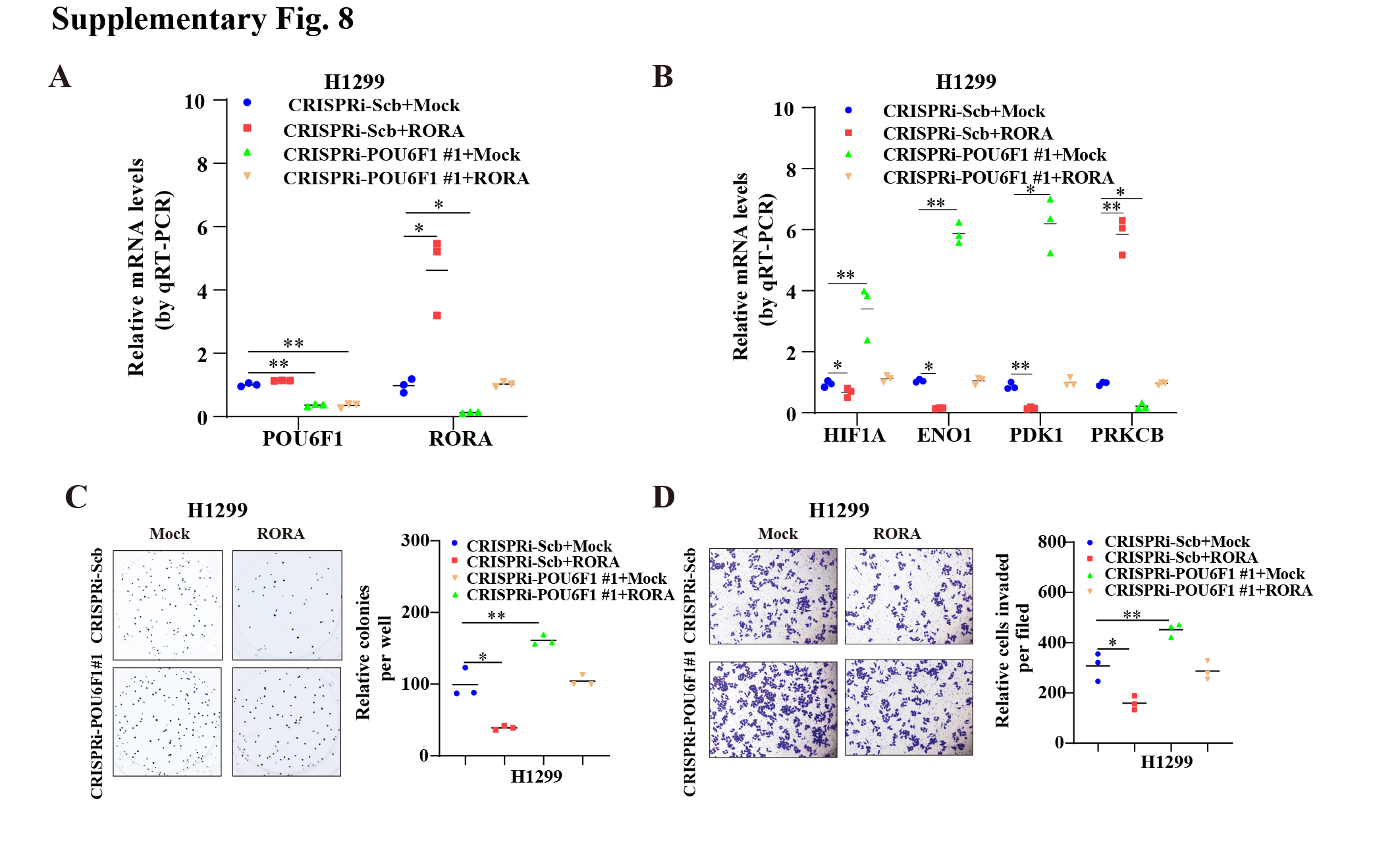

Supplement: Supplementary file 12 — Supplementary Figure 8 [file 41419_2022_4857_MOESM12_ESM.tif]

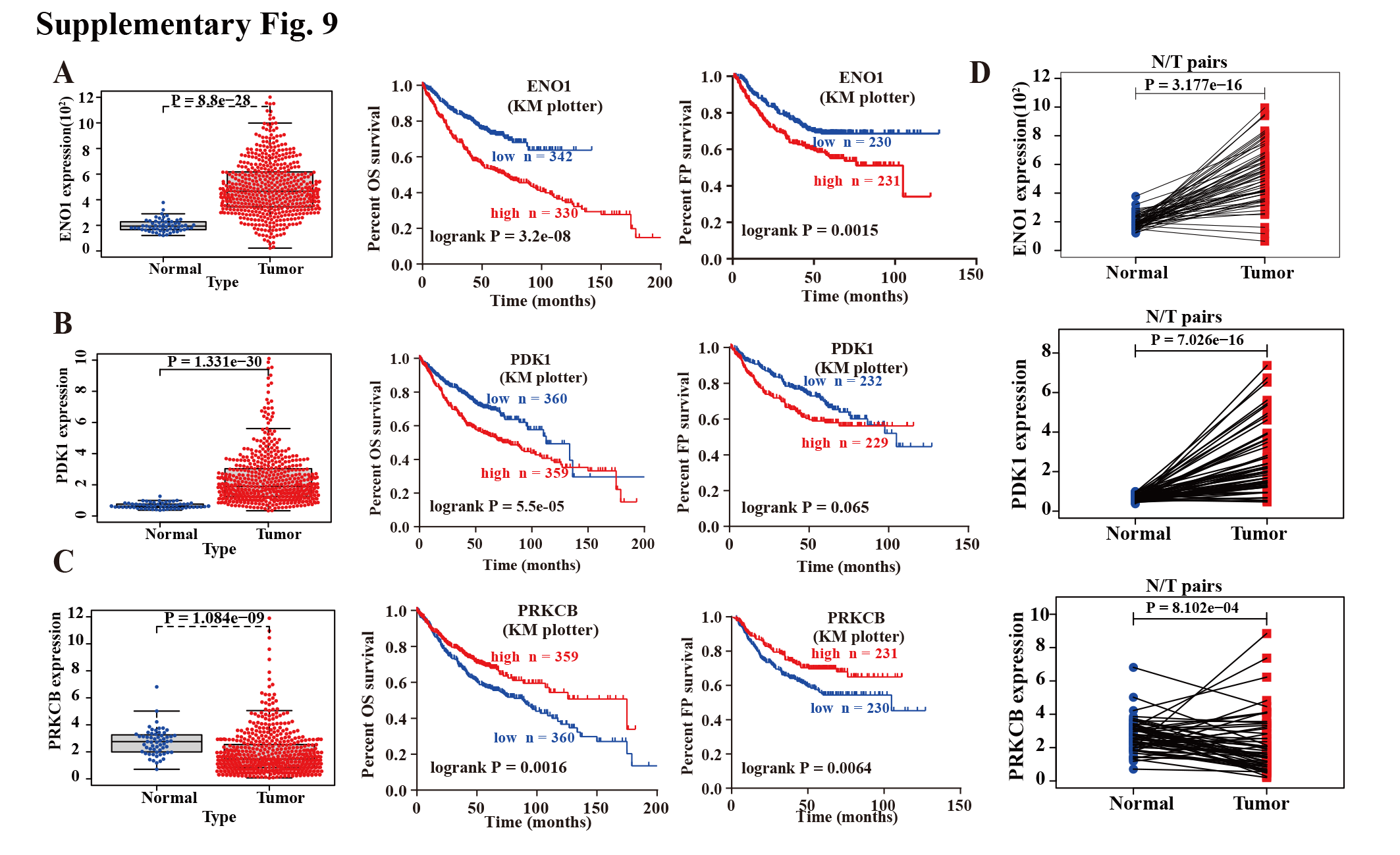

Supplement: Supplementary file 13 — Supplementary Figure 9 [file 41419_2022_4857_MOESM13_ESM.tif]
