# Supplementary material for: POU6F1 cooperates with RORA to suppress the proliferation of lung adenocarcinoma by downregulating HIF1A signaling pathway
Source: Cell Death Dis. 2022 May 3;13(5):427. doi: 10.1038/s41419-022-04857-y (PMC9065044; doi:10.1038/s41419-022-04857-y)
Supplement: Supplementary file 14 — Supplementary Table 1 [file 41419_2022_4857_MOESM14_ESM.docx]

**Supplementary Table 1 Transcription factors details list that were associated with the death of LUAD patients**

| 1) CDX4 | 31) FOXM1 | 61) NFATC1 | 91) FOXL2 |
| --- | --- | --- | --- |
| 2) CRX | 32) RFX5 | 62) ZNF189 | 92) STAT3 |
| 3) HSFX1 | 33) IRX6 | 63) ERG | 93) PHF1 |
| 4) HMGA1 | 34) RFXAP | 64) POU3F1 | 94) TEF |
| 5) BTG2 | 35) ARNTL | 65) HCLS1 | 95) ZNF41 |
| 6) ZNF154 | 36) HLF | 66) NFATC2 | 96) FOXN3 |
| 7) ZNF397 | 37) IRF4 | 67) CREBZF | 97) PBX4 |
| 8) RORA | 38) ZNF396 | 68) IRX4 | 98) ZHX3 |
| 9) FOSL1 | 39) YEATS4 | 69) KLF2 | 99) LHX1 |
| 10) ZNF449 | 40) ELF5 | 70) TFEB | 100) XBP1 |
| 11) YBX1 | 41) IRX5 | 71) ATRX | 101) NR2E3 |
| 12) DMTF1 | 42) MYBL2 | 72) HSF5 | 102) HDAC2 |
| 13) NR0B2 | 43) FMNL2 | 73) EGR3 | 103) EOMES |
| 14) CBFA2T3 | 44) HSF1 | 74) ZNF81 | 104) E2F7 |
| 15) IKZF4 | 45) CCRN4L | 75) PKNOX2 | 105) KLF15 |
| 16) TEAD4 | 46) EHF | 76) UBP1 |  |
| 17) POU6F1 | 47) PRDM16 | 77) TRIM22 |  |
| 18) ZNF80 | 48) PHF5A | 78) GAS7 |  |
| 19) VAX1 | 49) NR3C2 | 79) SETD2 |  |
| 20) REL | 50) ARID4A | 80) RUNX1T1 |  |
| 21) TGIF1 | 51) TRIM28 | 81) ZNF70 |  |
| 22) ZNF33A | 52) ZNF19 | 82) EGR2 |  |
| 23) ENO1 | 53) ZNF83 | 83) IRX2 |  |
| 24) ZNF169 | 54) PRDM2 | 84) NFIX |  |
| 25) VENTX | 55) SOX6 | 85) ZNF500 |  |
| 26) ZNF483 | 56) BTAF1 | 86) UHRF1 |  |
| 27) ZNF211 | 57) PFDN1 | 87) IRF8 |  |
| 28) PITX3 | 58) NR2C2 | 88) FOXO4 |  |
| 29) TFAM | 59) ZNF91 | 89) PEG3 |  |
| 30) PA2G4 | 60) MLLT10 | 90) ZSCAN22 |  |

The screened transcription factors (TFs) closely associated with death, derived from a public LUAD dataset of 515 cases.
