# Supplementary material for: POU6F1 cooperates with RORA to suppress the proliferation of lung adenocarcinoma by downregulating HIF1A signaling pathway
Source: Cell Death Dis. 2022 May 3;13(5):427. doi: 10.1038/s41419-022-04857-y (PMC9065044; doi:10.1038/s41419-022-04857-y)
Supplement: Supplementary file 15 — Supplementary Table 2 [file 41419_2022_4857_MOESM15_ESM.docx]

**Supplementary Table 2 Transcription factors details list that were associated with tumor stage of LUAD patients**

| 1) ARGFX | 11) IRF8 | 21) RUNX3 |
| --- | --- | --- |
| 2) ATF1 | 12) IRX4 | 22) SMC3 |
| 3) ATF2 | 13) MYF5 | 23) SPIB |
| 4) CDX4 | 14) NEUROG1 | 24) TAF5 |
| 5) CRX | 15) NFIB | 25) TBX21 |
| 6) EOMES | 16) NFXL1 | 26) VAV1 |
| 7) FOXP3 | 17) NKX2-2 | 27) VAX1 |
| 8) HCLS1 | 18) PBX4 | 28) ZNF444 |
| 9) HSFX1 | 19) POU2F2 | 29) ZNF80 |
| 10) IRF4 | 20) POU6F1 |  |

The screened transcription factors (TFs) closely associated with tumor stage, derived from a public LUAD dataset of 515 cases
