# Supplementary material for: POU6F1 cooperates with RORA to suppress the proliferation of lung adenocarcinoma by downregulating HIF1A signaling pathway
Source: Cell Death Dis. 2022 May 3;13(5):427. doi: 10.1038/s41419-022-04857-y (PMC9065044; doi:10.1038/s41419-022-04857-y)
Supplement: Supplementary file 16 — Supplementary Table 3 [file 41419_2022_4857_MOESM16_ESM.docx]

**Supplementary Table 3 Transcription factors details list that were associated with metastasis of LUAD patients**

| 1) ARID4A | 31) HIRA | 61) SCRT1 | 91) ZNF83 |
| --- | --- | --- | --- |
| 2) ARNT2 | 32) HMGA1 | 62) SETD2 | 92) ZNFX1 |
| 3) ATF1 | 33) HMX3 | 63) SMARCA4 | 93) ZSCAN18 |
| 4) BTAF1 | 34) HOXB3 | 64) ST18 | 94) ZSCAN22 |
| 5) CBFA2T2 | 35) HSFX1 | 65) SUPT4H1 |  |
| 6) CBL | 36) LZTR1 | 66) TAF13 |  |
| 7) CDX4 | 37) MGA | 67) TARDBP |  |
| 8) CITED1 | 38) MLX | 68) TBX19 |  |
| 9) CNBP | 39) MTF1 | 69) TEAD1 |  |
| 10) CNOT8 | 40) MZF1 | 70) TEF |  |
| 11) CREB3 | 41) NFAT5 | 71) TFAM |  |
| 12) CREBZF | 42) NFIA | 72) TULP4 |  |
| 13) CREM | 43) NFIL3 | 73) YEATS4 |  |
| 14) CRX | 44) NME2 | 74) ZHX3 |  |
| 15) DLX1 | 45) NR2C2 | 75) ZKSCAN1 |  |
| 16) DMTF1 | 46) NR2E3 | 76) ZNF134 |  |
| 17) E2F5 | 47) PAX6 | 77) ZNF135 |  |
| 18) ELF5 | 48) PEG3 | 78) ZNF154 |  |
| 19) ESR2 | 49) PFDN1 | 89) ZNF175 |  |
| 20) ETV6 | 50) PHF5A | 80) ZNF18 |  |
| 21) FMNL2 | 51) PITX2 | 81) ZNF202 |  |
| 22) FOXC1 | 52) POU3F1 | 82) ZNF207 |  |
| 23) FOXD1 | 53) POU6F1 | 83) ZNF211 |  |
| 24) FOXI1 | 54) PRDM2 | 84) ZNF256 |  |
| 25) FOXJ3 | 55) PURB | 85) ZNF274 |  |
| 26) FOXK1 | 56) RB1 | 86) ZNF33A |  |
| 27) FOXN3 | 57) REL | 87) ZNF37A |  |
| 28) GATAD2B | 58) RERE | 88) ZNF445 |  |
| 29) GTF2A2 | 59) RUNX1 | 89) ZNF70 |  |
| 30) HAND2 | 60) SCMH1 | 90) ZNF81 |  |

The screened transcription factors (TFs) closely associated with metastasis, derived from a public LUAD dataset of 515 cases
