# Supplementary material for: POU6F1 cooperates with RORA to suppress the proliferation of lung adenocarcinoma by downregulating HIF1A signaling pathway
Source: Cell Death Dis. 2022 May 3;13(5):427. doi: 10.1038/s41419-022-04857-y (PMC9065044; doi:10.1038/s41419-022-04857-y)
Supplement: Supplementary file 17 — Supplementary Table 4 [file 41419_2022_4857_MOESM17_ESM.docx]

**Supplementary Table 4. Primer sets used for qRT-PCR and ChIP**

| **Primer set** | **Primers** | **Sequence** | **Product size (bp)** | **Application** |
| --- | --- | --- | --- | --- |
| POU6F1 | Forward | 5’-TGCTCAGGGACAGGTTATTGG-3' | 201 | qRT-PCR |
|  | Reverse | 5’-GACTCAGGTGTGCTTGGCTTC-3' |  |  |
| GAPDH | Forward | 5’-ATGTCCCAGCTCTCCTCCACC-3' | 160 | qRT-PCR |
|  | Reverse | 5’-CTACATTCGGGAGGGCGGGCT-3' |  |  |
| β-actin | Forward | 5'-TGCCCATCTACGAGGGGTATG-3’ | 156 | qRT-PCR |
|  | Reverse | 5'-TCTCCTTAATGTCACGCACGATTT-3’ |  |  |
| RORA | Forward | 5'-GATGCTTTTGTTCTTACTGGCG-3’ | 220 | qRT-PCR |
|  | Reverse | 5'-CATTGCTTTGCTGACTTCTCCT-3’ |  |  |
| ENO1 | Forward | 5'-GCCTCCTGCTCAAAGTCAAC-3’ | 102 | qRT-PCR |
|  | Reverse | 5'-AACGATGAGACACCATGACG-3’ |  |  |
| ENO2 | Forward | 5'-GTCATCAAGGACAAATACGGCAAG-3’ | 174 | qRT-PCR |
|  | Reverse | 5'-ATAAAACTCTGAGGCAGCAACATC-3’ |  |  |
| PDK1 | Forward | 5'-TGGTTTTGGTTATGGATTGCCC-3’ | 194 | qRT-PCR |
|  | Reverse | 5'-CAGCCTCGTGGTTGGTGTTGTA-3’ |  |  |
| PRKCB | Forward | 5'- AGCCCCACGTTTTGTGACC-3’ | 117 | qRT-PCR |
|  | Reverse | 5'-GCTGGGAACATTCATCACGC-3’ |  |  |
| HIF1A | Forward | GCCTTGGATGGTTTTGTTATGGTT | 216 | qRT-PCR |
|  | Reverse | GCTTCGCTGTGTGTTTTGTTCTTT |  |  |
| ENO1 (-991/-853) | Forward | TCCAACTCCTTCCGTATTCCAC | 284 | ChIP |
|  | Reverse | TTACATCCCTCTCATTCCCATC |  |  |
| PDK1 (-922/-680) | Forward | AACCGCCAAAAACCGTTTTCTC | 242 | ChIP |
|  | Reverse | AGCCTCCGTCTCTTTACCTGAA |  |  |
| PRKCB (-1234/-1034) | Forward | CATAGGGTTGTTGCGGGGGCTA | 170 | ChIP |
|  | Reverse | TATTGGTTGATTGAGTGAGGGC |  |  |

POU6F1, POU domain, class 6, transcription factor 1; ACTB, β-actin; GAPDH, glyceraldehyde 3-phosphate dehydrogenase; RORA, retinoid-related orphan receptor alpha; ENO1, enolase 1; ENO2, enolase 2; PDK1, pyruvate dehydrogenase kinase 1; PRKCB, protein kinase C beta; HIF1A, hypoxia-inducible factor 1, alpha subunit; ChIP, chromatin immunoprecipitation.
