# Supplementary material for: POU6F1 cooperates with RORA to suppress the proliferation of lung adenocarcinoma by downregulating HIF1A signaling pathway
Source: Cell Death Dis. 2022 May 3;13(5):427. doi: 10.1038/s41419-022-04857-y (PMC9065044; doi:10.1038/s41419-022-04857-y)
Supplement: Supplementary file 18 — Supplementary Table 5 [file 41419_2022_4857_MOESM18_ESM.docx]

**Supplementary Table 5. Oligonucleotide sets used for constructs and short hairpin RNAs**

| **Oligo Set** | **Sequences** |
| --- | --- |
| POU6F1-CV186 | 5'-GCTGCAGGTCGACTCTAGAGGATCCATGGATCCTGGAGCCGGGTCA-3' (Forward) |
|  | 5'-ACTGACACACATTCCACAGGCTAGCCTAAGGGATCTGAAAGACGTT-3' (Reverse); |
| pCMV-HA -POU6F1 | 5'-CCGGAATTCGGATGGATCCTGGAGCCGGGTCA-3' (Forward) |
|  | 5'-CCGCTCGAGCTAAGGGATCTGAAAGACGTT-3' (Reverse); |
| sgRNA-CRISPRi-POU6F1 #1 | 5'-CACCGATACATGTAAAAATTTATCAAGG-3' (Forward) |
|  | 5'-AAACCCTTGATAAATTTTTACATGTATC-3' (Reverse); |
| sgRNA-CRISPRi-POU6F1 #2 | 5'-CACCGTTTTGTTTCTGTGTTTATTGGGG-3' (Forward) |
|  | 5'-AAACCCCCAATAAACACAGAAACAAAAC-3' (Reverse); |
| sh-Scb | 5'-CCGGTTCTCCGAACGTGTCACGTCTCGAGACGTGACACGTTCGGAGAATTTTTG-3' (Forward) |
|  | 5'-GATCCAAAAATTCTCCGAACGTGTCACGTCTCGAGACGTGACACGTTCGGAGAA-3' (Reverse); |
| sh-POU6F1 #1 | 5'-CCGGTGAACACCAGCAAGCTGAACCTCGAGGTTCAGCTTGCTGGTGTTCTTTTTG-3' (Forward) |
|  | 5'-GATCCAAAAAGAACACCAGCAAGCTGAACCTCGAGGTTCAGCTTGCTGGTGTTCA-3' (Reverse); |
| sh-POU6F1 #2 | 5'-CCGGTGCCAAGCACACCTGAGTCCCTCGAGGGACTCAGGTGTGCTTGGCTTTTTG-3' (Forward) |
|  | 5'-GATCCAAAAAGCCAAGCACACCTGAGTCCCTCGAGGGACTCAGGTGTGCTTGGCA-3' (Reverse); |
| sh-RORA #1 | 5'-CCGGTGAGCCAGAAGGGATGAACTTTCTCGAGAAAGTTCATCCCTTCTGGCTCTTTTTG-3' (Forward) |
|  | 5'-GATCCAAAAAGAGCCAGAAGGGATGAACTTTCTCGAGAAAGTTCATCCCTTCTGGCTCA-3' (Reverse); |
| sh-RORA #2 | 5'-CCGGTCCGGATGCAGCAGCAGCAGCTCGAGCTGCTGCTGCTGCATCCGGTTTTTG-3' (Forward) |
|  | 5'-GATCCAAAAACCGGATGCAGCAGCAGCAGCTCGAGCTGCTGCTGCTGCATCCGGA-3' (Reverse); |
| RORA-CV186 | 5'-ATGAATGAAGGAGCACCTGGCG-3' (Forward) |
|  | 5'-CCCATCAATTTGCATTGCTGGC-3' (Reverse); |
| pCMV-3Tag-1-RORA | 5'-CGCGGATCCATGAATGAAGGAGCACCTGG-3' (Forward) |
|  | 5'-CCCAAGCTTCTACCCATCAATTTGCATTG-3' (Reverse); |
| pBIFC-POU6F1-VC155 | 5'-CCCGAATTCGCATGGATCCTGGAGCCGGGTCAG-3' (Forward) |
|  | 5'-CGGGGTACCAGGGATCTGAAAGACGTTCAGCTTG-3' (Reverse); |
| pBIFC-RORA-VN173 | 5'-CCCAAGCTTATGAATGAAGGAGCACCTGGCG-3' (Forward) |
|  | 5'-CGGGGTACCGCCCCATCAATTTGCATTGCTGGC-3' (Reverse) |

POU6F1, POU domain, class 6, transcription factor 1; RORA, Retinoid-related orphan receptor alpha.
