# Supplementary material for: POU6F1 cooperates with RORA to suppress the proliferation of lung adenocarcinoma by downregulating HIF1A signaling pathway
Source: Cell Death Dis. 2022 May 3;13(5):427. doi: 10.1038/s41419-022-04857-y (PMC9065044; doi:10.1038/s41419-022-04857-y)
Supplement: Supplementary file 19 — Supplementary Table 6 [file 41419_2022_4857_MOESM19_ESM.docx]

**Supplementary Table 6. Correlation between POU6F1 mRNA expression and clinical parameters of LUAD patients.**

| **Parameter** |  | **Number** | **POU6F1 expression** | | **P value** |
| --- | --- | --- | --- | --- | --- |
|  |  |  | **Low (n=257)** | **High (n=258)** |  |
| Age | <=65 | 238 | 120 | 118 | 0.8602 |
|  | >65 | 258 | 127 | 131 |  |
|  | unknow | 19 | 10 | 9 |  |
| Gender | female | 277 | 152 | 125 | 0.019 |
|  | male | 238 | 105 | 133 |  |
| Tumor stage | I-II | 403 | 214 | 189 | 0.006 |
|  | III-IV | 111 | 42 | 69 |  |
| T stage | T 1-2 | 446 | 231 | 215 | 0.027 |
|  | T 3-4 | 66 | 24 | 42 |  |
|  | unknow | 3 | 2 | 1 | 0.012 |
| N stage | N0+NX | 343 | 185 | 158 |  |
|  | N1 | 172 | 72 | 100 |  |
| M stage | M0+MX | 490 | 249 | 241 | 0.103 |
|  | M1 | 25 | 8 | 17 |  |
