# Supplementary material for: POU6F1 cooperates with RORA to suppress the proliferation of lung adenocarcinoma by downregulating HIF1A signaling pathway
Source: Cell Death Dis. 2022 May 3;13(5):427. doi: 10.1038/s41419-022-04857-y (PMC9065044; doi:10.1038/s41419-022-04857-y)
Supplement: Supplementary file 20 — Supplementary Table 7 [file 41419_2022_4857_MOESM20_ESM.docx]

**Supplementary Table 7. Mass spectrometry (MS) analysis of POU6F1-interacting proteins**

**MS analysis of POU6F1-interacting proteins in A549 cells**

| \| RORA \| \| --- \| \| POU6F1 \| \| KRT18 \| \| ZC3HAV1 \| \| FARP2 \| \| SYF2 \| \| KIF2C \| \| MCRIP2 \| \| DDX41 \| \| SLIRP \| \| DDX50 \| \| RPL17 \| \| UPF3B \| \| STAU1 \| \| AP2B1 \| \| SRSF7 \| \| DDX3X \| \| PHF6 \| \| PUF60 \| \| HLA-A \| \| C4A \| \| DDX17 \| \| HBB \| \| MOGS \| \| RGPD3 \| \| MATR3 \| \| EWSR1 \| \| HNRNPC \| \| MSI2 \| \| CARS1 \| \| HNRNPLL \| \| ANKZF1 \| \| ELOB \| \| FGG \| \| CYCS \| \| MBNL1 \| \| TMEM33 \| \| EIF4E \| \| CSNK1A1 \| \| EXOSC9 \| \| TBCA \| \| AP3M2 \| \| PRRC2C \| \| EZR \| \| RBMS1 \| \| SEPTIN10 \| \| LAMB1 \| \| CALD1 \| \| PPIE \| \| LMO7 \| \| SF3B2 \| \| EEF1D \| \| SEC16A \| \| MYO1C \| \| MRE11 \| \| CPSF6 \| \| NUP160 \|   SEC24C  ANAPC1   \| L1RE1 \| \| --- \| \| STUB1 \| \| LAMTOR2 \| \|  \| | \| PABPC4 \| \| --- \| \| ESRP1 \| \| PUM1 \| \| CRIP2 \| \| ATXN2 \| \| MAN2A2 \| \| ATXN2L \| \| RNPS1 \| \| SLTM \| \| DHX30 \| \| ITIH4 \| \| CP \| \| SRSF2 \| \| HP \| \| SNRPN \| \| NUDT16L1 \| \| RPS15 \| \| DAZAP1 \| \| EXOSC5 \| \| ZNF841 \| \| FBL \| \| SUGP2 \| \| GTPBP1 \| \| IGF2BP3 \| \| KPNA3 \| \| KMT2D \| \| UQCRQ \| \| YKT6 \| \| DHX15 \| \| PRPF4 \| \| SART1 \| \| PLRG1 \| \| ZNF207 \| \| NUDT21 \| \| RAD21 \| \| KPNA6 \| \| DKC1 \| \| DNAJA2 \| \| PRPF40A \| \| SF3B1 \| \| PRKRA \| \| SNRNP200 \| \| ARL6IP5 \| \| BCAS2 \| \| SMNDC1 \| \| FLOT1 \| \| SRP72 \| \| PRPF6 \| \| PTBP3 \| \| AP2A1 \| \| BAG2 \| \| SERPINA1 \| \| A2M \| \| C3 \| \| IGHG1 \| \| IGHG2 \| \| APOA1 \| \| APOC3 \| \| FGA \| \| FGB \|   AKAP8L  SNX9 | \| ORM1 \| \| --- \| \| TF \| \| HPX \| \| CAT \| \| HRG \| \| APOA4 \| \| GPX1 \| \| SNRPB2 \| \| SNRNP70 \| \| VIM \| \| CLTA \| \| CLTB \| \| HNRNPA1 \| \| SNRPA1 \| \| HSPA8 \| \| PABPC1 \| \| HNRNPL \| \| AKR1B1 \| \| GSPT1 \| \| DDX5 \| \| LGALS3 \| \| TCN1 \| \| PCMT1 \| \| SFPQ \| \| EIF4B \| \| EEF1B2 \| \| MSN \| \| DDX6 \| \| U2AF2 \| \| PTBP1 \| \| VARS1 \| \| EEF1G \| \| EEF1D \| \| HNRNPH3 \| \| HNRNPH1 \| \| KIF5B \| \| HSPA4 \| \| PPM1A \| \| SRP14 \| \| EIF4A3 \| \| RPL3 \| \| BUD31 \| \| MTREX \| \| RANGAP1 \| \| CRKL \| \| RPS9 \| \| PRRC2A \| \| RBM25 \| \| RANBP2 \| \| FXR1 \| \| FXR2 \| \| RAB9A \| \| HNRNPA3 \| \| HNRNPM \| \| NCBP2 \| \| HNRNPF \| \| NUP98 \| \| AP2S1 \| \| VCP \| \| AFDN  LSM2  MED31 \| | \| ADAR \| \| --- \| \| SEC13 \| \| HNRNPH2 \| \| RRP1 \| \| NUP107 \| \| WDR5 \| \| HNRNPK \| \| SNRPE \| \| SNRPF \| \| LSM3 \| \| SNRPD1 \| \| SNRPD2 \| \| SNRPD3 \| \| RPS4X \| \| TRA2B \| \| PPP2R2A \| \| SUMO1 \| \| DYNLL1 \| \| HBA1 \| \| RAE1 \| \| SARNP \| \| SRSF3 \| \| PURA \| \| CLTC \| \| U2AF1 \| \| EXOSC10 \| \| OTUD4 \| \| EIF4G1 \| \| YWHAH \| \| EEF1A2 \| \| FMR1 \| \| SRSF1 \| \| DHX9 \| \| GOLGA2 \| \| NCBP1 \| \| AHNAK \| \| CPSF1 \| \| SF3A3 \| \| MYO1E \| \| TARDBP \| \| HNRNPA0 \| \| SRSF9 \| \| SRSF5 \| \| SRSF6 \| \| G3BP1 \| \| PABPC4 \| \| SQSTM1 \| \| TRA2A \| \| EXOSC2 \| \| CIRBP \| \| HNRNPD \| \| SAFB2 \| \| GIT2 \| \| MAP7 \| \| TRIM25 \| \| CAPRIN1 \| \| RBM39 \| \| ITPR3 \| \| SMC1A  PPME1  SUPT16H  TIMM13 \| | \| USP10 \| \| --- \| \| EXOSC7 \| \| EFTUD2 \| \| PLEC \| \| NONO \| \| RBBP5 \| \| SF3B3 \| \| CNN3 \| \| SAFB \| \| SF3B4 \| \| SF3A2 \| \| SF3A1 \| \| TRIP6 \| \| ELAVL1 \| \| DBN1 \| \| SMU1 \| \| LSM12 \| \| SNRPG \| \| HSP90AB2P \| \| ZNF326 \| \| SRSF10 \| \| RRP12 \| \| CRNKL1 \| \| EXOSC6 \| \| CDC40 \| \| HP1BP3 \| \| SRSF11 \| \| HMGB1 \| \| ATAD3B \| \| ZCCHC8 \| \| EDC4 \| \| PRPF8 \| \| LARP1 \| \| FIP1L1 \| \| RPS27L \| \| PHF5A \| \| NUFIP2 \| \| KTN1 \| \| ALYREF \| \| THOC6 \| \| MISP \| \| CHERP \| \| CCAR1 \| \| CCAR2 \| \| LRRC47 \| \| STAG2 \| \| MINK1 \| \| CPSF7 \| \| LSM14A \| \| THOC2 \| \| NUP133 \| \| PSPC1 \| \| DDX1 \| \| TAF15 \| \| RAD50 \| \| UPF1 \| \| ARHGEF2 \| \| ZNF622 \| \| MAGOHB \| \| FAF2 \| \| CCDC124  PFDN2 \| | \| SNRNP40 \| \| --- \| \| DNAJA3 \| \| PTCD3 \| \| MED30 \| \| PGAM5 \| \| FUBP3 \| \| THOC3 \| \| ZFR \| \| RBM14 \| \| PURB \| \| NIBAN2 \| \| CDC5L \| \| TRIR \| \| GRWD1 \| \| MMTAG2 \| \| SF3B5 \| \| SRRT \| \| EIF2A \| \| POLDIP3 \| \| TBL1XR1 \| \| PAIP1 \| \| XRN2 \| \| DHX36 \| \| PNN \| \| WDR26 \| \| MOV10 \| \| EXOSC4 \| \| ATAD3A \| \| TMOD3 \| \| FAM120A \| \| IGF2BP1 \| \| MYOF \| \| CWC15 \| \| ATXN10 \| \| NXF1 \| \| CPSF3 \| \| NUDT5 \| \| RALY \| \| GTF3C4 \| \| AGO2 \| \| PRPF19 \| \| G3BP2 \| \| SRRM2 \| \| SMC3 \| \| RUVBL1 \| \| SF3B6 \| \| PPIL1 \| \| RTCB \| \| LSM5 \| \| RBM7 \| \| YTHDF2 \| \| RBM8A \| \| EXOSC1 \| \| EMG1 \| \| YTHDF3 \| \| BAG6 \| \| CEP43 \| \| PEG10 \| \| AP2M1  IGF2BP2  EMC4  ACIN1 \| | \| RNF114 \| \| --- \| \| WDR47 \| \| PRPF38B \| \| WDR36 \| \| DDX39A \| \| UBE2A \| \| MRPS18B \| \| RBFOX2 \| \| PACSIN2 \| \| MED17 \| \| HNRNPA1P48 \| \| CLPB \| \| IMPDH1 \| \| UROD \| \| RBM15 \| \| MED27 \| \| CTNND1 \| \| ARHGEF7 \| \| CLK2 \| \| HNRNPC \| \| NCOA1 \| \| RTN3 \| \| PPIL3 \| \| EIF4E2 \| \| CUTA \| \| MKRN2 \| \| PALM2AKAP2 \| \| LPP \| \| POP7 \| \| MKRN1 \| \| CDV3 \| \| UBE2D3 \| \| CCNC \| \| STAU2 \| \| COPS6 \| \| UBR5 \| \| U2SURP \| \| PRPF31 \| \| SMC4 \| \| WDR6 \| \| MED22 \| \| EEF1D \| \| EEF1D \| \| ZC3H11A \| \| LSM8 \| \| VPS37B \| \| PAAF1 \| \| QKI \| \| SLC25A10 \| \| LARP4 \| \| RBMS2 \| \| ECI2 \| \| SACM1L \| \| ZC3H14 \| \| SERPINA3 \| \| KLC1 \| \| IBTK \| \| DLAT \| \| YARS2  MED13 \|   DDX20 | \| CERS2 \| \| --- \| \| SRPK1 \| \| JPT2 \| \| ARPP19 \| \| MT1G \| \| AAAS \| \| PDIA3 \| \| MRM3 \| \| TSR1 \| \| GEMIN4 \| \| MAZ \| \| MED19 \| \| NUP85 \| \| MPDU1 \| \| BAG1 \| \| POLRMT \| \| GLYR1 \| \| HS2ST1 \| \| SARS2 \| \| POLD1 \| \| KIF2A \| \| SAP18 \| \| ZNF593 \| \| PPP6C \| \| UBE2C \| \| CHD1 \| \| LSM1 \| \| NKRF \| \| CASC3 \| \| PPM1G \| \| STX7 \| \| POLR1G \| \| AKR7A2 \| \| MED7 \| \| PRC1 \| \| MED14 \| \| AQR \| \| BUB1B \| \| NBN \| \| DUSP11 \| \| MED24 \| \| PSIP1 \| \| MED6 \| \| AP2A2 \| \| LUC7L3 \| \| CPSF4 \| \| ECEL1 \| \| BAG3 \| \| TOP3B \| \| MT-CYB \| \| APOB \| \| HNRNPC \| \| UQCRH \| \| SNRPA \| \| LTA4H \| \| LAMP1 \| \| SON \| \| PTMS \| \| SCP2 \| \| POLR2A  DHRS7 \| | \| PSMB4 \| \| --- \| \| PPIF \| \| ADSS2 \| \| POLR2B \| \| GPD2 \| \| PTDSS1 \| \| PSMB2 \| \| YLPM1 \| \| RIDA \| \| METAP1 \| \| CLTCL1 \| \| MFAP1 \| \| CKS1B \| \| VBP1 \| \| DCAF7 \| \| YPEL5 \| \| NOP14 \| \| MRPS21 \| \| LACTB \| \| RBM10 \| \| REEP5 \| \| KMT2A \| \| AP1B1 \| \| ILK \| \| GOLGA4 \| \| MED21 \| \| NAE1 \| \| SNW1 \| \| THOC5 \| \| TRIM29 \| \| NCOA6 \| \| WTAP \| \| MAPRE2 \| \| SF1 \| \| MED1 \| \| MLF2 \| \| ACTBL2 \| \| HSP90AB4P \| \| SNX5 \| \| SH3BGRL3 \| \| HNRNPK \| \| MRPS2 \| \| LMNA \| \| CEP350 \| \| ATP5F1EP2 \| \| RBM17 \| \| VIRMA \| \| THOC7 \| \| ALKBH5 \| \| WDR82 \| \| GIGYF2 \| \| PAXIP1 \| \| MED12 \| \| MYH14 \| \| WAPL \| \| TMED4 \| \| IRF2BP1 \| \| RBM45 \| \| TBC1D10C  MED31 \|   RRP15 | \| MICU2 \| \| --- \| \| CMTR1 \| \| C12orf29 \| \| FAM98A \| \| LINC01006 \| \| UHMK1 \| \| GEMIN5 \| \| CCDC12 \| \| RNF138 \| \| GEMIN6 \| \| BRI3BP \| \| CTNNBL1 \| \| LARP4B \| \| TFG \| \| DCPS \| \| TRIM11 \| \| MED8 \| \| FYTTD1 \| \| MED15 \| \| WRNIP1 \| \| RANBP9 \| \| CLCC1 \| \| STRBP \| \| RGPD5 \| \| COPS4 \| \| CIZ1 \| \| PAGR1 \| \| MED10 \| \| NDUFAF3 \| \| RPP25 \| \| UTP14A \| \| KIFC1 \| \| TARS2 \| \| YTHDF1 \| \| WDR11 \| \| DPY30 \| \| WDR33 \| \| MED28 \| \| EHD4 \| \| ACBD3 \| \| C8orf33 \| \| PHAX \| \| MED20 \| \| AGO3 \| \| XAB2 \| \| PREB \| \| MED4 \| \| NIT2 \| \| ANLN \| \| DIABLO \| \| LANCL2 \| \| KLC4 \| \| ZCCHC3 \| \| RBM22 \| \| MED9 \| \| OCIAD1 \| \| HYPK \| \| MED11 \| \| CPSF2  TFIP11 \|   ASH2L |
| --- | --- | --- | --- | --- | --- | --- | --- | --- | --- | --- | --- | --- | --- | --- | --- | --- | --- | --- | --- | --- | --- | --- | --- | --- | --- | --- | --- | --- | --- | --- | --- | --- | --- | --- | --- | --- | --- | --- | --- | --- | --- | --- | --- | --- | --- | --- | --- | --- | --- | --- | --- | --- | --- | --- | --- | --- | --- | --- | --- | --- | --- | --- | --- | --- | --- | --- | --- | --- | --- | --- | --- | --- | --- | --- | --- | --- | --- | --- | --- | --- | --- | --- | --- | --- | --- | --- | --- | --- | --- | --- | --- | --- | --- | --- | --- | --- | --- | --- | --- | --- | --- | --- | --- | --- | --- | --- | --- | --- | --- | --- | --- | --- | --- | --- | --- | --- | --- | --- | --- | --- | --- | --- | --- | --- | --- | --- | --- | --- | --- | --- | --- | --- | --- | --- | --- | --- | --- | --- | --- | --- | --- | --- | --- | --- | --- | --- | --- | --- | --- | --- | --- | --- | --- | --- | --- | --- | --- | --- | --- | --- | --- | --- | --- | --- | --- | --- | --- | --- | --- | --- | --- | --- | --- | --- | --- | --- | --- | --- | --- | --- | --- | --- | --- | --- | --- | --- | --- | --- | --- | --- | --- | --- | --- | --- | --- | --- | --- | --- | --- | --- | --- | --- | --- | --- | --- | --- | --- | --- | --- | --- | --- | --- | --- | --- | --- | --- | --- | --- | --- | --- | --- | --- | --- | --- | --- | --- | --- | --- | --- | --- | --- | --- | --- | --- | --- | --- | --- | --- | --- | --- | --- | --- | --- | --- | --- | --- | --- | --- | --- | --- | --- | --- | --- | --- | --- | --- | --- | --- | --- | --- | --- | --- | --- | --- | --- | --- | --- | --- | --- | --- | --- | --- | --- | --- | --- | --- | --- | --- | --- | --- | --- | --- | --- | --- | --- | --- | --- | --- | --- | --- | --- | --- | --- | --- | --- | --- | --- | --- | --- | --- | --- | --- | --- | --- | --- | --- | --- | --- | --- | --- | --- | --- | --- | --- | --- | --- | --- | --- | --- | --- | --- | --- | --- | --- | --- | --- | --- | --- | --- | --- | --- | --- | --- | --- | --- | --- | --- | --- | --- | --- | --- | --- | --- | --- | --- | --- | --- | --- | --- | --- | --- | --- | --- | --- | --- | --- | --- | --- | --- | --- | --- | --- | --- | --- | --- | --- | --- | --- | --- | --- | --- | --- | --- | --- | --- | --- | --- | --- | --- | --- | --- | --- | --- | --- | --- | --- | --- | --- | --- | --- | --- | --- | --- | --- | --- | --- | --- | --- | --- | --- | --- | --- | --- | --- | --- | --- | --- | --- | --- | --- | --- | --- | --- | --- | --- | --- | --- | --- | --- | --- | --- | --- | --- | --- | --- | --- | --- | --- | --- | --- | --- | --- | --- | --- | --- | --- | --- | --- | --- | --- | --- | --- | --- | --- | --- | --- | --- | --- | --- | --- | --- | --- | --- | --- | --- | --- | --- | --- | --- | --- | --- | --- | --- | --- | --- | --- | --- | --- | --- | --- | --- | --- | --- | --- | --- | --- | --- | --- | --- | --- | --- | --- | --- | --- | --- | --- | --- | --- | --- | --- | --- | --- | --- | --- | --- | --- | --- | --- | --- | --- | --- | --- | --- | --- | --- | --- | --- | --- | --- | --- | --- | --- | --- | --- | --- | --- | --- | --- | --- | --- | --- | --- | --- | --- | --- | --- | --- | --- | --- | --- | --- | --- | --- | --- | --- | --- | --- | --- | --- | --- | --- | --- | --- | --- | --- | --- | --- | --- | --- | --- | --- | --- | --- | --- | --- | --- | --- | --- | --- | --- | --- | --- | --- | --- | --- | --- | --- | --- | --- | --- | --- | --- | --- | --- | --- | --- | --- | --- | --- | --- | --- | --- | --- | --- | --- | --- | --- | --- | --- | --- | --- | --- | --- | --- | --- | --- | --- | --- | --- | --- | --- | --- | --- | --- | --- | --- |

**MS analysis of POU6F1-interacting proteins in HEK293T cells**

| SYF2  KIF2C  KRT18  ZC3HAV1  DDX41  MCRIP2  RORA  FARP2  POU6F1  CCNA2  TRIM33  ACIN1  RAB14  PRPS2  SF3A3  UPF1  COPE  NOP58  RPL23  HNRNPA0  KIF2A  SLC25A11  PSMC3  MRPS18B  LAMTOR2  TMEM33  YLPM1  SRSF10  PEF1  THOC7  TFB2M  HDAC1  RPS11  FAM120A  SAFB  LSM12  SLC25A13  ATP5F1C  KHDRBS3  HSPA6  PDCD4  RPS3A  SLC25A5  ALKBH5  GPKOW  RBM4B  PURB  WDR6 | SF3B2  GEMIN5  PPP2R2A  RPL36  RBM45  CWC15  EMC8  EIF5B  RAB3D  EEF1G  CDKN2A  POLR2B  SF3A1  GEMIN6  RTCB  DNAJB1  IGF2BP2  EEF1D  MRPS22  STAU2  USP7  GFPT1  EPRS1  HBS1L  L2HGDH  ELAVL1  RBMS1  PRPSAP1  FUBP3  TRIR  NDUFB6  HDHD5  ASPH  APOC3  EMD  IARS1  YBX1  MCM5  RPL30  ASS1  GNL3  IGF2BP1  TRIM25  TCOF1  RRAS2  ZCCHC3  CAPRIN1  PSPC1 | U2AF1  GALK1  MT-CO2  RANBP2  ATP5PO  SRSF1  SYNCRIP  SF3B5  TOR1AIP1  LCLAT1  UQCRC2  LUC7L2  RPL10  EHD4  EMC4  PSMD1  SMARCE1  CPVL  RAB21  MRPS26  OLA1  MED14  SLC25A22  ILF3  ISY1  UBE2M  YBX3  STAT1  NCBP2  RER1  SUGP2  RPL29  RELA  HNRNPH2  PIP  BCAS2  AQR  PRPF40A  NUSAP1  WDR33  ELAVL2  NUP35  NUP160  RGPD5  C8orf33  AGO1  BAG6  NKRF | CHD5  RBM25  RANBP9  HSD17B12  NUP155  TBL2  ADAR  ARF6  DBT  THOC6  MSI1  MBNL1  PMPCB  AMOT  EEF1D  EEF1E1  EXOSC7  EXOSC2  MRPS2  RAB11FIP1  PTBP1  SNW1  MED8  LARP4B  HNRNPK  SAMM50  PHF5A  ZC3H11A  RHEB  XRN2  HNRNPH1  NDUFA13  RNPS1  DHCR7  MBOAT7  RPP25  SBDS  EFTUD2  HUWE1  ITPR3  PPIE  RBFOX2  CYC1  GINS3  CDC5L  MRPS23  CPSF1  NUP85 | MRPL53  GNA13  NCBP1  MAZ  RPL27  RMND5A  ITPA  AP3D1  POLR2C  PPP6C  RFC4  HNRNPK  KIF5B  PSMD4  CLTA  IRS4  NUP107  HNRNPF  HNRNPL  RUVBL1  RPS15  THOC1  DHX36  HAX1  BUD31  EEF1B2  MKRN2  RANGAP1  XAB2  PTCD3  REL  SRSF5  UPF3B  PABPC1  NUP133  RRP15  HSPA1B  EXOSC6  PRC1  PRPF19  SF3B6  DCAF7  TRA2A  SNRNP40  PSMD2  PRRC2A  AP3M2  MED9 | CPSF3  MED28  CCAR2  PRPF8  RUVBL2  CDC40  NDUFAF4  HDAC2  RBX1  MRPS14  HNRNPA3  MRPS9  NDUFB4  IBTK  OTOP2  MRPS35  HSPA8  RGPD3  ATAD3A  CPSF4  SMARCB1  GTF3C6  RPL10  PSMG3  PLRG1  SMN1  RNF126  HS2ST1  DYNC1LI1  DHX9  PSMD5  GEMIN2  AIMP2  PHF6  MRPS7  FIP1L1  DNAJA3  DNAJA2  THOC3  ZC3H13  HNRNPM  TMEM126A  MED27  BAIAP2L1  RAB9A  MRPL18  MOGS  STUB1 | STAU1  KDM1A  FHL3  CEP350  AKAP8L  LGALS3  MED6  TARS2  ATAD3B  GTF3C5  UTP14A  NUDT16L1  APOA1  CCNC  ERLIN1  POLD1  CCDC85C  EHD1  FAF2  NCOA1  GTF3C4  MAGEB2  EXOSC1  DNAJA1  SNRNP200  POLDIP3  POLR3A  CLTCL1  PRKRA  MYO1E  DDX20  MED15  GIGYF2  EXOSC4  CLTC  FAM120B  CRNKL1  RBM22  EXOSC3  SAFB2  EXOSC5  COQ8A  UBR5  HBB  MATR3  ZFR  HBA1 | KPNA1  C4A  SHROOM3  TSR1  SMARCC1  CLTB  WDR26  POLRMT  BAG4  MCCC2  ZNF622  TRAPPC3  DYNLL1  PPP1R12A  EXOSC10  PPIL4  PSIP1  MPDU1  TPM1  COBLL1  GIT2  SMU1  MRPL58  DCTN1  CALD1  CLPX  GET4  MED20  FYTTD1  THOC2  GOLGA3  CNIH4  SCCPDH  PABPC1  GPX4  BRI3BP  SEH1L  MED31  KDM6A  B2M  MED4  NCOA5  RFC1  DPY30  ECEL1  ARHGAP5  C7orf50  MED12 | WDR5  DNAJB6  EIF4B  FLOT2  CPS1  TMEM200B  MED17  HNRNPC  ZNF326  STAG2  UHMK1  BPIFB1  DDX54  NOP14  MED30  RALY  HRG  HNRNPC  MYH14  FHL2  IGHG1  MED1  HSP90AB4P  BAG5  BAG2  MED19  ASH2L  MED7  SMC1A  KMT2A  C3  GOLGA4  NKAP  LAMB1  STRBP  APOA4  CEP43  MED10  MED22  TFIP11  AKR1C3  RBM7  PAAF1  MED21  HP  ARHGEF7  TBC1D10C  NUDT15 | MED11  NCOA6  TRIM11  LZTS1  CHMP4B  RAD21  TFG  NAT2  PALM2AKAP2  MRE11  CCDC142  AFDN  RBBP5  MLF2  MAN2A2  PAGR1  CIZ1  ZCCHC8  TP53  KMT2D  AFDN  RAD50  PAXIP1  MTREX  TBL1XR1  PPM1F  ACAD9  DHX15  AK6  DAP3  QARS1  RPL21  SMC3  YTHDF1  RABGGTB  NBN  ESRP1  RFC3  PABPC4  SF3B1 |
| --- | --- | --- | --- | --- | --- | --- | --- | --- | --- |
