# Supplementary material for: POU6F1 cooperates with RORA to suppress the proliferation of lung adenocarcinoma by downregulating HIF1A signaling pathway
Source: Cell Death Dis. 2022 May 3;13(5):427. doi: 10.1038/s41419-022-04857-y (PMC9065044; doi:10.1038/s41419-022-04857-y)
Supplement: Supplementary file 21 — Supplementary Table 8 [file 41419_2022_4857_MOESM21_ESM.docx]

**Supplementary Table 8. Putative binding sequences of POU6F1 in the RORA promoter region**

| **Name** | **Score** | **Relative Score** | **Start** | **End** | **Strand** | **Predicted binding sites** |
| --- | --- | --- | --- | --- | --- | --- |
| POU6F1  POU6F1  POU6F1  POU6F1  POU6F1  POU6F1  POU6F1 | 9.168  6.607  6.512  6.470  6.468  6.332  6.131 | 0.9065  0.8604  0.8587  0.858  0.8579  0.8555  0.8518 | 625  296  237  722  971  721  725 | 634  305  246  731  980  730  734 | -  -  -  -  -  +  - | ATAATCAGCA  GTAATGTTTA  CCAATGAGGC  ATGATGATTA  ATCATGACGT  TTAATCATCA  TTCATGATGA |
